# Identification of metabolic genes for the prediction of prognosis and tumor microenvironment infiltration in early-stage non-small cell lung cancer

**DOI:** 10.1515/biol-2022-0091

**Published:** 2022-08-11

**Authors:** Jing Li, Yun Guan, Rongrong Zhu, Yang Wang, Huaguang Zhu, Xin Wang

**Affiliations:** Department of CyberKnife Center, Huashan Hospital, Fudan University, No. 525, Hongfeng Road, Pudong District, Shanghai 200040, China; Department of Rehabilitation, Northern Jiangsu People’s Hospital, Yangzhou, 225001, China

**Keywords:** lung cancer, overall survival, metabolism, immune infiltration, biomarker

## Abstract

Early-stage non-small cell lung cancer (NSCLC) patients are at substantial risk of poor prognosis. We attempted to develop a reliable metabolic gene-set-based signature that can predict prognosis accurately for early-stage patients. Least absolute shrinkage and selection operator method Cox regression models were performed to filter the most useful prognostic genes, and a metabolic gene-set-based signature was constructed. Forty-two metabolism-related genes were finally identified, and with specific risk score formula, patients were classified into high-risk and low-risk groups. Overall survival was significantly different between the two groups in discovery (HR: 5.050, 95% CI: 3.368–7.574, *P* < 0.001), internal validation series (HR: 6.044, 95% CI: 3.918–9.322, *P* < 0.001), GSE30219 (HR: 2.059, 95% CI: 1.510–2.808, *P* < 0.001), and GSE68456 (HR: 2.448, 95% CI: 1.723–3.477, *P* < 0.001). Survival receiver operating characteristic curve at the 5 years suggested that the metabolic signature (area under the curve [AUC] = 0.805) had better prognostic accuracy than any other clinicopathological factors. Further analysis revealed the distinct differences in immune cell infiltration and tumor purity reflected by an immune and stromal score between high- and low-risk patients. In conclusion, the novel metabolic signature developed in our study shows robust prognostic accuracy in predicting prognosis for early-stage NSCLC patients and may function as a reliable marker for guiding more effective immunotherapy strategies.

## 1 Introduction

Lung cancer is one of the most ubiquitous malignancies causing cancer-related deaths worldwide [1,2]. According to the published cancer statistics in the United States in 2018, a total of 234,030 new lung and bronchus cancer cases were estimated to be diagnosed. The incidence in both males and females is ranked second among all cancer types [2]. At the time of primary diagnosis, 80% of all lung cancer patients consist of non-small cell lung cancer (NSCLC), whose current standard treatment is curative resection for its great chances of achieving long-term survival. However, cancer-specific death occurred in nearly 10–44% of operable early-stage NSCLC patients within 5 years after radical resection [3]. Therefore, more reliable predictive models should be developed to identify high-risk early-stage patients who might benefit from a more aggressive follow scheme or therapeutic intervention.

Recently, more and more studies revealed that multi-gene-based signatures possessed a good ability to predict tumor prognosis and survival [4,5,6,7,8,9,10,11,12]. However, a limited robust gene signature has been developed to predict long-term mortality of early-stage NSCLC [13,14,15,16]. In addition, due to the public access to gene expression data and biological process-specific databases, more attention has been paid to constructing a prognostic gene classifier with a unique biological background like immune reaction [17] or epigenetic regulation [18] in NSCLC. Nevertheless, some concerns hamper the prediction power of these prognostic signatures, such as limited sample size and lack of external independent validation.

During the past decades, it has been elucidated that glucose, lipid, and amino acid metabolism are commonly dysregulated in cancer cells and are crucial to sustain cancer cell growth and proliferation [19]. Like highly proliferating normal cells, cancer cells require a constant supply of ATP and anabolic precursors to promote crucial biochemical processes, including DNA synthesis, protein, and fatty acid synthesis, post-translational modification of proteins, membrane formation and reassembly, vesicular transport of intracellular cargos, and endocytosis [20]. Previous studies have revealed that critical enzymes or transporters mediating cellular metabolism play fundamental roles in regulating tumor cell progression and therapeutic response [21,22,23,24]. In addition, a lot of enzymes and transporters have been proven as prognostic biomarkers in NSCLC [25,26]. However, no previous studies analyzed these metabolic genes comprehensively to evaluate the prognostic value of the entire gene set mediating cell metabolism. Therefore, we hypothesized that the metabolism-related gene set-based prognostic signature might be of concrete predictive value in helping to identify high-risk patients with poor prognoses.

In the present study, a total of 1,161 early-stage NSCLC patients were identified from Gene Expression Omnibus (GEO) database. Based on a specific sample-splitting approach and Cox regression analysis, a 42 metabolism-related genes-based metabolic signature was established from the discovery set and successfully validated in the internal validation cohort and another two independent cohorts. This signature may assist in identifying the subgroup of NSCLC patients at high risk of poor survival.

## 2 Methods

### 2.1 Preprocessing of microarray data

Raw microarray colon cancer data sets were obtained from the GEO database (http://www.ncbi.nlm.nih.gov/geo/) and were normalized using Robust Multichip Average [27]. All data sets were produced by the Affymetrix platform (U133 plus 2.0 or U133A). All probes were mapped based on their own EntrezGeneID. When multiple probes were mapped to the same EntrezGeneID, the mean value was used to represent its average expression level.

### 2.2 Datasets selection

The inclusion criterion for datasets was as follows: (i) all data sets were created by Affymetrix platform (U133 plus 2.0 or U133A); (ii) sample size was set as over one hundred; and (iii) patients should be pathologically diagnosed with NSCLC. Datasets missing necessary clinicopathological or follow-up data were excluded. Finally, NSCLC data sets of GSE31210, GSE30219, GSE37745, GSE50081, and GSE68465 were identified in this study to construct and validate the prognostic value of gene signature. All identified patients were re-staged according to the 8th TNM staging system. GSE31210, GSE37745, and GSE50081 were combined as a meta data set and were randomly divided into discovery (*n* = 286) and internal validation set (*n* = 286). GSE30219 and GSE68465 were two independent external validation sets. ComBat method was used to remove the batch effects when combing different data sets, and this method was implemented in the SVA R package. The patients from data sets were further filtered based on tumor stage, and early-stage (TNM I–II) patients were finally identified.

### 2.3 Metabolism-related gene set

A total of 5,557 genes corresponding to human metabolic enzymes and transporters were downloaded from The Human Metabolome Database (HMDB) (http://hmdb.ca/), of which 4,152 genes were included in the microarray probes of the Affymetrix platform.

### 2.4 Identification of prognostic metabolism-related genes

**Figure 1:**
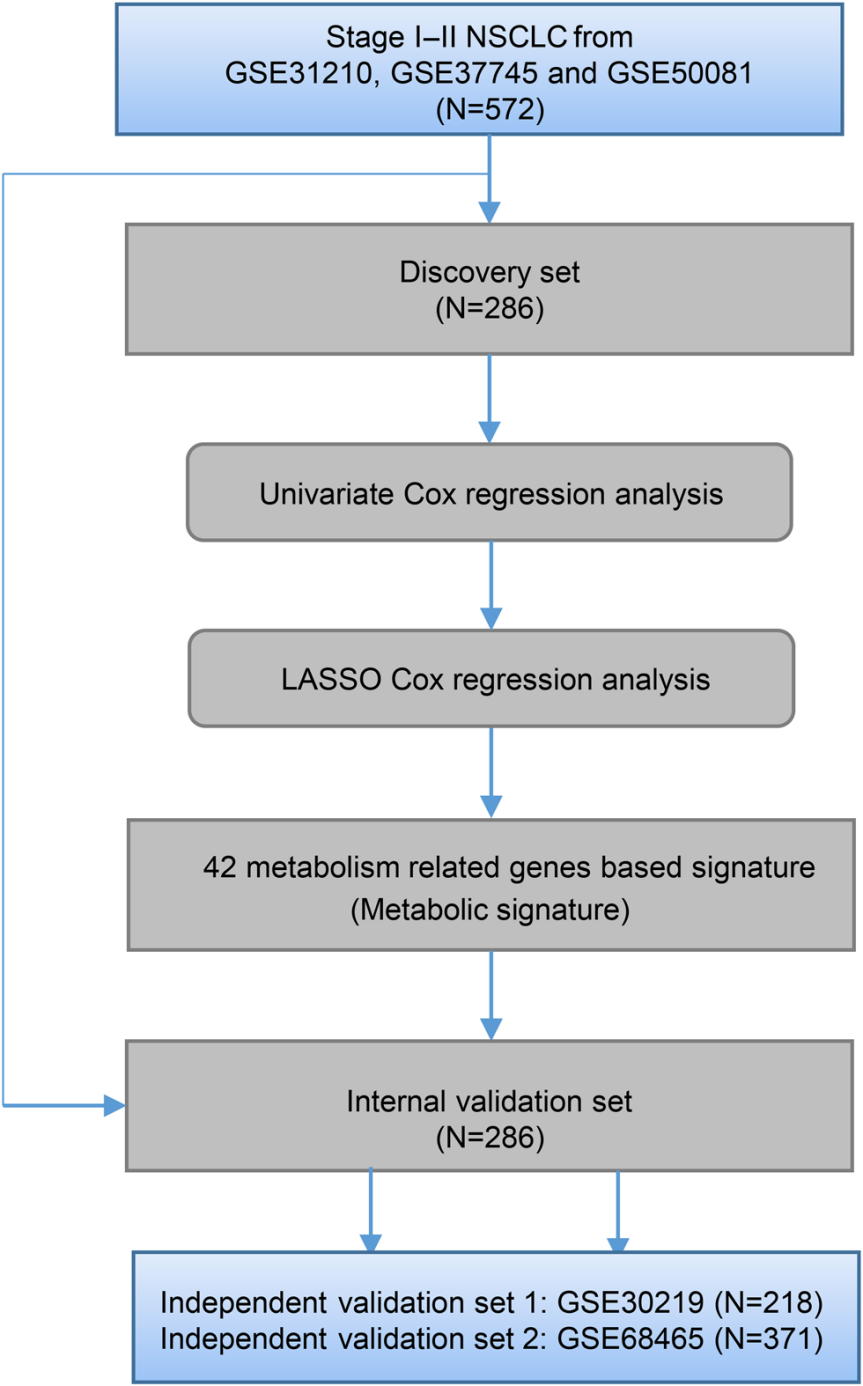
The flowchart of our study.

The overall flowchart of our study is shown in Figure 1. In the discovery data set, univariate Cox survival analysis was first used to filter the potential prognostic genes significantly associated with OS. The least absolute shrinkage and selection operator method (LASSO) Cox regression model at 10-fold cross-validation was then performed to identify the most useful metabolism-related genes.

### 2.5 Development of risk score and statistical analyses

Using LASSO Cox regression, we identified a panel of genes and constructed a classifier based on multi-mRNAs to predict OS of early-stage lung cancer in the discovery cohort. A standard risk score calculation formula was used to generate each patient’s risk score by combining the gene expression value and the corresponding LASSO Cox regression coefficients (β). Based on the median risk score developed in the discovery cohort, individuals from different cohorts were categorized into high-risk and low-risk subgroups. The risk score formula was defined as the following:

{\rm{Risk\; score}}=\hspace{1em}\mathop{\sum }\limits_{i=1}^{n}{{\rm{\exp }}}_{i}* {\beta }_{i}.]

### 2.6 Gene set variation analysis (GSVA) of the developed metabolic signature

By using the non-parametric and unsupervised method, GSVA is widely accepted as a reliable method to estimate signaling pathway activity based on RNA expression data. Therefore, we used “GSVA” R packages to compare the difference in the activity of biological signaling pathways between low- and high-risk samples. Gene sets information for GSVA processing was downloaded from the MSigDB database (https://www.gsea-msigdb.org/gsea/index.jsp).

#### 2.6.1 Cibersort

To characterize the 22 kinds of immune cell compositions (including B cells naïve, B cells memory, plasma cells, T cells CD8, CD4, naïve T cells, CD4 memory resting, T cells CD4 memory activated, T cells follicular helper, T cells regulatory (Tregs), T cells gamma delta, NK cells resting, NK cells activated, monocytes, macrophages M0, macrophages M1, macrophages M2, dendritic cells resting, dendritic cells activated, mast cells resting, mast cells activated, eosinophils, and neutrophils) in each sample. We used the CIBERSORT algorithm (http://cibersort.stanford.edu/) to calculate the infiltration level of each immune cell based on the gene expression profiles.

### 2.7 Estimation of stromal and immune score

The fractions of stromal and immune cells reflected by the stromal and immune score were inferred in each lung cancer sample using the ESTIMATE algorithm (https://sourceforge.net/projects/estimateproject/).

### 2.8 Statistical analyses

Wilcoxon rank-sum test was performed for data with skewed distribution, or Student’s test was conducted for data with normal distribution to compare the difference between groups. Kaplan–Meier method was used to plot the survival curves with a log-rank test comparing the differences between survival curves. The independent OS prediction ability of the risk score was tested via multivariate Cox regression analyses and data stratification analyses. Time-dependent receiver operating characteristic curve (ROC) analysis was utilized to probe the prognostic or predictive accuracy of each parameter and the signature as well. Decision curve analysis (DCA) examined the theoretical relationship between the threshold survival probability at the 5 years of OC and the relative value of false-positive and false-negative results to determine the clinical utility of the metabolic signature. All statistical R software (version 4.0.5, www.r-project.org) was adopted for all statistical analyses. All statistical tests were 2-sided, and a *P* value <0.05 was considered of statistical significance. When selecting potential prognostic genes that are significantly associated with OS, a *P* value less than 0.001 was set as a threshold to make ideal data reduction.

## 3 Results

### 3.1 Construction of metabolic signature from the discovery set

**Figure 2:**
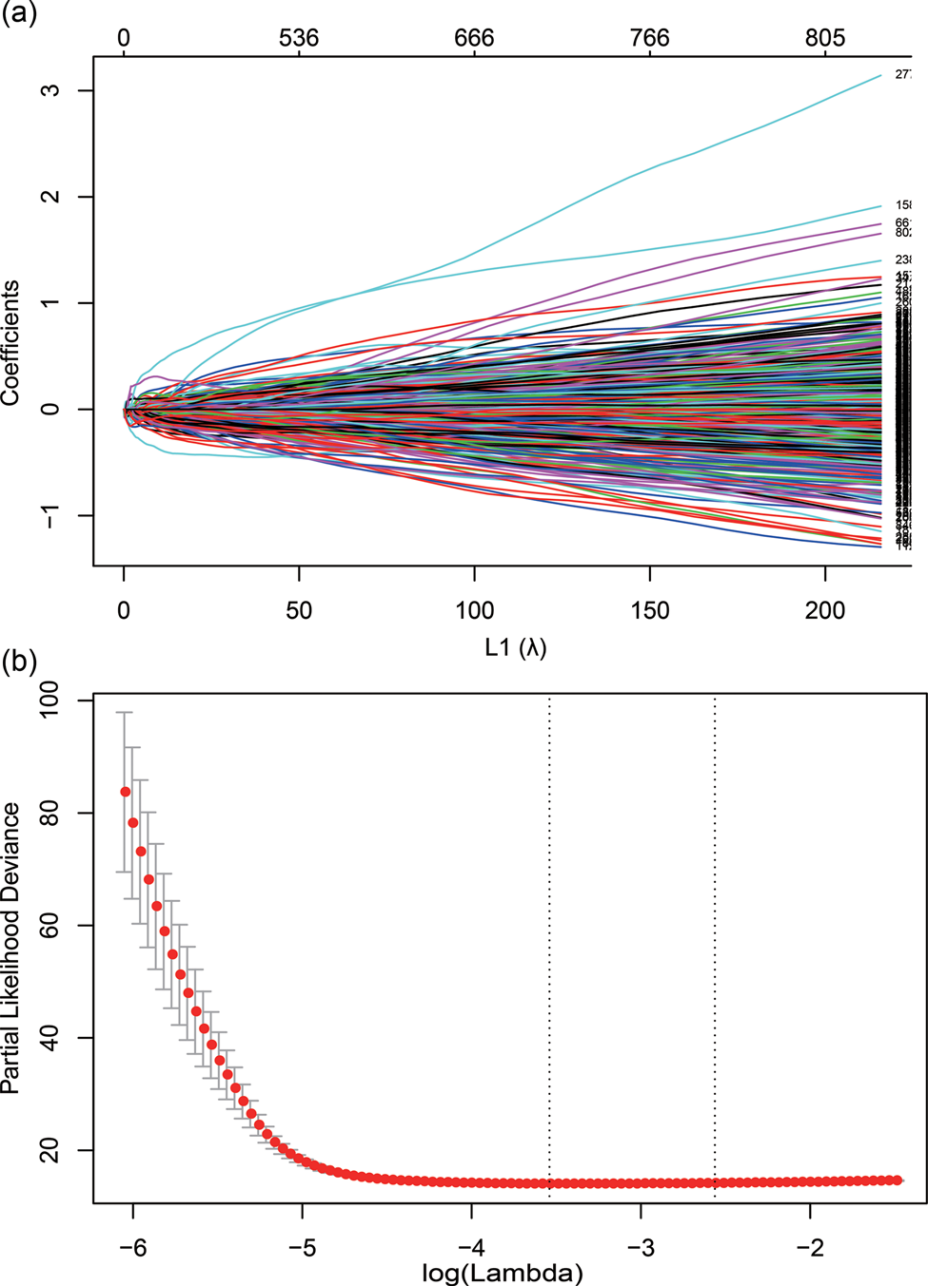
(a) LASSO coefficient profiles of the 3,493 OS-associated metabolic genes. (b) Tuning parameter (l) selection in the LASSO model used 10-fold cross-validation via minimum criteria. Abbreviations: LASSO, least absolute shrinkage and selection operator; OS, overall survival.

A total of 1,161 early stage (Stage I/II) patients were finally identified, which included 218 patients from GSE30219, 226 patients from GSE31210, 165 patients from GSE37745, 181 patients from GSE50081, and 371 patients from GSE68465. Among 5,557 metabolism-related genes from the HMDB database, 4,152 genes were measured by all platforms. In the discovery set, a univariate Cox survival analysis was performed and found that 3,493 metabolism-related genes were significantly associated with OS with *p* value less than 0.001 (Table S1). LASSO regression model was further conducted and the minimize λ method resulted in 42 prognostic epigenetic genes at 10-fold cross-validation (Figure 2). Finally, a risk score was calculated based on the expression value of the 42 genes and risk regression coefficients for each patient (Table S2).

### 3.2 The prognostic value of metabolic signature in the discovery, internal validation, and external validation sets

**Figure 3:**
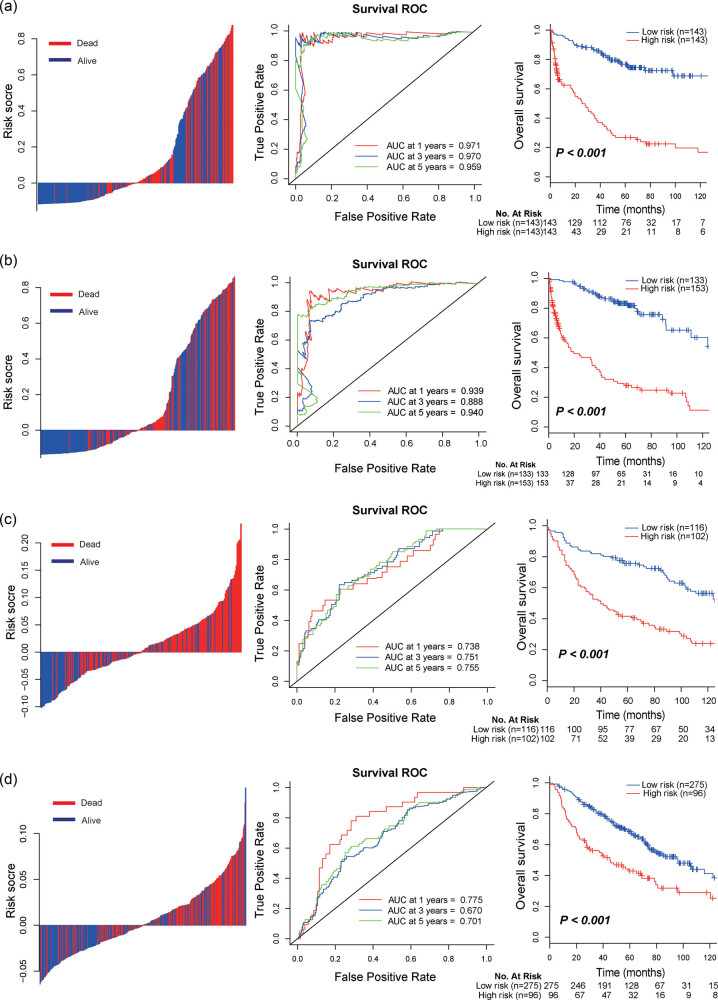
Distribution of risk score (left panel), time-dependent ROC curves at 1, 3, and 5 years (middle panel), and Kaplan–Meier survival analysis between patients at low and high risk of death (right panel) in discovery set (a), internal validation set (b), independent validation set 1 (c) and independent validation set 2 (d). Abbreviations: ROC, receiver operating characteristic curve.

The distribution of risk scores and OS status are depicted in Figure 3a (left panel). The chance of death raised steadily, and the score increased. Time-dependent ROC analyses at the 1 , 3, and 5 years were performed to evaluate the prognostic accuracy of the metabolic signature (Figure 3a, middle panel). The 1, 3, and 5-year OS rates of those with low-risk scores were 94.4, 85.8, and 77.5%, compared with 62.9, 40.2, and 26.4% for patients in high-risk group, respectively (HR: 5.050, 95% CI: 3.368–7.574, *P* < 0.001, Figure 3a, right panel).

We then carried out the same analyses in the internal validation cohort. The prognostic score showed the same clinical significance as in the discovery set. The 1-year, 3-year, and 5-year RFS were 98.5, 89.8, and 83.3% for the low-risk group, and 58.7, 41.4, and 28.0% for the high-risk group (HR: 6.044, 95% CI: 3.918–9.322, Figure 3b, right panel)).

To confirm that the metabolic signature had a similar prognostic value in different populations, we validated the performance of the metabolic signature in another two independent data sets, GSE30219 and GSE68465. Using the established cutoff point, the metabolic showed good performance in predicting OS in both two validation cohorts (GSE30219: HR: 2.059, 95% CI: 1.510–2.808, Figure 3c, middle panel; GSE68465: HR: 2.448, 95% CI: 1.723–3.477, *P* < 0.001, Figure 3d, middle panel). Moreover, the metabolic signature can separate patients into two risk groups with significantly different OS (Figure 3c–d, right panel).

### 3.3 Independence and accuracy of the signature in OS prediction

**Figure 4:**
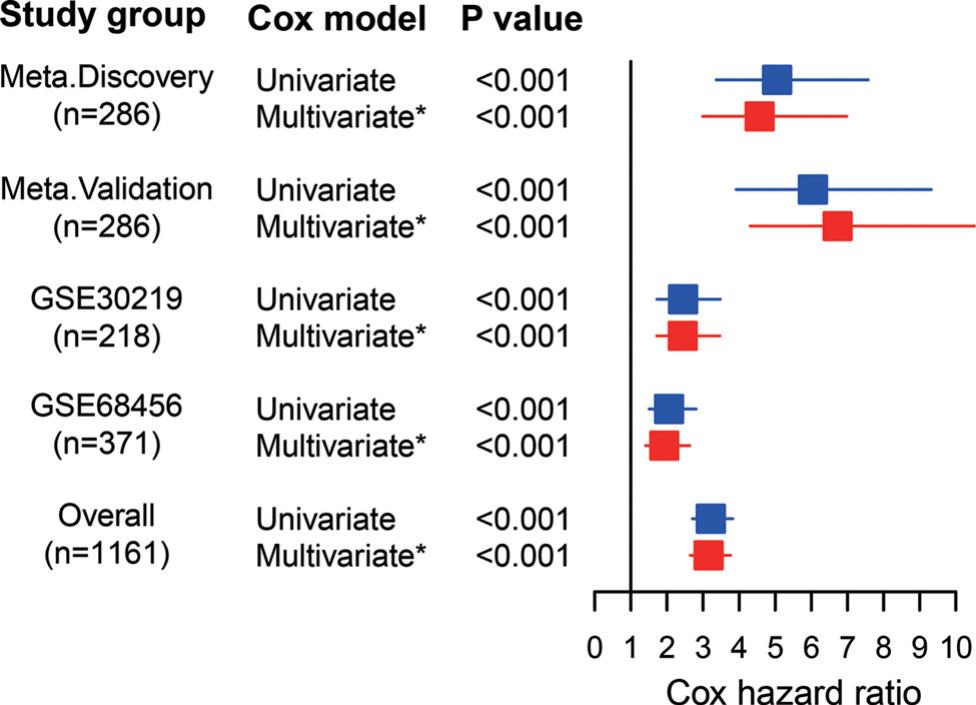
Forest plot of the univariate and multivariate Cox regression analysis in predicting OS: Adjusting age, sex, TNM stage, and histology. Abbreviations: OS, overall survival.

**Figure 5:**
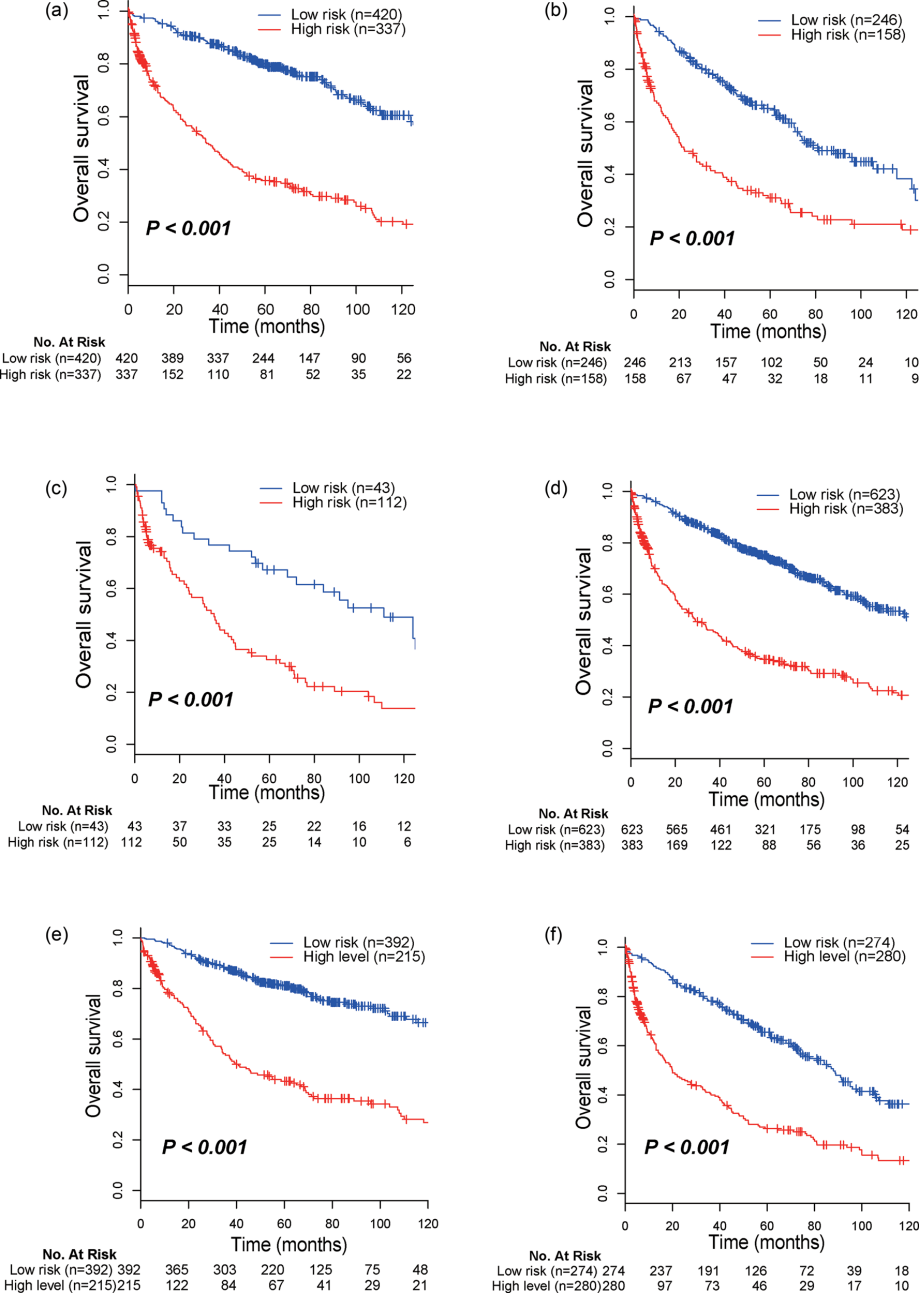
Kaplan–Meier survival analysis for patients based on the metabolic signature stratified by clinicopathological risk factors. (a) stage I, *P* < 0.001; (b) stage II, *P* < 0.001; (c) squamous cell carcinoma, *P* < 0.001; (d) non-squamous cell carcinoma, *P* < 0.001; (c) age <65, *P* < 0.001; (d) age ≥65, *P* < 0.001.

After multivariate analyses adjusted by clinicopathological parameters, including age, sex, histology, and AJCC TNM stage, the metabolic signature remained a powerful and independent prognostic factor in all sets (Figure 4). Further stratification analysis showed that the signature was a statistically significant prognosis predictor too in Stage I (Figure 5a), Stage II (Figure 5b), and patients diagnosed with squamous cell carcinoma (Figure 5c) or non-squamous cell carcinoma (Figure 5d), patients with age <65 (Figure 5e) or ≥65 (Figure 5f).

**Figure 6:**
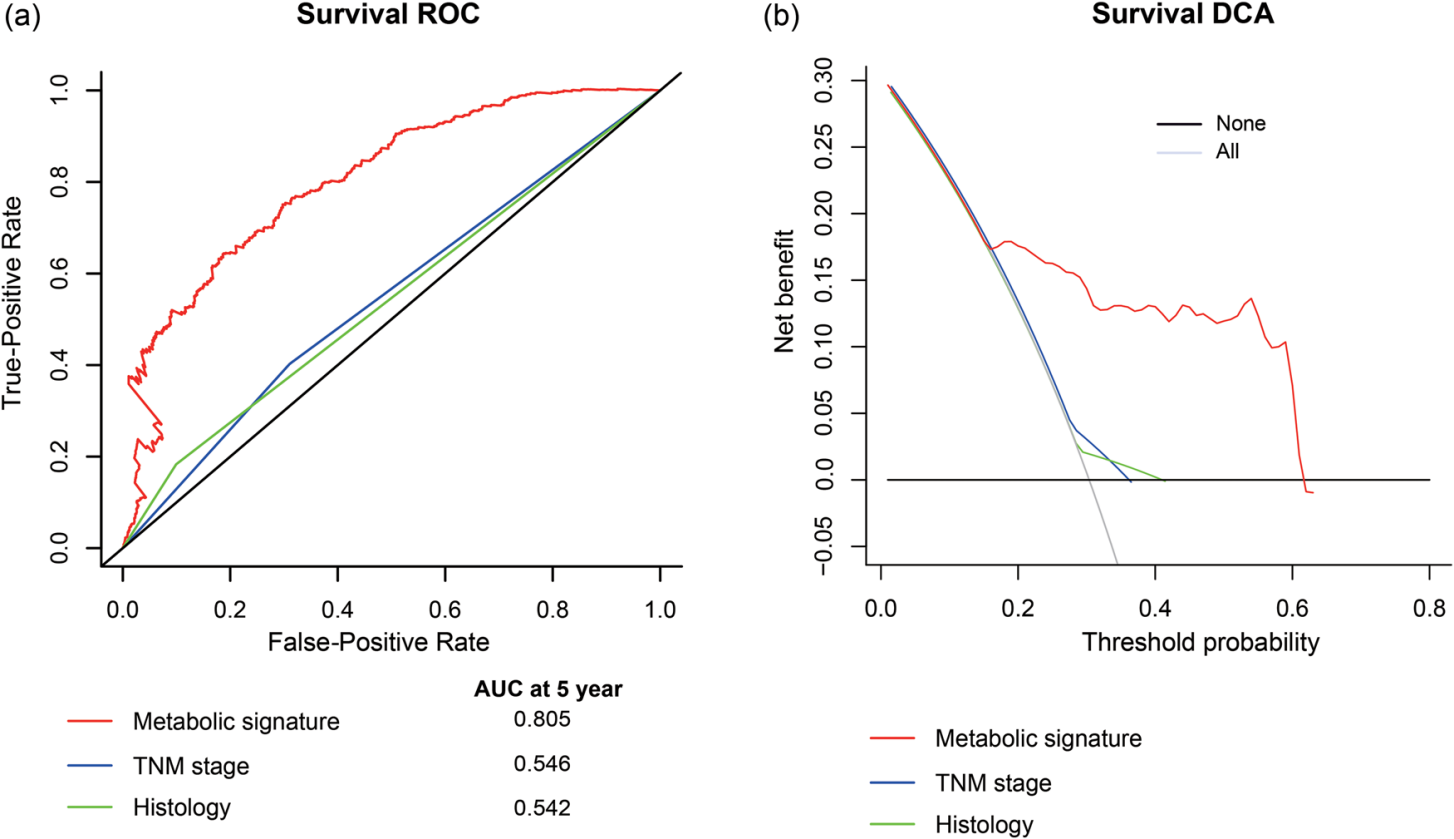
Time-dependent ROC curves at 5 year compare the prognostic accuracy in predicting OS of the metabolic signature with clinical factors (a) in the entire cohorts with stage I-II lung cancer (*N* = 1,161). DCA at 60 months for the tumor stage, histology and metabolic signature (b). The *y*-axis measures the net benefit.

To further verify the high efficacy of this signature in predicting OS, time-dependent ROC analysis demonstrated that the metabolic signature (AUC = 0.805) had significantly better prognostic accuracy than the tumor stage at 5 years (Figure 6a). In addition, the DCA analysis suggested that using the metabolic signature to predict OS brought notably more benefit than either the treat-all-patients scheme or the treat-none scheme, while the tumor stage or histology added strikingly less benefit (Figure 6b).

### 3.4 The metabolic signature is associated with immune cell infiltration

**Figure 7:**
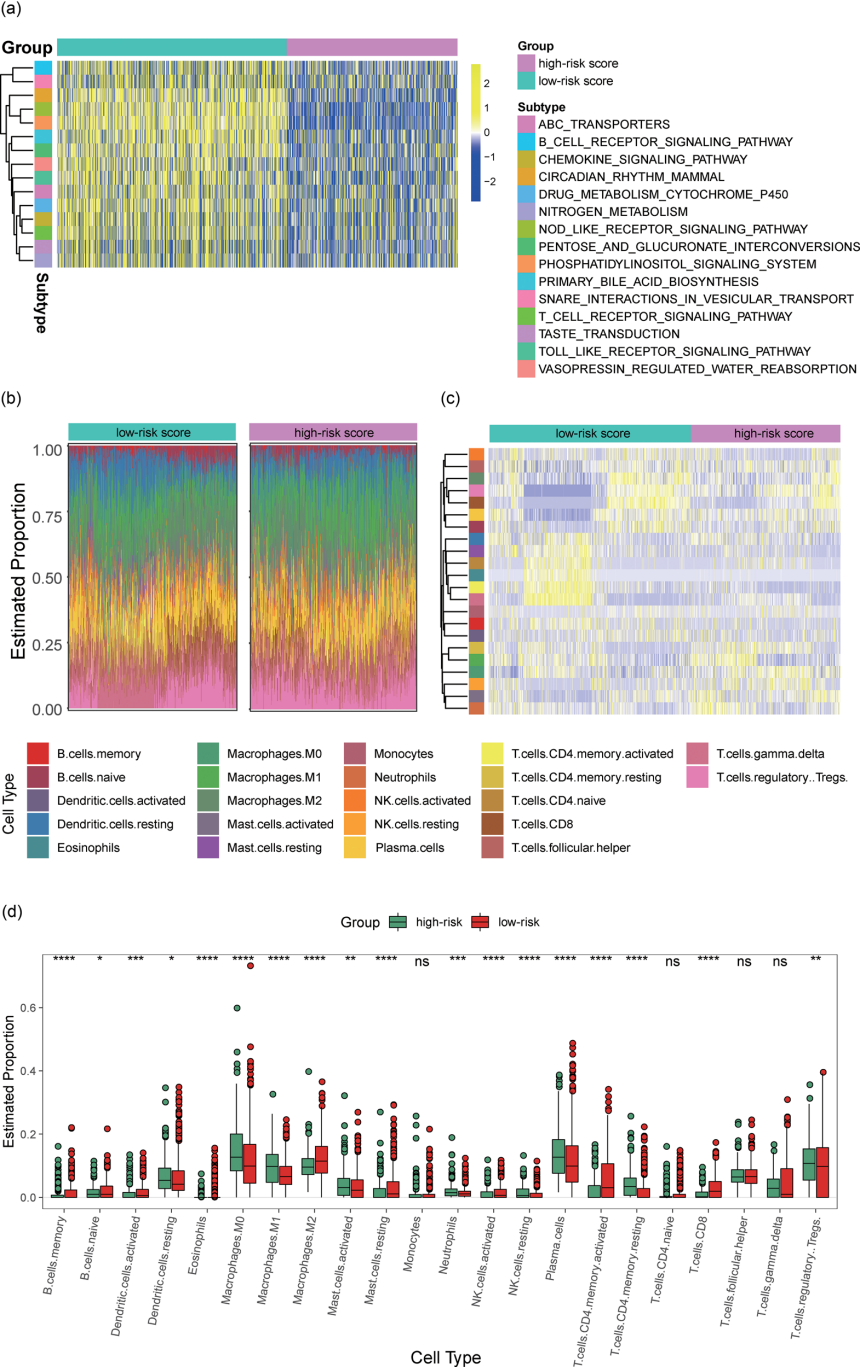
(a) GSVA enrichment analysis showing the activation states of signaling pathways in low- and high-risk patients stratified by metabolic gene-set-based signature. (b) The proportions of different immune cell components in NSCLC cancer samples from low- and high-risk groups. (c) Heatmap for the infiltration levels of immune cells in the high- and low-risk patients. (d) Box plots for the differences in infiltration levels of immune cells between high- and low-risk patients. Abbreviations: GSVA, gene set variation analysis; OS, overall survival; and NSCLC, non -mall cell lung cancer.

To characterize the biological processes within this 42-gene-based metabolic signature, we performed GSVA analysis, which indicated that the low-risk group is associated with immune activation biological processes, including T-cell and B-cell receptor signaling pathways, Nod-like receptor signaling pathway, and Chemokine signaling pathway (Figure 7a). To further explore the difference in immune cell infiltration between the two risk groups, we performed a CIBERSORT analysis, and the proportions of 22 kinds of immune cell components in cancer tissues are visualized in Figure 7b. Heatmap was then used to plot the difference in the immune cell infiltrations between high- and low-risk cancer samples (Figure 7c). Compared with the high-risk group, the infiltration levels of B-cell memory and naive, dendritic cells activated, macrophages M2, NK cells activated, CD4 and CD8 T cells (Figure 5d). Instead, the regulatory T cells functioning suppressing effect on other T-cell subsets was highly enriched in high-risk samples.

### 3.5 Potential of the metabolic signature as an indicator of immunotherapy response in early-stage NSCLC patients

**Figure 8:**
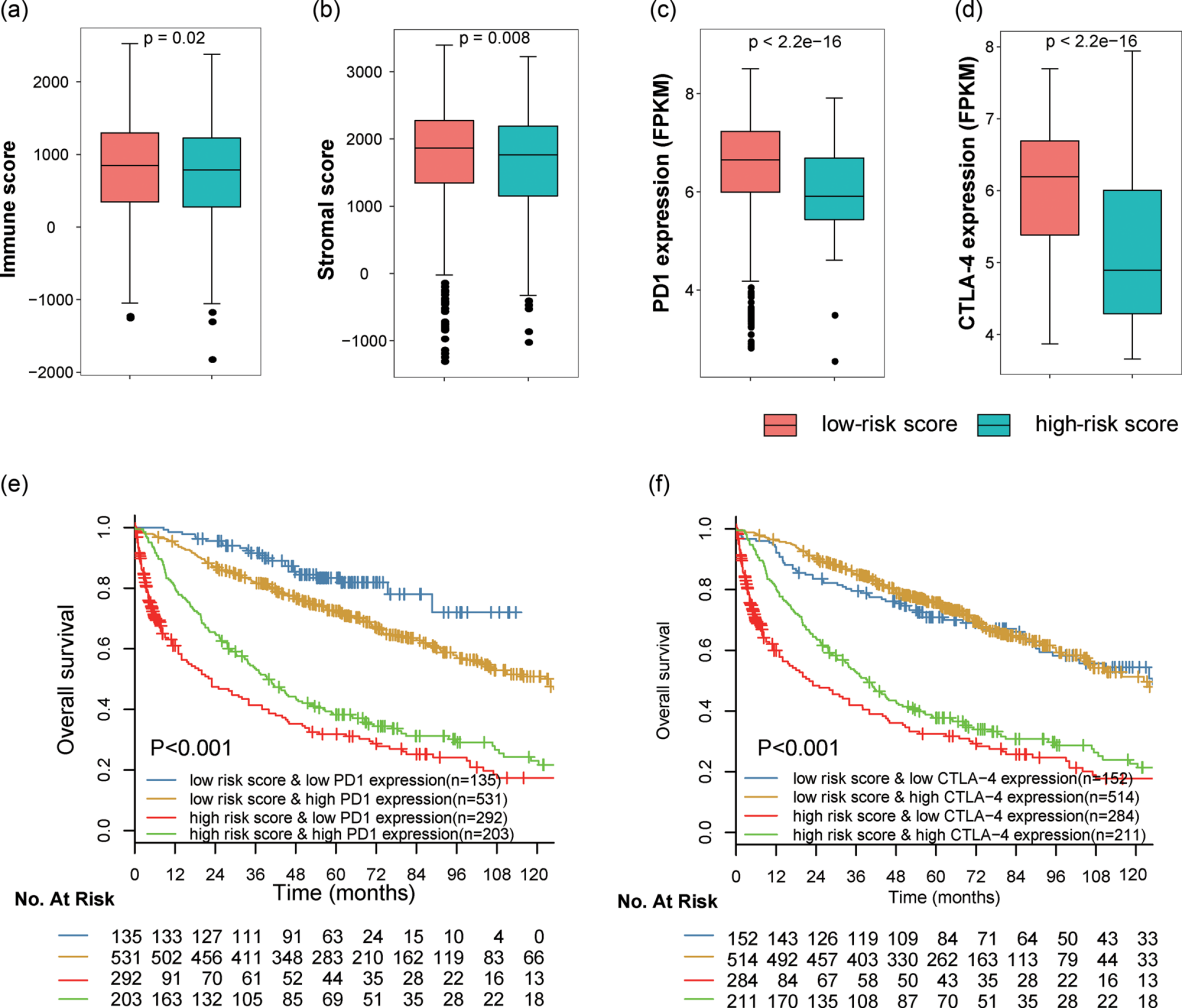
Box plots for the (a) immune and (b) stromal scores in the high- and low-risk groups. Box plots for the expression of immune checkpoints (c) PD-1 and (d) CTLA-4 in patients with high- and low-risk scores. Kaplan–Meier survival curves of overall survival among four patient groups stratified by the metabolic risk scores and PD-1 (e) and CTLA-4 (f). Abbreviations: PD1, programmed cell death-1; CTLA-4, cytotoxic T- lymphocyte Antigen 4.

According to the difference in immune cell infiltration between low- and high-risk samples, we hypothesized that the developed metabolic gene-set-based signature might be an indicator of immunotherapy response. Tumor purity and immune checkpoint inhibitor (ICI) gene expression are established indicators for the immunotherapy response. We then explored the correlation between metabolic signature, tumor purity, and ICI gene expression. As a result, immune and stromal scores in low-risk samples were significantly higher than in high-risk samples (Figure 8a and b). Next, the expression levels of ICI genes (programmed cell death [PD-1] and cytotoxic T-lymphocyte Antigen 4 [CTLA-4]) between patients with low- and high-risk scores were compared. It was found that high-risk patients tend to show low PD-D1 and CTLA-4 expression (Figure 8c and d), suggesting a possible weak immunotherapy response in the high-risk patient group.

To further test whether the microenvironment immune infiltration characterized by metabolic signature has an impact on survival in patients with similar ICI gene expression levels, stratified survival analysis was performed. It revealed that patients with low-risk scores and low PD-1 levels had notably better survival than those with a high-risk score and low PD-1 level (Figure 8e, *p* < 0.001), and patients with low-risk scores and high PD-1 also were associated with prolonged survival relative to those patients with high-risk score and high PD-L1 (Figure 6e, *p* < 0.001). A similar result was successfully repeated by using risk score and CTLA-4 expression level. When stratifying patients with high-risk scores based on ICI expression, we also observed that ICI gene expressions were associated with noticeable survival differences in patients with high-risk scores. However, survival differences were totally reversed for low- and high-risk patients when stratifying patients based on PD1 expression. In addition, survival differences were not present for patients with low-risk scores when stratifying patients with CTLA-4 expression. Together, these results revealed the potential of metabolic gene-set-based signature as a predictive indicator of treatment response to ICI immunotherapy.

## 4 Discussion

Surgery is the optimal treatment to cure lung cancer. However, nearly 50% of patients with NSCLC might experience relapse and cancer-specific death in spite of curative resection [28,29]. TNM staging indicates the seriousness and recurrence potential of primary lung carcinoma [30,31]. Nevertheless, even patients diagnosed with the same TNM stage could show distinct recurrence potentials after curative resection. Consequently, the current TNM staging system has limitations in clinical practice. In this study, a novel prognostic signature based on 42 metabolic genes was developed to improve the prediction of OS for NSCLC patients after surgical resection. By applying the metabolic signature to the meta-discovery set patients, a clear separation was observed in the survival curves between low- and high-risk patients. And it was successfully validated in the internal meta-validation set and the two external independent validation cohorts of GSE30219 and GSE68465, indicating the good reproducibility of this signature in early-stage NSCLC. In addition, the time-dependent ROC and DCA curves at the 5 years suggested that this metabolic signature has the most significant accuracy and clinical utility in predicting mortality after the initial resection of NSCLC. Therefore, our study identified a novel metabolic signature that could help identify patients with high mortality risk.

Previous studies have reported different prognostic signatures for patients with early-stage lung cancer. Nevertheless, some concerns hamper the prediction power of these prognostic signatures, such as limited sample size, and lack of external independent validation. Based on The Cancer Genome Atlas and GEO cohorts, Li et al. constructed a prognostic signature of 25 gene pairs consisting of 40 unique genes in early-stage NSCLC, while this model achieved an average predictive accuracy with a C-index of 0.64 [8]. Wu et al. reported an immune-related genes-based signature in early-stage lung cancer. However, the 5-year AUC of the prognostic signature reached only 0.62 in predicting OS in the validation cohort [17]. In this study, the developed metabolic signature embraced a robust predictive power in the discovery set and the overall cohort.

NSCLC is a type of immunogenic cancer. The spontaneous anticancer immune response might help increase survival duration while the immune escape may cut down survival [32]. Recently, several immune-related treatment schemes such as immune checkpoint inhibition, vaccination, and antigen-specific active immunotherapy have been developed in NSCLC [32]. The aim of immunotherapy is to activate the immune-suppressive networks in the tumor microenvironment. Several immune-related markers have been utilized for predicting the responses to immunotherapy [33]. The expression of immune checkpoint genes such as PD1 is currently available biomarkers in clinical practice. However, only a small subset of patients with NSCLC are responders for ICIs because of potential innate tumor heterogeneity. In addition, the immune infiltrates in the TME are increasingly recognized to be associated with immunotherapy response. Our study revealed that the metabolic signature was in relation to infiltrations of B-cell memory and naive, dendritic cells activated, macrophages M2, NK cells activated, CD4, and CD8 T cells, indicating the crosstalk between these metabolic genes expression and immune cells. Tumors positive for PD-1 usually exhibit a higher response to immune checkpoint inhibition therapy. Here, our data showed that the low-risk NSCLC patients had higher PD-1 expression in comparison to those with high risk. These findings indicated that the complex interplay between immune infiltrate and immune checkpoint gene in the tumor immune microenvironment has an impact on the prognosis of NSCLC patients.

Although we successfully developed a model for cancer prognosis prediction through a biology-driven approach in large NSCLC cases, some limitations in our study need to be addressed. First, the data of several other crucial clinicopathological features and therapy strategies are not available in the GEO database. Thus, we cannot adjust these factors when building the signature. Second, our study was based on the data from publicly available data sets, which may have some inherent limitations of a retrospective study, and thus, future prospective studies might be warranted to substantiate our results. Lastly, mechanisms of the identified metabolic genes on the progression of early-stage NSCLC still need further investigation.

In summary, our study revealed that the metabolic prognostic signature could distinguish early-stage NSCLC patients with low mortality risk from those with high death risk, regardless of TNM stage and histotype. This novel metabolic signature developed in our study embraces robust prognostic accuracy in predicting prognosis for early-stage NSCLC patients and may function as a reliable marker for guiding more effective immunotherapy strategies.

## Supplementary Material

Supplementary Figure
