# Supplementary material for: Identification of metabolic genes for the prediction of prognosis and tumor microenvironment infiltration in early-stage non-small cell lung cancer
Source: Open Life Sci. 2022 Aug 11;17(1):881–92. doi: 10.1515/biol-2022-0091 (PMC9372707; doi:10.1515/biol-2022-0091)
Supplement: Supplementary Figure [file biol-2022-0091-sm.pdf]

Supplementary material

Table S1: Univariate Cox regression analysis of the whole metabolic genes in predicting OS in discovery set

Gene	*P* value	HR	lower 95% CI	upper 95% CI
A1CF	0.001982	0.79212	0.683371	0.918175
A2M	<0.001	0.346029	0.289502	0.413593
A4GALT	1.16E-13	1.540236	1.37419	1.726345
AACS	<0.001	0.463136	0.398876	0.537749
AADAC	9.66E-15	0.76258	0.712016	0.816735
AAK1	<0.001	0.175992	0.125649	0.246506
AANAT	1.07E-09	1.52029	1.328778	1.739404
AARS	<0.001	0.426751	0.368057	0.494805
AASDHPPT	<0.001	0.387913	0.332517	0.452537
AASS	<0.001	0.563323	0.508199	0.624427
ABAT	<0.001	0.548645	0.496278	0.606537
ABCA1	<0.001	0.418888	0.351961	0.498542
ABCA7	<0.001	0.395924	0.336621	0.465674
ABCB1	<0.001	0.427556	0.367688	0.497172
ABCB11	4.97E-07	1.563187	1.313335	1.860573
ABCB4	<0.001	0.458183	0.389224	0.53936
ABCB6	9.07E-11	1.702613	1.449537	1.999875
ABCB7	<0.001	0.404295	0.348961	0.468404
ABCC1	7.30E-13	0.572125	0.491172	0.666422
ABCC2	2.43E-06	1.175756	1.099204	1.257641
ABCC3	<0.001	0.618446	0.553574	0.690919
ABCC4	<0.001	0.427742	0.368236	0.496865
ABCC5	<0.001	0.541474	0.477994	0.613384
ABCC8	4.44E-16	1.486452	1.350802	1.635725
ABCF1	<0.001	0.377239	0.315072	0.451672
ABCG2	<0.001	0.523535	0.468092	0.585544
ABHD10	<0.001	0.471158	0.415011	0.534901
ABHD4	<0.001	0.311743	0.25461	0.381696
ABHD5	<0.001	0.435067	0.381879	0.495663
ABHD6	<0.001	0.248827	0.194474	0.318371
ABI1	<0.001	0.427116	0.371667	0.490839
ABL1	<0.001	0.417322	0.355804	0.489478
ABL2	<0.001	0.313183	0.257445	0.380988
ABO	0.022891	1.252477	1.031687	1.520518

(*Continued*)

Table S1: *Continued*

Gene	*P* value	HR	lower 95% CI	upper 95% CI
ABR	<0.001	0.30323	0.249821	0.368057
ACAA1	<0.001	0.311988	0.25625	0.379849
ACAA2	2.87E-05	0.617	0.492082	0.773628
ACACA	<0.001	0.394786	0.332936	0.468125
ACACB	<0.001	0.263108	0.213448	0.324321
ACAD10	<0.001	0.223514	0.173821	0.287414
ACAD8	<0.001	0.541547	0.485625	0.603907
ACADL	<0.001	0.619557	0.566089	0.678075
ACADM	<0.001	0.52366	0.475777	0.576361
ACADS	7.33E-15	0.251544	0.177654	0.356166
ACADSB	<0.001	0.500795	0.451606	0.555341
ACADVL	<0.001	0.32936	0.257212	0.421745
ACAN	<0.001	0.240534	0.177024	0.326831
ACAP1	<0.001	0.402023	0.333385	0.484791
ACAP2	<0.001	0.510998	0.457408	0.570866
ACAT1	<0.001	0.274012	0.220783	0.340075
ACAT2	<0.001	0.560574	0.489842	0.641518
ACBD3	<0.001	0.501641	0.448249	0.561394
ACBD4	<0.001	0.198806	0.14918	0.26494
ACE	0.000744	0.753003	0.638561	0.887956
ACE2	<0.001	0.677315	0.619648	0.740349
ACHE	1.40E-09	1.403109	1.25743	1.565665
ACLY	<0.001	0.403688	0.327107	0.498199
ACO1	<0.001	0.379982	0.319877	0.45138
ACO2	<0.001	0.316115	0.250165	0.39945
ACOT11	0.782032	1.031102	0.829987	1.28095
ACOT7	0.161971	1.186021	0.933795	1.506374
ACOT8	<0.001	0.392163	0.33436	0.459958
ACOT9	<0.001	0.459277	0.401638	0.525188
ACOX1	<0.001	0.196719	0.148687	0.260267
ACOX2	<0.001	0.603102	0.543795	0.668877
ACOX3	<0.001	0.362679	0.300247	0.438093
ACOXL	<0.001	0.450475	0.388269	0.522649
ACP1	<0.001	0.381994	0.323572	0.450964
ACP2	1.56E-06	0.543546	0.423837	0.697065
ACP5	7.37E-06	0.718022	0.621207	0.829924
ACP6	<0.001	0.458699	0.401559	0.52397
ACPP	2.44E-15	0.506728	0.428239	0.599602
ACSBG1	4.87E-12	0.54966	0.463837	0.651363
ACSBG2	1.84E-11	0.59582	0.512279	0.692985

(*Continued*)

Table S1: *Continued*

Gene	*P* value	HR	lower 95% CI	upper 95% CI
ACSF2	4.66E-15	0.604865	0.533379	0.685931
ACSL1	<0.001	0.536384	0.483084	0.595565
ACSL3	<0.001	0.535871	0.48637	0.59041
ACSL4	<0.001	0.426609	0.371542	0.489838
ACSL5	<0.001	0.705519	0.662069	0.75182
ACSL6	<0.001	0.211561	0.160415	0.279015
ACSM1	0.017736	1.246935	1.039006	1.496474
ACSM3	<0.001	0.578575	0.527531	0.634559
ACSM5	<0.001	0.293027	0.237357	0.361754
ACSS3	<0.001	0.508034	0.454909	0.567364
ACVR1	<0.001	0.445979	0.388717	0.511677
ACVR1B	<0.001	0.225812	0.165794	0.307557
ACVR2A	<0.001	0.444348	0.392611	0.502903
ACVR2B	<0.001	0.378184	0.312487	0.457694
ACVRL1	3.30E-05	0.644491	0.52379	0.793004
ACYP1	<0.001	0.53738	0.484092	0.596534
ADA	0.472584	1.051643	0.916636	1.206534
ADAM9	<0.001	0.518815	0.46418	0.57988
ADAMTS1	<0.001	0.544551	0.490212	0.604914
ADAMTS13	<0.001	0.203722	0.15454	0.268556
ADAMTS2	7.69E-05	0.671427	0.551116	0.818002
ADAT1	<0.001	0.409649	0.350147	0.479262
ADCY1	0.017941	1.242463	1.038025	1.487165
ADCY10	0.00045	0.735403	0.619403	0.873126
ADCY2	<0.001	0.382025	0.315021	0.463281
ADCY3	<0.001	0.38186	0.319254	0.456743
ADCY6	<0.001	0.322857	0.26458	0.39397
ADCY7	2.01E-09	0.576422	0.481445	0.690136
ADCY8	0.000762	1.194247	1.076979	1.324284
ADCY9	<0.001	0.261005	0.213129	0.319635
ADH1A	<0.001	0.383358	0.328946	0.44677
ADH1B	<0.001	0.708118	0.661851	0.75762
ADH1C	<0.001	0.715904	0.666042	0.769499
ADH5	<0.001	0.468886	0.415953	0.528556
ADH6	3.45E-08	0.643776	0.550531	0.752814
ADH7	0.028667	0.910941	0.83792	0.990324
ADI1	<0.001	0.372223	0.317861	0.435884
ADIPOQ	<0.001	0.468614	0.405332	0.541776
ADK	<0.001	0.497507	0.441077	0.561155
ADM	0.081609	0.920215	0.838005	1.010489

(*Continued*)

Table S1: *Continued*

Gene	*P* value	HR	lower 95% CI	upper 95% CI
ADM2	2.63E-12	1.706082	1.468944	1.981503
ADO	<0.001	0.399259	0.344172	0.463162
ADORA1	1.11E-16	0.322772	0.247442	0.421035
ADORA2B	7.54E-06	0.775783	0.694191	0.866964
ADORA3	1.25E-14	0.621824	0.551081	0.701647
ADPGK	<0.001	0.300683	0.240657	0.37568
ADPRH	<0.001	0.266754	0.20859	0.341136
ADPRM	<0.001	0.30839	0.252701	0.376353
ADRA1A	2.11E-07	0.51288	0.398563	0.659987
ADRA1B	0.191503	1.093623	0.95619	1.25081
ADRA1D	0.059816	1.172753	0.993433	1.384441
ADRA2A	<0.001	0.606577	0.552557	0.665879
ADRA2B	8.37E-09	0.569614	0.470353	0.689823
ADRA2C	1.11E-16	0.565821	0.494532	0.647387
ADRB1	9.99E-16	0.509503	0.432176	0.600666
ADRB2	<0.001	0.636002	0.585432	0.690942
ADRB3	0.000417	1.422619	1.16969	1.730241
ADRBK1	3.76E-06	1.515764	1.270787	1.807968
ADRBK2	<0.001	0.442052	0.386388	0.505735
ADSL	<0.001	0.426409	0.365282	0.497764
ADSS	<0.001	0.454785	0.382933	0.540119
AFP	0.847554	0.986045	0.854422	1.137945
AGA	<0.001	0.483972	0.432105	0.542064
AGAP1	<0.001	0.338368	0.285561	0.40094
AGAP2	2.85E-10	1.677002	1.428093	1.969296
AGK	<0.001	0.380198	0.321996	0.44892
AGL	<0.001	0.602088	0.557259	0.650524
AGMAT	8.52E-12	0.49835	0.408066	0.608609
AGPAT1	<0.001	0.339351	0.277696	0.414695
AGPAT2	0.000114	0.652864	0.525761	0.810694
AGPAT3	<0.001	0.374907	0.3152	0.445924
AGPAT4	<0.001	0.47219	0.402288	0.55424
AGPAT5	<0.001	0.523333	0.470655	0.581906
AGPS	<0.001	0.5104	0.459243	0.567257
AGT	2.82E-09	0.739318	0.669211	0.816769
AGTR1	<0.001	0.470351	0.412949	0.535732
AGTR2	3.14E-09	0.639157	0.551183	0.741172
AGXT	1.62E-06	1.635655	1.33773	1.999932
AHCY	9.79E-08	0.589203	0.48506	0.715707
AHCYL1	<0.001	0.302098	0.243262	0.375164

(*Continued*)

Table S1: *Continued*

Gene	*P* value	HR	lower 95% CI	upper 95% CI
AHCYL2	<0.001	0.565877	0.511401	0.626156
AHR	<0.001	0.550391	0.497271	0.609184
AHSG	0.003458	1.236278	1.072423	1.425168
AICDA	0.455658	0.955538	0.847918	1.076816
AIFM1	<0.001	0.412426	0.356379	0.477288
AK1	3.57E-10	0.595476	0.506417	0.700197
AK2	<0.001	0.355935	0.296256	0.427637
AK5	0.544471	0.954054	0.819434	1.11079
AKAP10	<0.001	0.336061	0.278587	0.405392
AKAP13	<0.001	0.377081	0.311301	0.456761
AKAP5	<0.001	0.354984	0.292125	0.431369
AKAP7	<0.001	0.323041	0.271535	0.384316
AKAP8	<0.001	0.280295	0.22927	0.342677
AKAP9	<0.001	0.417734	0.362227	0.481746
AKR1A1	<0.001	0.424634	0.363147	0.496532
AKR1B1	1.58E-12	0.671205	0.600946	0.749678
AKR1B10	0.001489	1.067748	1.025426	1.111816
AKR1C1	0.025785	1.105538	1.012201	1.207481
AKR1C3	0.000266	0.893757	0.841393	0.94938
AKR1C4	5.09E-10	0.696271	0.621164	0.78046
AKR1D1	2.24E-10	0.704969	0.63279	0.785381
AKR7A2	<0.001	0.395525	0.33115	0.472415
AKR7A3	<0.001	0.448142	0.371574	0.540487
AKT1	1.92E-10	0.458288	0.360441	0.582697
AKT2	1.46E-08	0.366014	0.258538	0.51817
AKT3	<0.001	0.383664	0.328119	0.448613
ALAD	<0.001	0.302487	0.245989	0.371961
ALAS1	<0.001	0.465932	0.409545	0.530082
ALAS2	0.00013	1.578143	1.249213	1.993683
ALB	<0.001	0.367112	0.295511	0.456062
ALDH18A1	<0.001	0.450856	0.381019	0.533494
ALDH1A1	5.38E-08	0.818298	0.761244	0.879628
ALDH1A2	2.87E-11	1.476818	1.316575	1.656563
ALDH1A3	3.44E-15	0.560592	0.485369	0.647472
ALDH1B1	1.39E-07	1.596461	1.34137	1.900063
ALDH1L1	3.26E-11	0.428406	0.3335	0.55032
ALDH2	<0.001	0.579332	0.528555	0.634987
ALDH3A1	0.233035	0.958186	0.893229	1.027866
ALDH3A2	<0.001	0.515435	0.464504	0.57195
ALDH3B1	8.28E-06	0.610497	0.491421	0.758426

(*Continued*)

Table S1: *Continued*

Gene	*P* value	HR	lower 95% CI	upper 95% CI
ALDH3B2	1.90E-05	1.265209	1.135911	1.409225
ALDH4A1	7.79E-10	1.850761	1.521024	2.251982
ALDH5A1	<0.001	0.482403	0.42534	0.54712
ALDH6A1	<0.001	0.398401	0.334787	0.474103
ALDH7A1	<0.001	0.55757	0.501494	0.619916
ALDH8A1	<0.001	0.57029	0.510013	0.63769
ALDH9A1	<0.001	0.412946	0.355098	0.480218
ALDOA	0.127049	1.210987	0.946991	1.548577
ALDOB	4.76E-05	0.590498	0.458104	0.761154
ALDOC	0.731884	1.028718	0.874891	1.209592
ALG12	0.153143	0.860435	0.700083	1.057515
ALG13	<0.001	0.367309	0.313876	0.429839
ALG3	0.003724	0.734851	0.596747	0.904916
ALG5	<0.001	0.343696	0.291022	0.405904
ALG6	<0.001	0.452921	0.401407	0.511046
ALG8	<0.001	0.434168	0.375684	0.501757
ALG9	<0.001	0.466916	0.410318	0.53132
ALK	4.65E-12	0.48136	0.391274	0.592186
ALKBH1	<0.001	0.389907	0.333608	0.455707
ALLC	1.47E-05	0.762203	0.674098	0.861824
ALOX12	1.27E-13	0.601545	0.525872	0.688108
ALOX12B	9.10E-09	1.421493	1.260808	1.602656
ALOX15	0.034362	0.907895	0.830159	0.992909
ALOX15B	1.34E-09	0.729212	0.658436	0.807597
ALOX5	<0.001	0.471434	0.405063	0.548681
ALOXE3	0.011371	0.810282	0.688474	0.953639
ALPI	4.64E-14	1.584518	1.405872	1.785864
ALPL	4.01E-06	0.712635	0.617055	0.823021
ALPP	<0.001	1.528675	1.402533	1.666162
ALPPL2	0.232997	1.113735	0.933047	1.329413
ALS2CL	<0.001	0.394408	0.329333	0.472342
AMACR	2.78E-15	0.362445	0.281768	0.46622
AMBP	3.95E-05	0.73877	0.639461	0.853503
AMD1	<0.001	0.483039	0.431235	0.541065
AMFR	0.01467	0.834283	0.721304	0.964959
AMHR2	1.70E-14	1.46118	1.326242	1.609847
AMN	0.414468	0.918052	0.747626	1.127329
AMPD1	<0.001	0.596981	0.538199	0.662183
AMPD2	<0.001	0.414154	0.351247	0.488328
AMPD3	<0.001	0.534503	0.477524	0.59828

(*Continued*)

Table S1: *Continued*

Gene	*P* value	HR	lower 95% CI	upper 95% CI
AMT	<0.001	0.349885	0.296962	0.41224
ANG	9.10E-15	0.626868	0.557038	0.705452
ANKH	<0.001	0.343157	0.281232	0.418716
ANKS1B	<0.001	0.384303	0.308041	0.479446
ANO1	6.17E-12	0.608245	0.527872	0.700856
ANO10	<0.001	0.343945	0.269522	0.438917
ANO2	0.264836	1.109311	0.924401	1.33121
ANO3	<0.001	0.54078	0.480411	0.608736
ANPEP	3.48E-05	0.663767	0.546693	0.805914
ANTXR1	3.24E-10	0.509717	0.413128	0.628887
ANXA1	6.66E-12	0.708647	0.642283	0.781867
ANXA11	<0.001	0.410124	0.351704	0.47825
ANXA13	0.11161	0.9279	0.846185	1.017506
ANXA2	0.000304	0.641149	0.503732	0.816054
ANXA2P2	1.41E-14	0.503344	0.422604	0.599509
ANXA3	<0.001	0.696366	0.653862	0.741632
ANXA4	<0.001	0.522005	0.468073	0.582151
ANXA5	<0.001	0.448542	0.384454	0.523312
ANXA6	4.15E-05	0.524394	0.385138	0.714
ANXA7	<0.001	0.335898	0.283322	0.39823
ANXA9	0.001654	1.18865	1.067327	1.323764
AOAH	1.18E-06	0.614915	0.505403	0.748157
AOC2	<0.001	0.402398	0.340862	0.475043
AOC3	<0.001	0.714779	0.664356	0.769029
AOX1	<0.001	0.456227	0.396955	0.52435
APAF1	<0.001	0.288453	0.232693	0.357574
APCS	2.14E-11	0.540695	0.451634	0.647318
APEX1	<0.001	0.328748	0.271823	0.397595
APIP	<0.001	0.405322	0.350458	0.468776
APLP1	2.55E-06	1.271773	1.150562	1.405753
APOA1	<0.001	0.311221	0.249093	0.388844
APOA4	7.49E-05	1.374836	1.174435	1.609432
APOB	8.87E-06	0.667068	0.557965	0.797505
APOBEC3B	0.000732	0.859964	0.787871	0.938654
APOBEC3C	0.025028	1.300638	1.03351	1.63681
APOBEC3F	<0.001	0.220027	0.167988	0.288186
APOBEC3G	0.000153	1.515819	1.222179	1.880008
APOC3	1.14E-09	1.701233	1.433771	2.018589
APOE	0.000175	0.767242	0.668092	0.881107
APOH	1.74E-09	0.6913	0.613017	0.779579

(*Continued*)

Table S1: *Continued*

Gene	*P* value	HR	lower 95% CI	upper 95% CI
APP	<0.001	0.337774	0.266065	0.428809
APRT	1.21E-14	0.413831	0.330732	0.517809
APTX	<0.001	0.317186	0.247173	0.407032
AQP1	3.09E-12	0.713782	0.649247	0.784731
AQP2	0.029499	1.294564	1.026046	1.633353
AQP3	0.00123	0.842472	0.759293	0.934762
AQP4	<0.001	0.747224	0.703546	0.793613
AR	<0.001	0.467158	0.409222	0.533296
ARAF	<0.001	0.221569	0.169232	0.290092
ARCN1	<0.001	0.39465	0.33888	0.459598
ARF3	3.77E-15	0.346568	0.266111	0.451351
ARF4	<0.001	0.379001	0.321945	0.44617
ARF5	4.59E-13	2.059577	1.693552	2.50471
ARF6	<0.001	0.327004	0.259728	0.411706
ARFGAP1	1.11E-16	0.322726	0.247337	0.421094
ARFGAP2	9.26E-08	0.495312	0.382738	0.640996
ARFGAP3	<0.001	0.460099	0.407858	0.519031
ARFGEF1	<0.001	0.51329	0.459361	0.573551
ARFGEF2	<0.001	0.326814	0.268605	0.397638
ARFIP1	<0.001	0.518876	0.467096	0.576396
ARFIP2	0.853589	0.976073	0.754704	1.262374
ARG1	0.737028	1.036386	0.841248	1.276789
ARG2	<0.001	0.535225	0.468882	0.610954
ARHGAP29	<0.001	0.52351	0.468303	0.585224
ARHGAP32	<0.001	0.530294	0.472297	0.595414
ARHGDIA	1.27E-13	1.886672	1.595033	2.231635
ARHGDIB	<0.001	0.406882	0.344041	0.481201
ARHGDIG	1.00E-07	0.5757	0.469843	0.705407
ARHGEF10L	<0.001	0.458691	0.385488	0.545795
ARHGEF11	3.85E-13	0.338488	0.252665	0.453463
ARHGEF12	<0.001	0.142352	0.103326	0.196117
ARHGEF17	<0.001	0.297019	0.235257	0.374996
ARHGEF18	<0.001	0.446516	0.382307	0.521508
ARHGEF2	<0.001	0.258254	0.199579	0.33418
ARHGEF3	<0.001	0.482292	0.427101	0.544615
ARHGEF4	0.000567	1.282599	1.113352	1.477575
ARHGEF6	<0.001	0.520433	0.46991	0.576388
ARHGEF7	<0.001	0.376175	0.318849	0.443808
ARHGEF9	<0.001	0.385707	0.334179	0.44518
ARL2BP	8.35E-11	0.461943	0.365897	0.583199

(*Continued*)

Table S1: *Continued*

Gene	*P* value	HR	lower 95% CI	upper 95% CI
ARL4C	7.53E-12	0.544518	0.45756	0.648003
ARSA	0.101502	0.822645	0.651232	1.039177
ARSB	<0.001	0.253196	0.193289	0.33167
ARSD	<0.001	0.495177	0.425687	0.576011
ARSE	1.61E-10	0.763981	0.70347	0.829696
ARSF	3.87E-06	1.383268	1.205332	1.587471
ARSJ	<0.001	0.622025	0.569812	0.679023
ART1	0.351377	1.116976	0.885117	1.40957
ART3	2.29E-14	0.644335	0.57557	0.721315
ART4	<0.001	0.47585	0.414623	0.546119
ASAH1	<0.001	0.411256	0.358238	0.47212
ASAP1	<0.001	0.46547	0.39417	0.549669
ASAP2	<0.001	0.630307	0.581902	0.682738
ASCC3	<0.001	0.504229	0.450054	0.564925
ASGR1	2.26E-10	0.547764	0.454793	0.65974
ASGR2	0.840774	0.985799	0.857412	1.133411
ASH1L	<0.001	0.44545	0.390776	0.507774
ASIP	0.27339	0.930727	0.818523	1.058311
ASL	<0.001	0.565494	0.500593	0.63881
ASMT	0.000412	1.338849	1.138713	1.57416
ASMTL	<0.001	0.398812	0.333966	0.476249
ASNA1	<0.001	0.421195	0.356796	0.497218
ASNS	0.002646	0.717054	0.577264	0.890696
ASPA	2.51E-11	0.5986	0.514842	0.695985
ASPH	<0.001	0.529131	0.473831	0.590885
ASRGL1	<0.001	0.616759	0.553966	0.68667
ASS1	3.21E-08	0.754293	0.682552	0.833574
ATAD2	<0.001	0.652873	0.59237	0.719557
ATAT1	<0.001	0.319486	0.260423	0.391944
ATF1	<0.001	0.484712	0.432963	0.542647
ATF2	<0.001	0.312512	0.256661	0.380516
ATF3	4.60E-11	0.57832	0.491317	0.680729
ATF4	<0.001	0.318105	0.259074	0.390587
ATF5	5.94E-11	1.71028	1.456379	2.008446
ATF7	<0.001	0.208314	0.160395	0.270549
ATIC	<0.001	0.410263	0.346164	0.48623
ATM	<0.001	0.294925	0.243079	0.35783
ATOX1	0.012302	1.410987	1.077596	1.847524
ATP10A	1.71E-05	0.575175	0.446985	0.740127
ATP10B	1.92E-10	0.69835	0.625282	0.779956

(*Continued*)

Table S1: *Continued*

Gene	*P* value	HR	lower 95% CI	upper 95% CI
ATP10D	<0.001	0.573472	0.523708	0.627966
ATP11A	<0.001	0.479393	0.410235	0.560211
ATP11B	<0.001	0.535827	0.478345	0.600217
ATP12A	2.97E-05	0.774967	0.687562	0.873483
ATP13A1	<0.001	0.357977	0.288819	0.443696
ATP13A2	1.00E-12	0.414112	0.324993	0.527668
ATP13A3	<0.001	0.54615	0.488533	0.610564
ATP1A1	<0.001	0.462422	0.390149	0.548082
ATP1A2	<0.001	0.511823	0.44319	0.591085
ATP1B1	<0.001	0.528393	0.475184	0.58756
ATP1B2	<0.001	0.541982	0.470452	0.624389
ATP1B3	0.631714	0.958531	0.806106	1.139777
ATP1B4	0.684731	0.962674	0.801176	1.156725
ATP2A1	0.012598	1.267237	1.052096	1.526372
ATP2A2	<0.001	0.281703	0.211637	0.374965
ATP2A3	1.76E-08	1.493047	1.29874	1.716425
ATP2B1	<0.001	0.552103	0.500159	0.609442
ATP2B2	8.91E-09	1.855609	1.503045	2.290871
ATP2B3	1.85E-07	0.544518	0.433293	0.684295
ATP2B4	<0.001	0.402822	0.341703	0.474873
ATP2C1	<0.001	0.491205	0.439076	0.549522
ATP2C2	<0.001	0.447663	0.38216	0.524394
ATP4A	3.65E-05	1.229636	1.114691	1.356434
ATP4B	4.59E-11	1.596484	1.388937	1.835044
ATP5A1	1.39E-10	0.465516	0.368561	0.587977
ATP5B	<0.001	0.416942	0.347743	0.499912
ATP5C1	<0.001	0.426863	0.37057	0.491708
ATP5D	0.674075	0.93321	0.676195	1.287913
ATP5E	<0.001	0.276132	0.224481	0.339666
ATP5F1	<0.001	0.432526	0.380029	0.492275
ATP5G1	0.000324	0.635664	0.496542	0.813765
ATP5G3	<0.001	0.321288	0.257249	0.401268
ATP5H	<0.001	0.200408	0.149414	0.268805
ATP5I	0.000347	0.54684	0.392854	0.761183
ATP5J	<0.001	0.305965	0.25157	0.372121
ATP5O	<0.001	0.307912	0.253146	0.374525
ATP6AP1	<0.001	0.321601	0.247426	0.418011
ATP6V0A2	<0.001	0.277153	0.226004	0.339879
ATP6V0A4	2.79E-08	0.800218	0.739692	0.865696
ATP6V0C	<0.001	2.452403	2.027962	2.965678

(*Continued*)

Table S1: *Continued*

Gene	*P* value	HR	lower 95% CI	upper 95% CI
ATP6V0D1	0.023613	0.733705	0.561139	0.959341
ATP6V0E1	<0.001	0.330452	0.275225	0.396761
ATP6V0E2	<0.001	0.487861	0.422457	0.56339
ATP6V1A	3.77E-15	0.51493	0.436418	0.607566
ATP6V1B1	0.925828	1.007869	0.85455	1.188695
ATP6V1B2	<0.001	0.44252	0.378144	0.517855
ATP6V1C1	<0.001	0.466868	0.410536	0.530929
ATP6V1D	<0.001	0.393923	0.338124	0.458931
ATP6V1E1	<0.001	0.369028	0.31344	0.434474
ATP6V1G1	<0.001	0.329932	0.27651	0.393675
ATP6V1G2	<0.001	0.266203	0.209464	0.338311
ATP6V1H	<0.001	0.431677	0.358702	0.519497
ATP7A	<0.001	0.475015	0.422076	0.534594
ATP7B	<0.001	0.584824	0.525756	0.650527
ATP8A1	<0.001	0.468224	0.415551	0.527574
ATP8A2	2.84E-08	0.651822	0.5604	0.758157
ATP8B1	<0.001	0.534226	0.481205	0.59309
ATP8B2	1.72E-09	0.489926	0.388396	0.617997
ATP8B3	2.91E-07	0.609826	0.504816	0.736679
ATP8B4	<0.001	0.522527	0.468063	0.583329
ATP9A	<0.001	0.499745	0.43931	0.568495
ATP9B	2.89E-15	0.331046	0.251604	0.435572
ATR	<0.001	0.523186	0.472363	0.579478
ATRX	<0.001	0.550129	0.501513	0.603457
AUH	<0.001	0.459944	0.407795	0.518761
AURKA	0.000491	0.792627	0.695529	0.90328
AURKB	1.96E-08	1.44263	1.269424	1.639468
AURKC	0.229956	1.087722	0.948193	1.247783
AVPI1	7.71E-12	0.570261	0.485532	0.669776
AVPR1A	1.11E-16	0.359946	0.283022	0.457776
AVPR1B	7.04E-13	1.553395	1.377388	1.751893
AVPR2	1.05E-12	1.508937	1.347456	1.689772
AXL	<0.001	0.402234	0.331872	0.487515
AZGP1	1.18E-13	0.829619	0.789676	0.871581
AZIN1	<0.001	0.482406	0.430262	0.540869
AZU1	0.019927	0.863713	0.763469	0.977118
B2M	<0.001	0.42499	0.356849	0.506144
B3GALNT1	<0.001	0.62257	0.568887	0.681319
B3GALT1	2.18E-07	0.59768	0.491971	0.726102
B3GALT2	<0.001	0.557506	0.499554	0.622181

(*Continued*)

Table S1: *Continued*

Gene	*P* value	HR	lower 95% CI	upper 95% CI
B3GALT4	<0.001	0.310728	0.246647	0.391457
B3GALT5	0.053595	1.099766	0.99853	1.211265
B3GAT1	0.470943	0.972747	0.902354	1.048632
B3GAT3	1.26E-11	0.301009	0.212665	0.426051
B3GNT1	<0.001	0.276992	0.22541	0.340376
B4GALNT1	1.11E-15	0.403583	0.323292	0.503816
B4GALT1	0.227368	1.22643	0.880484	1.708299
B4GALT2	0.894971	0.978095	0.704005	1.358897
B4GALT3	<0.001	0.36274	0.290016	0.4537
B4GALT4	<0.001	0.554937	0.486614	0.632853
B4GALT5	<0.001	0.488249	0.424918	0.561019
B4GALT6	<0.001	0.448656	0.37218	0.540847
B4GALT7	<0.001	0.350082	0.281224	0.435801
BAAT	1.97E-06	0.753185	0.670166	0.846488
BAIAP2	5.79E-10	0.530861	0.434497	0.648597
BATF	2.44E-15	0.539398	0.462969	0.628445
BBOX1	9.94E-12	0.716074	0.650426	0.788349
BCAN	0.23036	1.156742	0.911808	1.467471
BCAT1	3.23E-12	0.716071	0.65186	0.786606
BCAT2	8.11E-08	0.568991	0.463058	0.699159
BCHE	<0.001	0.6708	0.629385	0.714941
BCKDHA	<0.001	0.326382	0.266198	0.400173
BCKDHB	<0.001	0.341273	0.288591	0.403571
BCKDK	<0.001	0.354534	0.289648	0.433956
BCL2	<0.001	0.258816	0.205456	0.326034
BCMO1	<0.001	0.576503	0.513726	0.646951
BCR	<0.001	0.346613	0.282073	0.425922
BCS1L	<0.001	0.337761	0.284046	0.401635
BDH1	0.004023	1.245125	1.072346	1.445743
BDH2	<0.001	0.481315	0.430762	0.5378
BDKRB1	3.06E-10	0.673079	0.59503	0.761365
BDKRB2	0.000177	0.756783	0.654204	0.875447
BEST1	<0.001	0.338472	0.265121	0.432118
BEST2	0.040384	0.862002	0.747898	0.993513
BHLHE40	0.000103	0.748994	0.647342	0.866607
BHMT	0.131671	1.079025	0.977433	1.191176
BHMT2	1.82E-09	0.70017	0.623372	0.78643
BLK	0.000181	0.758065	0.655764	0.876325
BLM	<0.001	0.573654	0.508728	0.646867
BLVRA	<0.001	0.330762	0.272973	0.400784

(*Continued*)

Table S1: *Continued*

Gene	*P* value	HR	lower 95% CI	upper 95% CI
BLVRB	1.04E-08	0.630018	0.53783	0.738007
BLZF1	<0.001	0.464195	0.410926	0.524369
BMP1	0.001479	1.477697	1.161507	1.879963
BMP2	<0.001	0.729438	0.678124	0.784635
BMP2K	<0.001	0.442467	0.386421	0.506641
BMP7	0.000319	0.727907	0.612305	0.865336
BMP8A	0.000173	1.326968	1.144854	1.53805
BMP8B	<0.001	0.28294	0.227102	0.352507
BMPR1A	<0.001	0.421215	0.368295	0.481738
BMPR1B	6.88E-15	0.650566	0.58384	0.724917
BMPR2	<0.001	0.451812	0.397697	0.513291
BMX	2.49E-13	0.686013	0.620161	0.758857
BNIP3	0.195132	0.910993	0.791167	1.048969
BPGM	<0.001	0.491549	0.433919	0.556833
BPNT1	<0.001	0.353764	0.290805	0.430354
BRIP1	3.54E-06	0.759195	0.675734	0.852964
BRS3	0.000362	0.682286	0.552981	0.841826
BRSK2	6.27E-06	1.389215	1.204547	1.602195
BSPRY	<0.001	0.628825	0.569362	0.6945
BST1	<0.001	0.481573	0.418317	0.554395
BTD	<0.001	0.281046	0.22732	0.347469
BTK	<0.001	0.43719	0.378782	0.504604
BUB1	0.07445	0.848574	0.708498	1.016344
BUB1B	2.29E-05	0.818467	0.745995	0.89798
C1GALT1	<0.001	0.566584	0.517032	0.620884
C1QA	0.133552	0.907265	0.798945	1.030271
C1QB	0.181156	1.094859	0.958672	1.250391
C1R	2.48E-07	0.654118	0.556706	0.768576
C1S	1.48E-08	0.693074	0.6105	0.786816
C2orf83	0.094029	1.123556	0.980337	1.287698
C5AR1	3.05E-09	0.679031	0.597472	0.771724
C9orf156	<0.001	0.274233	0.218837	0.343652
CA1	<0.001	0.212398	0.160044	0.281878
CA10	<0.001	0.613004	0.548984	0.68449
CA12	0.589723	1.027875	0.930124	1.135899
CA14	0.000889	1.228963	1.088272	1.387842
CA2	1.11E-16	0.70871	0.653349	0.768761
CA3	<0.001	0.615668	0.559008	0.678071
CA4	5.03E-09	0.687201	0.60599	0.779295
CA5A	2.97E-10	1.431569	1.280388	1.600601

(*Continued*)

Table S1: *Continued*

Gene	*P* value	HR	lower 95% CI	upper 95% CI
CA5B	<0.001	0.395802	0.325102	0.481877
CA6	0.031087	1.180705	1.015216	1.373171
CA7	0.000121	1.298106	1.13644	1.482771
CA8	2.57E-09	0.613341	0.522216	0.720367
CA9	0.197186	0.938034	0.851131	1.033811
CABIN1	<0.001	0.239605	0.178867	0.320967
CABYR	0.255756	0.938663	0.84159	1.046934
CACNA1B	2.55E-08	1.415535	1.252599	1.599666
CACNA1C	<0.001	0.215916	0.168205	0.277162
CACNA1D	<0.001	0.45946	0.399076	0.528981
CACNA1E	0.000124	0.597761	0.459659	0.777354
CACNA1F	3.33E-15	1.607285	1.428267	1.80874
CACNA1G	0.137382	0.82942	0.648059	1.061537
CACNA1H	3.61E-11	1.644092	1.419021	1.904861
CACNA1I	0.997127	0.999591	0.800127	1.24878
CACNA1S	0.610157	1.034421	0.908234	1.178141
CACNA2D1	<0.001	0.512613	0.444096	0.591702
CACNA2D2	<0.001	0.721588	0.673166	0.773492
CACNA2D3	<0.001	0.541797	0.486006	0.603993
CACNB1	<0.001	0.251615	0.196533	0.322135
CACNB2	<0.001	0.285831	0.225501	0.362301
CACNB3	<0.001	0.388128	0.329904	0.456628
CACNB4	<0.001	0.496186	0.425027	0.579258
CACNG1	0.013014	1.179522	1.035413	1.343688
CACNG2	0.843567	0.981465	0.815026	1.181892
CACNG3	1.33E-15	1.649376	1.458841	1.864797
CACNG4	0.27868	1.130801	0.905307	1.412462
CACNG5	2.21E-09	0.434116	0.330264	0.570623
CACYBP	<0.001	0.400538	0.343529	0.467008
CAD	<0.001	0.336019	0.266856	0.423107
CADM3	0.918952	1.010736	0.822819	1.24157
CADM4	0.013245	1.269929	1.051154	1.534237
CADPS	1.20E-10	0.458062	0.361178	0.580933
CADPS2	<0.001	0.616009	0.571529	0.66395
CALB1	0.738418	0.989715	0.931449	1.051626
CALB2	5.96E-08	0.622877	0.524875	0.739177
CALCA	0.008952	1.065814	1.016073	1.11799
CALCOCO1	<0.001	0.363377	0.303615	0.434903
CALD1	<0.001	0.461941	0.401084	0.532032
CALM1	<0.001	0.445011	0.392608	0.504408

(*Continued*)

Table S1: *Continued*

Gene	*P* value	HR	lower 95% CI	upper 95% CI
CALML3	0.000925	1.18757	1.072715	1.314722
CALML5	0.90376	0.994777	0.913822	1.082904
CALR	5.01E-05	1.899633	1.393091	2.590358
CALU	9.50E-05	0.655551	0.530291	0.810399
CALY	0.531814	1.040067	0.919557	1.176369
CAMK1	<0.001	0.396065	0.325232	0.482324
CAMK1D	<0.001	0.599754	0.536012	0.671077
CAMK1G	5.16E-06	1.515783	1.267555	1.812622
CAMK2B	0.060443	1.17354	0.993	1.386905
CAMK4	<0.001	0.405096	0.348467	0.470929
CAMKK2	<0.001	0.238112	0.178541	0.31756
CAMKMT	9.63E-14	0.520148	0.437928	0.617803
CAMKV	1.26E-05	0.702251	0.599247	0.822961
CAMLG	<0.001	0.440929	0.384325	0.505871
CANT1	<0.001	0.413948	0.350089	0.489455
CANX	<0.001	0.401408	0.345548	0.466297
CAPG	7.15E-13	0.59341	0.514585	0.684311
CAPN1	<0.001	0.337614	0.270489	0.421396
CAPN10	0.008289	0.798405	0.675515	0.943651
CAPN11	0.170173	1.105834	0.957769	1.27679
CAPN2	<0.001	0.461178	0.404979	0.525176
CAPN3	<0.001	0.477947	0.420075	0.543791
CAPN7	<0.001	0.5052	0.454545	0.561501
CAPN9	2.11E-15	0.630464	0.562593	0.706523
CAPNS1	0.34325	0.894105	0.709349	1.126981
CARKD	<0.001	0.4618	0.404559	0.52714
CARM1	5.11E-10	1.88104	1.541257	2.295731
CARS	6.89E-06	0.565927	0.441574	0.7253
CARS2	<0.001	0.19182	0.142097	0.258942
CARTPT	1.55E-05	1.698916	1.335875	2.160617
CASK	<0.001	0.435235	0.377469	0.501841
CASP1	<0.001	0.561631	0.510046	0.618433
CASQ1	<0.001	0.542058	0.474643	0.61905
CASQ2	6.44E-15	0.665722	0.600989	0.737427
CASR	0.126354	0.852593	0.694931	1.046025
CAST	<0.001	0.463239	0.409089	0.524558
CAT	<0.001	0.382465	0.329254	0.444276
CBL	<0.001	0.349545	0.288393	0.423663
CBLB	<0.001	0.400777	0.339438	0.473201
CBLC	4.44E-16	1.566375	1.405979	1.74507

(*Continued*)

Table S1: *Continued*

Gene	*P* value	HR	lower 95% CI	upper 95% CI
CBR1	0.085289	1.107642	0.985891	1.244428
CBR3	4.80E-05	0.764365	0.671489	0.870087
CBS	1.29E-08	1.474352	1.289742	1.685387
CC2D1A	0.094007	0.782072	0.586549	1.042772
CCBL1	1.67E-15	0.357204	0.277265	0.46019
CCBL2	<0.001	0.504766	0.453623	0.561676
CCKAR	0.008618	1.258868	1.060186	1.494784
CCKBR	4.44E-16	0.415404	0.33615	0.513344
CCL16	1.71E-06	0.714632	0.622744	0.820078
CCL2	0.110093	1.097296	0.979173	1.229669
CCNB1IP1	<0.001	0.388947	0.334615	0.4521
CCND1	3.69E-12	0.731299	0.669517	0.798783
CCPG1	<0.001	0.567909	0.520934	0.619119
CCR1	1.16E-12	0.626193	0.550388	0.712439
CCR10	3.87E-10	0.656425	0.575362	0.74891
CCR2	<0.001	0.466632	0.410524	0.530408
CCR3	0.056256	0.883412	0.777841	1.003312
CCR4	0.011494	1.167299	1.035336	1.316082
CCR5	<0.001	0.534614	0.48349	0.591143
CCR6	<0.001	0.48569	0.425118	0.554893
CCR9	<0.001	0.472486	0.414881	0.53809
CCS	1.39E-13	0.357801	0.272501	0.469802
CD101	<0.001	0.38606	0.31252	0.476905
CD163	4.33E-15	0.628603	0.559771	0.705899
CD1A	<0.001	0.690349	0.633754	0.751997
CD1B	<0.001	0.554827	0.496428	0.620096
CD1C	<0.001	0.568491	0.511246	0.632144
CD1D	<0.001	0.546388	0.486195	0.614033
CD1E	3.97E-14	0.642655	0.573069	0.720691
CD207	9.08E-12	0.73854	0.676938	0.805748
CD209	1.55E-15	0.475311	0.395868	0.570697
CD320	5.88E-07	0.546436	0.431077	0.692665
CD36	<0.001	0.541758	0.484653	0.605591
CD38	1.73E-09	0.719041	0.645838	0.80054
CD40	<0.001	0.400366	0.324349	0.494199
CD40LG	1.02E-08	0.573706	0.474369	0.693845
CD44	<0.001	0.444914	0.37909	0.522168
CD97	1.55E-15	0.595787	0.524539	0.676714
CDA	1.39E-11	1.250589	1.172069	1.33437
CDC14A	<0.001	0.245221	0.194131	0.309758

(*Continued*)

Table S1: *Continued*

Gene	*P* value	HR	lower 95% CI	upper 95% CI
CDC14B	<0.001	0.309468	0.252775	0.378877
CDC25A	2.45E-06	1.381464	1.207739	1.580177
CDC25B	4.61E-09	0.603276	0.509466	0.714361
CDC25C	1.39E-08	1.526168	1.318825	1.766109
CDC34	2.41E-07	1.708061	1.394022	2.092845
CDC37	0.054703	0.766432	0.584276	1.005378
CDC42	<0.001	0.359516	0.304605	0.424326
CDC42BPA	<0.001	0.489361	0.433698	0.552168
CDC42BPB	<0.001	0.37635	0.317486	0.446128
CDC42EP1	0.006609	1.203118	1.052818	1.374876
CDC42EP2	1.24E-07	1.476848	1.278095	1.706508
CDC42EP3	<0.001	0.505671	0.447672	0.571183
CDC42EP4	7.34E-09	0.428971	0.321995	0.571488
CDC42SE1	<0.001	0.393339	0.330676	0.467877
CDC7	<0.001	0.636306	0.587998	0.688582
CDH1	<0.001	0.604594	0.541502	0.675037
CDH10	<0.001	0.563061	0.511579	0.619723
CDH11	<0.001	0.552011	0.496517	0.613708
CDH12	0.243492	0.944851	0.858937	1.039358
CDH13	<0.001	0.53456	0.474236	0.602558
CDH15	9.73E-06	1.392072	1.20227	1.611838
CDH16	5.00E-15	1.394164	1.282855	1.515131
CDH17	0.050568	0.926284	0.857839	1.000189
CDH18	0.020459	1.145369	1.021177	1.284665
CDH19	<0.001	0.412597	0.340857	0.499436
CDH2	3.69E-05	0.744475	0.647109	0.856491
CDH20	5.89E-07	1.335343	1.192092	1.495808
CDH22	3.96E-08	1.658518	1.384568	1.98667
CDH3	0.554748	0.972261	0.885613	1.067387
CDH4	0.849651	1.021367	0.820819	1.270915
CDH5	<0.001	0.50286	0.449493	0.562564
CDH6	<0.001	0.39346	0.326078	0.474767
CDH7	3.47E-05	1.400343	1.19401	1.642331
CDH8	<0.001	0.332437	0.259178	0.426403
CDH9	0.278938	0.91974	0.790479	1.070138
CDHR1	4.97E-05	1.294093	1.142541	1.465749
CDHR5	9.88E-15	1.432404	1.307814	1.568862
CDIPT	<0.001	0.43821	0.378375	0.507508
CDK1	0.000312	0.817577	0.732787	0.912177
CDK10	<0.001	0.279129	0.225545	0.345443

(*Continued*)

Table S1: *Continued*

Gene	*P* value	HR	lower 95% CI	upper 95% CI
CDK12	<0.001	0.250015	0.201579	0.310089
CDK13	<0.001	0.201554	0.156655	0.25932
CDK17	<0.001	0.485331	0.431102	0.546382
CDK18	0.000113	0.669018	0.545519	0.820477
CDK19	<0.001	0.315207	0.248952	0.399094
CDK2	4.70E-07	1.583231	1.324085	1.893097
CDK20	<0.001	0.226893	0.180293	0.285537
CDK4	0.011844	0.751847	0.602092	0.938849
CDK5	2.12E-12	0.461098	0.371544	0.572237
CDK5RAP1	<0.001	0.363146	0.302413	0.436075
CDK6	2.22E-16	0.524235	0.449424	0.6115
CDK7	<0.001	0.50831	0.453883	0.569263
CDK8	<0.001	0.470805	0.418174	0.530059
CDK9	0.494859	0.870857	0.585484	1.295324
CDKAL1	0.177265	0.899164	0.770518	1.049289
CDKL1	<0.001	0.362791	0.298611	0.440766
CDKL2	<0.001	0.578214	0.524329	0.637637
CDKL3	<0.001	0.245464	0.193606	0.311212
CDKL5	0.879672	0.98706	0.833896	1.168356
CDKN3	0.191482	1.060402	0.971074	1.157947
CDO1	<0.001	0.43164	0.375127	0.496666
CDS1	<0.001	0.415378	0.362124	0.476464
CDS2	<0.001	0.259915	0.20889	0.323405
CDY1	0.235671	0.920048	0.801676	1.0559
CDYL	<0.001	0.401263	0.342588	0.469987
CEACAM3	3.00E-06	0.583596	0.46556	0.731558
CECR1	4.00E-14	0.655775	0.587834	0.731569
CEL	0.000555	1.113543	1.047598	1.183639
CELF2	<0.001	0.530315	0.473585	0.59384
CEPT1	<0.001	0.465052	0.403311	0.536243
CERK	<0.001	0.378537	0.328747	0.435868
CERS2	<0.001	0.460281	0.39689	0.533797
CERS4	0.121883	0.924101	0.836153	1.0213
CERS6	<0.001	0.504687	0.44629	0.570725
CES2	<0.001	0.310795	0.247378	0.390468
CES3	0.00109	0.769783	0.657932	0.900649
CETN1	0.000265	0.747677	0.639525	0.874118
CETN2	<0.001	0.47124	0.418622	0.530472
CETP	0.087722	1.078716	0.988861	1.176735
CFTR	<0.001	0.504513	0.432769	0.588151

(*Continued*)

Table S1: *Continued*

Gene	*P* value	HR	lower 95% CI	upper 95% CI
CH25H	<0.001	0.740103	0.690876	0.792838
CHAT	0.079986	1.107893	0.987832	1.242546
CHD1	<0.001	0.570624	0.523029	0.62255
CHD1L	<0.001	0.391444	0.334261	0.458409
CHD2	<0.001	0.422492	0.368326	0.484622
CHD3	<0.001	0.21362	0.163112	0.279768
CHD4	<0.001	0.502761	0.431236	0.58615
CHD5	0.819157	1.025295	0.827649	1.27014
CHD7	2.87E-05	0.622097	0.498068	0.777012
CHD8	4.15E-11	0.427218	0.331851	0.54999
CHD9	<0.001	0.549917	0.498155	0.607056
CHEK1	1.66E-12	0.636216	0.561161	0.721311
CHEK2	9.29E-13	0.56355	0.481469	0.659624
CHERP	0.985889	1.002743	0.740209	1.358392
CHFR	<0.001	0.363957	0.304066	0.435644
CHGA	1.41E-12	0.599462	0.520313	0.69065
CHI3L1	0.001062	0.826969	0.738051	0.9266
CHI3L2	7.77E-16	0.637823	0.571789	0.711482
CHIA	1.78E-08	0.76426	0.696003	0.839211
CHIT1	0.831996	1.006519	0.947876	1.068789
CHKA	<0.001	0.432799	0.372475	0.502893
CHKB	<0.001	0.354023	0.297246	0.421646
CHN1	<0.001	0.551814	0.498192	0.611208
CHN2	<0.001	0.406575	0.347596	0.475561
CHPF	2.07E-14	0.455103	0.371945	0.556853
CHPF2	<0.001	0.346601	0.27378	0.438791
CHPT1	<0.001	0.521289	0.46507	0.584305
CHRM2	9.42E-11	1.515944	1.336592	1.719362
CHRM3	4.37E-05	0.745426	0.647457	0.858219
CHRM4	2.03E-05	1.36829	1.184546	1.580535
CHRM5	4.32E-07	0.686863	0.593761	0.794563
CHRNA10	0.07857	0.807798	0.636821	1.02468
CHRNA2	0.000108	0.657333	0.531566	0.812855
CHRNA3	3.06E-07	1.450316	1.257918	1.672141
CHRNA4	2.09E-12	1.61086	1.410309	1.83993
CHRNA5	0.314148	1.047534	0.956966	1.146674
CHRNA6	<0.001	0.432211	0.368493	0.506947
CHRNA9	2.08E-06	0.784577	0.709777	0.867259
CHRNB2	0.897641	1.012874	0.833513	1.230832
CHRNB3	1.29E-14	0.425637	0.342531	0.528907

(*Continued*)

Table S1: *Continued*

Gene	*P* value	HR	lower 95% CI	upper 95% CI
CHRNB4	<0.001	0.554914	0.484775	0.635202
CHST1	7.65E-13	0.49231	0.405582	0.597583
CHST11	7.24E-13	0.601488	0.523502	0.691093
CHST12	<0.001	0.44916	0.384374	0.524865
CHST15	<0.001	0.449285	0.383774	0.525979
CHST3	0.549968	0.946199	0.789296	1.134294
CHST5	0.632071	1.050339	0.859053	1.284219
CHST7	9.30E-11	0.599326	0.513325	0.699735
CHSY1	<0.001	0.571853	0.518237	0.631015
CHUK	<0.001	0.586648	0.539775	0.637591
CILP	1.65E-07	0.809721	0.748196	0.876305
CINP	<0.001	0.26346	0.208556	0.33282
CISD1	<0.001	0.473396	0.401982	0.557496
CKB	0.000415	0.77689	0.675286	0.893782
CKM	7.88E-12	1.288932	1.198526	1.386158
CKMT2	<0.001	0.487381	0.42379	0.560513
CLC	0.000392	0.840541	0.763573	0.925268
CLCA2	0.097082	0.952024	0.898306	1.008953
CLCA4	7.47E-07	1.168973	1.098879	1.243538
CLCC1	<0.001	0.391911	0.33855	0.453683
CLCN1	0.253845	1.100455	0.933609	1.297119
CLCN2	1.22E-15	0.400061	0.3197	0.500622
CLCN3	<0.001	0.494666	0.441911	0.553721
CLCN4	<0.001	0.31369	0.254905	0.386031
CLCN5	<0.001	0.412821	0.351589	0.484717
CLCN6	<0.001	0.3243	0.27059	0.388671
CLCN7	0.867892	0.972764	0.702584	1.346842
CLCNKB	0.96006	1.003988	0.859179	1.173202
CLDN1	6.94E-08	0.800419	0.738208	0.867873
CLDN10	0.200967	0.961328	0.90494	1.02123
CLDN11	2.09E-05	0.650155	0.533201	0.792761
CLDN14	<0.001	0.325205	0.26275	0.402505
CLDN15	6.68E-13	0.410393	0.321884	0.52324
CLDN16	0.001143	1.19518	1.073437	1.330729
CLDN17	0.000289	1.263062	1.11322	1.433073
CLDN18	6.06E-08	0.825407	0.770047	0.884746
CLDN3	0.002894	0.877461	0.805144	0.956274
CLDN9	1.22E-15	0.436255	0.356014	0.534581
CLEC10A	<0.001	0.576416	0.512934	0.647754
CLEC4A	<0.001	0.574818	0.522656	0.632184

(*Continued*)

Table S1: *Continued*

Gene	*P* value	HR	lower 95% CI	upper 95% CI
CLEC4M	0.000231	0.717892	0.601783	0.856403
CLEC5A	1.79E-14	0.717961	0.659637	0.781441
CLGN	4.80E-14	0.746718	0.692107	0.805637
CLIC1	<0.001	0.398358	0.329593	0.481468
CLIC2	<0.001	0.594734	0.547344	0.646226
CLIC3	0.203907	0.924112	0.818181	1.043759
CLIC4	2.22E-16	0.477888	0.400897	0.569665
CLIC5	<0.001	0.497203	0.430763	0.57389
CLK1	<0.001	0.539637	0.492912	0.590792
CLK2	<0.001	0.290338	0.239656	0.351737
CLK4	<0.001	0.537044	0.485632	0.593899
CLNS1A	<0.001	0.356444	0.298827	0.425169
CLOCK	<0.001	0.440856	0.389747	0.498668
CLPP	0.005387	0.663965	0.497607	0.885938
CLSTN1	<0.001	0.492854	0.425901	0.570332
CLSTN2	2.22E-16	0.623331	0.556961	0.697612
CLSTN3	0.000191	1.501668	1.212872	1.859228
CLTC	<0.001	0.439878	0.379565	0.509776
CLTCL1	0.889346	0.987497	0.8271	1.178998
CMAS	<0.001	0.433489	0.377094	0.498317
CMPK1	<0.001	0.376743	0.322389	0.440262
CNGA1	<0.001	0.511223	0.452415	0.577676
CNGA3	0.008379	1.089681	1.022281	1.161526
CNGB1	3.62E-11	0.56606	0.478279	0.669951
CNGB3	<0.001	0.463358	0.388236	0.553015
CNOT4	<0.001	0.369935	0.315271	0.434077
CNOT6	<0.001	0.487599	0.435927	0.545395
CNP	<0.001	0.226339	0.168666	0.303733
CNR1	<0.001	0.395301	0.323079	0.483669
COASY	<0.001	0.39986	0.338604	0.4722
COL13A1	<0.001	0.356015	0.293258	0.432201
COL1A1	0.168564	1.136521	0.947242	1.363622
COL1A2	6.12E-10	0.677898	0.599354	0.766736
COL4A3BP	<0.001	0.545901	0.497923	0.598501
COLEC12	<0.001	0.65446	0.60497	0.707998
COMP	3.46E-08	0.819075	0.763007	0.879263
COMT	9.82E-12	0.354795	0.263294	0.478096
COPA	<0.001	0.334481	0.278038	0.402382
COPB1	<0.001	0.439738	0.387705	0.498753
COPB2	<0.001	0.364593	0.301038	0.441566

(*Continued*)

Table S1: *Continued*

Gene	*P* value	HR	lower 95% CI	upper 95% CI
COPE	1.48E-06	1.784468	1.409599	2.259028
COPZ1	<0.001	0.3945	0.330516	0.470872
COQ2	<0.001	0.355959	0.298573	0.424374
COQ3	<0.001	0.498094	0.435704	0.569419
COQ6	<0.001	0.28069	0.229637	0.343095
COQ7	<0.001	0.331367	0.275831	0.398085
CORT	<0.001	0.313435	0.258984	0.379333
COTL1	<0.001	0.505803	0.43141	0.593023
COX10	<0.001	0.264117	0.209647	0.332739
COX11	<0.001	0.442846	0.390722	0.501924
COX15	<0.001	0.356747	0.300976	0.422852
COX4I1	<0.001	0.197461	0.145936	0.267177
COX5A	<0.001	0.364677	0.299716	0.443718
COX5B	0.279334	1.197515	0.863886	1.65999
COX6A1	<0.001	3.192783	2.557938	3.985186
COX6A2	<0.001	0.425937	0.364505	0.497723
COX6B1	0.005049	0.6787	0.517629	0.889892
COX6C	1.48E-09	0.584792	0.491446	0.695869
COX7A1	0.001962	0.758934	0.63733	0.90374
COX7A2	<0.001	0.308055	0.251007	0.378068
COX7A2L	<0.001	0.373704	0.313623	0.445294
COX7B	<0.001	0.367651	0.31625	0.427405
COX7C	<0.001	0.304732	0.236011	0.393463
COX8A	2.72E-08	1.907736	1.519191	2.395656
CP	1.00E-11	0.826806	0.782743	0.873348
CPA3	<0.001	0.708634	0.666236	0.75373
CPB1	0.828842	1.012001	0.908259	1.127593
CPB2	<0.001	0.734787	0.685167	0.788002
CPD	<0.001	0.544555	0.486858	0.60909
CPM	<0.001	0.632979	0.575364	0.696363
CPN1	1.42E-11	0.569609	0.483797	0.670641
CPN2	4.46E-07	0.714352	0.62689	0.814016
CPNE1	2.89E-15	0.574692	0.500844	0.659429
CPNE3	<0.001	0.534238	0.480565	0.593905
CPNE6	4.64E-14	1.57565	1.40005	1.773275
CPNE7	7.22E-15	0.404747	0.322279	0.508318
CPOX	<0.001	0.517446	0.460633	0.581266
CPS1	6.76E-05	0.83599	0.765477	0.912998
CPT1A	<0.001	0.406807	0.334481	0.494771
CPT2	<0.001	0.358154	0.30077	0.426486

(*Continued*)

Table S1: *Continued*

Gene	*P* value	HR	lower 95% CI	upper 95% CI
CRABP1	0.011605	1.199176	1.041421	1.380827
CRABP2	0.062445	0.930342	0.862294	1.00376
CRAT	0.000463	0.61748	0.471436	0.808765
CREB1	<0.001	0.424187	0.371771	0.483993
CREB3	<0.001	0.365751	0.295892	0.452104
CREB3L1	0.000764	0.721403	0.596463	0.872514
CREB3L2	<0.001	0.29679	0.239627	0.367591
CREBBP	<0.001	0.162739	0.122034	0.217021
CREM	<0.001	0.348934	0.292301	0.416539
CRISP2	2.16E-10	0.777718	0.719649	0.840473
CRNN	1.82E-05	1.247486	1.127509	1.380229
CROT	<0.001	0.471669	0.415081	0.535973
CRP	1.23E-08	1.562446	1.340035	1.821772
CRTC1	5.41E-09	1.525877	1.323936	1.758621
CRTC3	<0.001	0.368914	0.311229	0.437292
CRY1	<0.001	0.522924	0.472969	0.578155
CRY2	<0.001	0.266685	0.216154	0.329029
CRYL1	<0.001	0.359488	0.297548	0.434322
CRYM	<0.001	0.613675	0.555746	0.677644
CRYZ	<0.001	0.615759	0.569753	0.66548
CS	<0.001	0.367143	0.295273	0.456508
CSAD	3.22E-14	0.347408	0.264396	0.456483
CSE1L	<0.001	0.531106	0.476909	0.591462
CSF1R	<0.001	0.520072	0.456381	0.592651
CSF2RB	<0.001	0.586081	0.533668	0.643641
CSGALNACT1	<0.001	0.554769	0.503957	0.610704
CSGALNACT2	<0.001	0.512738	0.460905	0.5704
CSN1S1	9.70E-06	1.175092	1.094021	1.262171
CSNK1A1	<0.001	0.388442	0.334351	0.451285
CSNK1D	<0.001	0.263037	0.209202	0.330726
CSNK1E	0.00106	0.630905	0.478869	0.83121
CSNK1G2	0.001143	1.647181	1.219382	2.225066
CSNK1G3	<0.001	0.38249	0.320713	0.456167
CSNK2A1	<0.001	0.397348	0.325943	0.484396
CSNK2A2	<0.001	0.371557	0.314905	0.438401
CSNK2B	<0.001	0.342295	0.267883	0.437378
CSPG4	5.61E-05	0.732178	0.629143	0.852086
CTBP1	1.11E-16	0.286076	0.21262	0.384909
CTBS	<0.001	0.487301	0.435155	0.545697
CTDNEP1	6.54E-07	0.364713	0.245109	0.542678

(*Continued*)

Table S1: *Continued*

Gene	*P* value	HR	lower 95% CI	upper 95% CI
CTDP1	4.13E-14	0.329527	0.247086	0.439476
CTDSP1	<0.001	0.220993	0.171821	0.284238
CTDSP2	<0.001	0.380198	0.321118	0.450146
CTDSPL	<0.001	0.242547	0.187691	0.313436
CTGF	<0.001	0.593433	0.535079	0.658152
CTH	<0.001	0.480133	0.420681	0.547987
CTNNAL1	<0.001	0.563768	0.513685	0.618733
CTNNB1	2.63E-08	0.571554	0.469335	0.696036
CTNS	<0.001	0.303268	0.242641	0.379043
CTPS1	<0.001	0.565303	0.504352	0.633621
CTPS2	<0.001	0.493803	0.439813	0.55442
CTSC	<0.001	0.54603	0.481724	0.618921
CTSD	9.83E-11	0.625515	0.542635	0.721053
CUBN	<0.001	0.45059	0.390722	0.519632
CUL5	<0.001	0.441452	0.388403	0.501747
CUTC	<0.001	0.423941	0.371322	0.484017
CXCL11	<0.001	0.758248	0.710761	0.808908
CXCL13	0.003956	0.916755	0.864132	0.972583
CXCR1	0.006094	1.186244	1.049948	1.340233
CXCR2	8.14E-11	0.653157	0.574411	0.7427
CXCR4	<0.001	0.537156	0.470339	0.613465
CYB561	9.57E-09	0.466529	0.359565	0.605313
CYB561D2	0.001409	0.672984	0.527746	0.858193
CYB5A	<0.001	0.517151	0.461433	0.579598
CYB5B	<0.001	0.401594	0.346141	0.465931
CYB5R1	<0.001	0.368038	0.297721	0.454961
CYB5R2	0.000165	0.785018	0.692149	0.890347
CYB5R3	1.61E-06	0.487373	0.363341	0.653746
CYB5R4	<0.001	0.433637	0.379505	0.49549
CYBA	8.93E-09	1.806857	1.476879	2.210562
CYBB	1.56E-13	0.485533	0.400778	0.58821
CYBRD1	<0.001	0.550201	0.49994	0.605515
CYC1	4.07E-09	0.596597	0.502258	0.708655
CYCS	<0.001	0.459805	0.405985	0.52076
CYFIP1	<0.001	0.479621	0.421592	0.545639
CYLD	<0.001	0.423687	0.370026	0.485129
CYP11A1	0.000739	1.278004	1.10831	1.47368
CYP11B1	8.10E-06	1.549062	1.278166	1.877372
CYP11B2	2.55E-15	1.608958	1.430103	1.810181
CYP17A1	0.058725	1.175591	0.994051	1.390284

(*Continued*)

Table S1: *Continued*

Gene	*P* value	HR	lower 95% CI	upper 95% CI
CYP19A1	0.023482	0.737418	0.56659	0.959753
CYP1A1	1.04E-11	1.22323	1.154218	1.296369
CYP1A2	0.005707	1.376389	1.097397	1.726308
CYP1B1	2.81E-12	0.646132	0.571625	0.73035
CYP20A1	<0.001	0.367314	0.313812	0.429937
CYP24A1	0.002396	0.913294	0.861357	0.968363
CYP26A1	0.12945	0.94592	0.880325	1.016402
CYP26B1	0.065291	1.17008	0.990104	1.382771
CYP27A1	<0.001	0.494686	0.423681	0.577592
CYP27B1	1.31E-13	0.633451	0.561343	0.714823
CYP2A13	3.74E-07	1.342634	1.198414	1.50421
CYP2A6	5.92E-05	0.610487	0.479816	0.776745
CYP2A7	6.63E-12	0.645331	0.56948	0.731286
CYP2B6	<0.001	0.296915	0.238486	0.369659
CYP2C18	4.95E-09	0.746322	0.676618	0.823206
CYP2C19	<0.001	0.468999	0.410193	0.536235
CYP2C8	<0.001	0.332468	0.269294	0.410463
CYP2C9	8.65E-14	0.43196	0.34647	0.538545
CYP2D6	4.40E-07	1.551098	1.308159	1.839152
CYP2E1	<0.001	0.38601	0.322155	0.462522
CYP2F1	1.07E-14	0.582534	0.507963	0.668054
CYP2J2	<0.001	0.575834	0.516835	0.641569
CYP2R1	<0.001	0.380093	0.316842	0.455972
CYP2U1	<0.001	0.178135	0.130996	0.242237
CYP2W1	1.89E-09	0.569767	0.474229	0.684551
CYP39A1	<0.001	0.539052	0.485232	0.598841
CYP3A4	0.000781	0.647449	0.50241	0.834359
CYP3A43	<0.001	0.452071	0.381857	0.535194
CYP3A5	<0.001	0.601257	0.543255	0.665451
CYP46A1	<0.001	0.540766	0.47056	0.621447
CYP4A11	2.15E-12	0.438683	0.34858	0.552075
CYP4B1	1.11E-16	0.789111	0.746556	0.834093
CYP4F11	8.59E-08	1.184137	1.113095	1.259712
CYP4F12	<0.001	0.626996	0.565665	0.694976
CYP4F2	3.70E-07	1.210317	1.124443	1.30275
CYP4F3	0.577973	0.982588	0.923628	1.045312
CYP4F8	0.224706	1.08231	0.952571	1.229719
CYP7A1	0.1835	0.932019	0.840173	1.033904
CYP7B1	<0.001	0.586093	0.52996	0.648171
CYSLTR1	<0.001	0.555129	0.504378	0.610987

(*Continued*)

Table S1: *Continued*

Gene	*P* value	HR	lower 95% CI	upper 95% CI
CYSLTR2	0.25432	0.913583	0.782092	1.06718
CYTH1	<0.001	0.283809	0.214082	0.376245
CYTH2	<0.001	0.238749	0.183653	0.310374
CYTH3	0.612709	0.942923	0.751022	1.183859
CYTH4	2.01E-11	0.384429	0.290705	0.50837
DAAM1	<0.001	0.482405	0.431956	0.538745
DAD1	<0.001	0.388662	0.325046	0.464729
DAGLA	2.47E-05	1.359573	1.178712	1.568185
DAK	<0.001	0.228282	0.178879	0.291328
DAO	<0.001	0.254555	0.20337	0.318622
DAPK1	<0.001	0.446981	0.384321	0.519857
DAPK2	<0.001	0.408345	0.344418	0.484137
DARS	<0.001	0.448544	0.390381	0.515372
DARS2	1.22E-06	0.617854	0.508651	0.750502
DBH	7.34E-11	1.524495	1.342835	1.730729
DBI	<0.001	0.362903	0.299972	0.439037
DBT	<0.001	0.353867	0.298757	0.419143
DCHS1	0.018241	0.789117	0.648251	0.960594
DCHS2	0.831711	0.982576	0.835524	1.15551
DCK	<0.001	0.472686	0.422266	0.529127
DCLK1	<0.001	0.582877	0.53257	0.637936
DCLK2	0.076646	0.826372	0.669095	1.02062
DCP2	<0.001	0.401623	0.346819	0.465088
DCPS	1.24E-13	0.400176	0.314108	0.509826
DCT	<0.001	0.333217	0.267412	0.415215
DCTD	<0.001	0.354131	0.30019	0.417764
DCTPP1	<0.001	0.419509	0.346054	0.508555
DCX	0.801091	1.016781	0.893307	1.157322
DCXR	2.81E-13	0.434775	0.347686	0.543677
DDAH1	<0.001	0.364895	0.309418	0.430318
DDAH2	9.67E-08	0.523573	0.412758	0.66414
DDB1	0.555934	1.1354	0.744047	1.732597
DDB2	<0.001	0.443141	0.376879	0.521052
DDC	0.218446	0.941661	0.855695	1.036263
DDHD2	<0.001	0.52632	0.47521	0.582927
DDO	4.45E-10	0.541862	0.446958	0.656917
DDOST	<0.001	0.262796	0.202767	0.340597
DDR2	<0.001	0.476258	0.419543	0.540639
DDX1	<0.001	0.467838	0.415135	0.527231
DDX10	1.30E-06	0.602627	0.490885	0.739807

(*Continued*)

Table S1: *Continued*

Gene	*P* value	HR	lower 95% CI	upper 95% CI
DDX11	0.010593	1.219815	1.047416	1.420591
DDX17	<0.001	0.530966	0.470597	0.599079
DDX18	<0.001	0.46427	0.41137	0.523972
DDX19A	<0.001	0.225961	0.176677	0.288994
DDX21	<0.001	0.526984	0.473009	0.587118
DDX23	<0.001	0.404304	0.34827	0.469354
DDX24	<0.001	0.40774	0.342398	0.485551
DDX25	<0.001	0.455384	0.389219	0.532797
DDX27	<0.001	0.390321	0.332342	0.458414
DDX28	0.052677	0.766575	0.585835	1.003078
DDX31	<0.001	0.23089	0.182468	0.292163
DDX39A	4.17E-14	0.526016	0.445271	0.621403
DDX3X	<0.001	0.314735	0.260253	0.380621
DDX3Y	0.008358	0.893695	0.82208	0.971548
DDX4	0.431365	0.953251	0.846086	1.073988
DDX41	<0.001	0.457019	0.393794	0.530396
DDX42	<0.001	0.40471	0.348358	0.470178
DDX43	2.46E-12	0.738841	0.678851	0.804133
DDX46	<0.001	0.46383	0.412684	0.521315
DDX49	0.036235	1.323902	1.018159	1.721456
DDX50	<0.001	0.295824	0.241942	0.361706
DDX51	3.45E-08	1.62634	1.368284	1.933064
DDX52	<0.001	0.364372	0.310688	0.427331
DDX54	<0.001	0.257729	0.1979	0.335646
DDX56	<0.001	0.276488	0.204685	0.37348
DDX58	<0.001	0.496693	0.441381	0.558936
DDX6	<0.001	0.3394	0.287556	0.400591
DDX60	<0.001	0.649925	0.605502	0.697607
DECR1	<0.001	0.387017	0.333768	0.448762
DECR2	<0.001	0.374706	0.300775	0.466809
DEF6	<0.001	0.311597	0.253666	0.382758
DEF8	<0.001	0.360561	0.297551	0.436915
DEFA4	9.10E-12	0.631408	0.553251	0.720606
DENND5A	<0.001	0.344736	0.29143	0.407793
DERA	<0.001	0.470147	0.414896	0.532756
DGAT1	3.00E-15	0.501139	0.422117	0.594953
DGKA	0.005258	0.79635	0.678656	0.934455
DGKB	0.027607	0.825643	0.696239	0.979099
DGKD	<0.001	0.41322	0.345408	0.494344
DGKE	<0.001	0.293898	0.239564	0.360555

(*Continued*)

Table S1: *Continued*

Gene	*P* value	HR	lower 95% CI	upper 95% CI
DGKG	<0.001	0.410351	0.347736	0.48424
DGKH	<0.001	0.411309	0.352436	0.480016
DGKI	2.70E-05	0.760072	0.668675	0.863962
DGKQ	0.002292	0.721259	0.584633	0.889813
DGKZ	5.56E-08	1.820652	1.4667	2.260021
DGUOK	<0.001	0.268479	0.206984	0.348242
DHCR24	<0.001	0.593018	0.533466	0.659217
DHCR7	1.96E-05	0.697397	0.591046	0.822885
DHDDS	<0.001	0.301904	0.239842	0.380025
DHFR	<0.001	0.49327	0.428055	0.568421
DHODH	<0.001	0.319478	0.263064	0.387989
DHPS	1.66E-12	0.361998	0.273022	0.479972
DHRS2	0.907073	1.005656	0.914785	1.105554
DHRS3	9.34E-14	0.595738	0.519848	0.682708
DHRS9	1.12E-13	0.687685	0.622975	0.759116
DHTKD1	<0.001	0.489106	0.427728	0.559292
DHX15	<0.001	0.489331	0.439794	0.544449
DHX16	<0.001	0.378252	0.31899	0.448525
DHX29	<0.001	0.563619	0.516301	0.615274
DHX30	<0.001	0.124432	0.088049	0.175847
DHX32	<0.001	0.451527	0.396813	0.513785
DHX34	0.094609	1.207494	0.967994	1.506252
DHX35	6.66E-16	0.422329	0.342555	0.520679
DHX38	<0.001	0.247717	0.1871	0.327973
DHX40	<0.001	0.487892	0.439614	0.541473
DHX57	<0.001	0.295551	0.241427	0.36181
DHX58	0.099678	0.870819	0.73861	1.026692
DHX8	1.95E-13	0.277417	0.197099	0.390465
DHX9	<0.001	0.524304	0.470201	0.584634
DIAPH1	<0.001	0.33094	0.269715	0.406063
DIAPH2	<0.001	0.484892	0.429392	0.547565
DIAPH3	0.444955	1.058048	0.915434	1.22288
DIMT1	<0.001	0.324941	0.266355	0.396412
DIO1	<0.001	0.59034	0.525086	0.663702
DIO2	3.60E-10	0.641044	0.557862	0.736629
DIO3	0.000589	1.282934	1.112999	1.478814
DIRAS2	0.115596	1.091926	0.978634	1.218333
DLAT	<0.001	0.490603	0.429195	0.560798
DLD	<0.001	0.498169	0.446298	0.556068
DLG4	4.11E-06	1.575379	1.298335	1.911538

(*Continued*)

Table S1: *Continued*

Gene	*P* value	HR	lower 95% CI	upper 95% CI
DLST	7.30E-06	1.671178	1.335225	2.09166
DMBT1	2.71E-11	0.797355	0.745961	0.85229
DMP1	0.009087	1.244303	1.055865	1.466371
DMPK	2.88E-05	0.648084	0.528884	0.79415
DNA2	<0.001	0.546307	0.483043	0.617857
DNAJC6	<0.001	0.454959	0.398669	0.519197
DNASE1	<0.001	0.237189	0.179922	0.312685
DNASE1L3	1.95E-13	0.534624	0.452432	0.631747
DNM1	0.009923	0.825984	0.714267	0.955174
DNM1L	<0.001	0.43047	0.367381	0.504394
DNM2	2.60E-06	1.71587	1.369933	2.149164
DNM3	<0.001	0.516465	0.459292	0.580754
DNMT1	<0.001	0.391518	0.319677	0.479504
DNMT3A	<0.001	0.345935	0.28883	0.41433
DNMT3B	<0.001	0.543553	0.473785	0.623594
DNMT3L	6.33E-14	1.447956	1.314472	1.594997
DNPEP	<0.001	0.239806	0.187346	0.306956
DNPH1	7.55E-12	2.034006	1.659908	2.492417
DNTT	0.19849	0.8657	0.694864	1.078535
DOC2A	0.233804	1.061781	0.962004	1.171908
DOC2B	0.063051	1.137081	0.993035	1.302022
DOCK1	<0.001	0.278639	0.227643	0.341058
DOCK10	<0.001	0.497721	0.445539	0.556015
DOCK2	3.33E-16	0.435545	0.356832	0.531621
DOCK3	0.236268	0.948255	0.868438	1.035407
DOCK4	<0.001	0.400443	0.344701	0.465199
DOCK5	<0.001	0.41218	0.352784	0.481576
DOCK6	0.128455	1.217662	0.944627	1.569615
DOCK9	<0.001	0.316697	0.261839	0.383047
DOHH	6.03E-06	0.546708	0.420899	0.710122
DOLK	<0.001	0.370506	0.310332	0.442348
DOLPP1	<0.001	0.291917	0.235009	0.362606
DOT1L	0.000249	0.561338	0.412161	0.764508
DPAGT1	<0.001	0.488833	0.41503	0.575759
DPEP1	0.048994	1.150571	1.000618	1.322996
DPEP2	<0.001	0.486287	0.417022	0.567058
DPH5	<0.001	0.392233	0.337053	0.456446
DPM1	<0.001	0.517215	0.467658	0.572022
DPM2	0.000268	0.596587	0.45188	0.787634
DPM3	<0.001	0.386099	0.319986	0.465871

(*Continued*)

Table S1: *Continued*

Gene	*P* value	HR	lower 95% CI	upper 95% CI
DPP4	<0.001	0.715262	0.670868	0.762594
DPYD	<0.001	0.474342	0.416083	0.540758
DPYS	<0.001	0.502872	0.446944	0.565799
DPYSL2	<0.001	0.593283	0.543238	0.647938
DPYSL3	0.083422	0.880177	0.761752	1.017012
DRD1	0.001528	0.824807	0.732187	0.929142
DRD2	0.061673	1.23983	0.989549	1.553415
DRD3	2.02E-13	1.791109	1.533216	2.092381
DRD4	2.55E-15	1.354989	1.256719	1.460942
DRD5	<0.001	0.456671	0.383737	0.543467
DRG1	<0.001	0.385326	0.319582	0.464596
DRG2	0.399612	1.135318	0.845017	1.525349
DSC1	1.07E-05	1.227616	1.12048	1.344996
DSC2	1.39E-12	0.667616	0.597011	0.746571
DSC3	0.041934	1.061865	1.002192	1.125091
DSE	6.19E-07	0.601275	0.492285	0.734395
DSG1	0.83547	1.01119	0.910389	1.123152
DSG2	1.71E-06	0.768543	0.689979	0.856054
DSG3	0.00025	1.090094	1.040913	1.141598
DSPP	<0.001	0.287882	0.235106	0.352505
DSTYK	<0.001	0.311762	0.246184	0.394807
DTYMK	<0.001	0.288585	0.229254	0.36327
DUOX1	1.11E-16	0.526366	0.452379	0.612452
DUOX2	6.85E-07	0.785645	0.714284	0.864134
DUS1L	5.55E-09	0.401669	0.295591	0.545815
DUS4L	<0.001	0.495327	0.439349	0.558437
DUSP1	<0.001	0.453321	0.385412	0.533196
DUSP10	1.54E-12	0.51076	0.423969	0.615317
DUSP12	<0.001	0.380311	0.322276	0.448797
DUSP13	1.34E-08	0.577809	0.478182	0.698192
DUSP14	5.45E-12	0.564166	0.479431	0.663877
DUSP2	<0.001	0.534078	0.470309	0.606493
DUSP21	9.81E-09	1.456249	1.280678	1.655891
DUSP22	<0.001	0.511734	0.455511	0.574898
DUSP26	<0.001	0.216974	0.167032	0.281849
DUSP3	0.287564	0.86247	0.656632	1.132833
DUSP4	0.165512	0.936557	0.853694	1.027462
DUSP5	2.28E-10	0.64933	0.5682	0.742044
DUSP6	<0.001	0.640047	0.583615	0.701937
DUSP7	0.092408	1.188633	0.971943	1.453632

(*Continued*)

Table S1: *Continued*

Gene	*P* value	HR	lower 95% CI	upper 95% CI
DUSP9	0.888485	1.009345	0.886289	1.149487
DUT	<0.001	0.420173	0.357022	0.494495
DYNLL1	3.72E-14	0.407163	0.322654	0.513808
DYRK1A	<0.001	0.299426	0.23079	0.388474
DYRK1B	9.13E-07	1.512628	1.282271	1.784369
DYRK2	<0.001	0.481186	0.42235	0.548217
DYRK3	<0.001	0.348714	0.283908	0.428313
DYRK4	<0.001	0.364199	0.302979	0.437789
DZIP3	<0.001	0.518252	0.466665	0.575542
EBP	<0.001	0.384004	0.316626	0.46572
ECH1	<0.001	0.401606	0.342053	0.471528
ECHDC1	<0.001	0.548132	0.498566	0.602626
ECHS1	<0.001	0.418477	0.3584	0.488625
ECI1	<0.001	0.420499	0.360507	0.490473
ECI2	<0.001	0.49953	0.441182	0.565595
ECSIT	<0.001	0.331017	0.263299	0.416151
EDF1	<0.001	0.237646	0.183164	0.308334
EDN1	<0.001	0.535016	0.473331	0.60474
EDNRA	<0.001	0.574292	0.522452	0.631274
EDNRB	<0.001	0.5721	0.520319	0.629033
EEA1	<0.001	0.538647	0.488943	0.593403
EEF1A1	<0.001	0.200194	0.159944	0.250572
EEF1A2	0.146583	1.065849	0.97792	1.161683
EEF1D	<0.001	0.312941	0.254331	0.385058
EEF2	4.54E-10	0.342314	0.244374	0.479506
EFHC1	<0.001	0.44951	0.396045	0.510193
EFTUD1	<0.001	0.428445	0.373006	0.492124
EGF	<0.001	0.67374	0.617939	0.73458
EGFR	4.13E-14	0.456315	0.372296	0.559295
EGLN1	<0.001	0.395237	0.32546	0.479973
EGLN3	0.011614	0.884162	0.803538	0.972876
EHD2	1.22E-09	0.569072	0.474473	0.682532
EHHADH	<0.001	0.545903	0.495315	0.601658
EHMT1	<0.001	0.275089	0.221746	0.341265
EHMT2	0.04117	0.755447	0.577152	0.988821
EIF2AK1	<0.001	0.387201	0.326996	0.45849
EIF2AK2	<0.001	0.355635	0.295584	0.427886
EIF2AK3	<0.001	0.27936	0.228215	0.341968
EIF2B1	<0.001	0.296314	0.24456	0.35902
EIF2B2	<0.001	0.423474	0.362588	0.494585

(*Continued*)

Table S1: *Continued*

Gene	*P* value	HR	lower 95% CI	upper 95% CI
EIF2B3	<0.001	0.323342	0.269018	0.388635
EIF2B4	<0.001	0.250742	0.197236	0.318764
EIF2B5	<0.001	0.392281	0.333425	0.461526
EIF2S1	<0.001	0.440409	0.384399	0.504581
EIF2S2	<0.001	0.408586	0.348651	0.478824
EIF2S3	<0.001	0.412832	0.355252	0.479745
EIF4A3	<0.001	0.43283	0.368357	0.508588
EIF5	<0.001	0.448895	0.397344	0.507135
EIF5A	2.16E-10	1.563362	1.361947	1.794563
EIF5A2	<0.001	0.571725	0.505987	0.646003
EIF5B	<0.001	0.51636	0.462279	0.576768
ELMO1	0.00487	0.735694	0.594158	0.910947
ELMO2	<0.001	0.246328	0.194392	0.312141
ELMO3	1.06E-09	0.60471	0.51446	0.710793
ELN	0.087317	0.860601	0.724538	1.022216
ELOVL2	4.70E-11	0.579202	0.49225	0.681512
ELOVL4	<0.001	0.609163	0.545212	0.680614
ELOVL5	<0.001	0.285778	0.232458	0.351328
ELOVL6	<0.001	0.598225	0.537294	0.666067
ELP3	<0.001	0.410294	0.352023	0.47821
ELSPBP1	1.03E-14	0.613971	0.542587	0.694746
EMG1	<0.001	0.459461	0.390484	0.540622
ENDOG	0.009609	0.753021	0.607529	0.933354
ENO1	1.23E-06	1.496705	1.271659	1.761578
ENO2	0.439375	1.056641	0.918921	1.215002
ENO3	3.33E-08	0.578886	0.476809	0.702816
ENOPH1	<0.001	0.463104	0.412686	0.519681
ENOSF1	<0.001	0.463466	0.408752	0.525503
ENOX1	<0.001	0.3973	0.339917	0.46437
ENOX2	<0.001	0.288155	0.235423	0.352698
ENPEP	<0.001	0.59898	0.546355	0.656674
ENPP1	2.67E-10	0.524969	0.429833	0.641161
ENPP2	<0.001	0.541267	0.489081	0.599022
ENTPD1	<0.001	0.461612	0.405682	0.525253
ENTPD2	<0.001	1.673209	1.500194	1.866177
ENTPD3	<0.001	0.579158	0.521693	0.642952
ENTPD4	<0.001	0.314225	0.258168	0.382452
ENTPD5	<0.001	0.315657	0.255862	0.389426
ENTPD6	<0.001	0.303617	0.242783	0.379694
ENTPD7	<0.001	0.460954	0.394858	0.538114

(*Continued*)

Table S1: *Continued*

Gene	*P* value	HR	lower 95% CI	upper 95% CI
EOGT	<0.001	0.572585	0.524913	0.624586
EP300	<0.001	0.562286	0.505504	0.625447
EPB42	1.76E-09	1.652886	1.403355	1.946787
EPHA1	3.23E-11	0.477095	0.383422	0.593653
EPHA2	<0.001	0.629986	0.57088	0.695213
EPHA3	<0.001	0.350842	0.292044	0.421476
EPHA4	<0.001	0.524049	0.461553	0.595007
EPHA5	0.000188	0.687369	0.564609	0.836821
EPHA7	0.000928	0.783418	0.678036	0.90518
EPHB1	0.462151	0.951876	0.834616	1.085611
EPHB2	0.035667	0.828286	0.694772	0.987457
EPHB3	0.684326	0.965218	0.813782	1.144835
EPHB4	<0.001	0.405824	0.33431	0.492637
EPHB6	2.35E-10	0.517914	0.422543	0.634811
EPHX1	0.12882	0.930277	0.847436	1.021217
EPHX2	1.72E-12	0.675573	0.605836	0.753337
EPM2A	<0.001	0.252558	0.199093	0.320381
EPRS	<0.001	0.473447	0.417454	0.536951
EPX	2.52E-12	0.584499	0.50293	0.679299
ERAL1	4.18E-07	0.519354	0.40296	0.669367
ERAP1	<0.001	0.427948	0.373056	0.490919
ERBB2	0.086103	0.832977	0.676081	1.026282
ERBB3	<0.001	0.452041	0.376324	0.542993
ERBB4	<0.001	0.425849	0.365176	0.496603
ERC1	<0.001	0.234401	0.17947	0.306146
ERCC2	7.11E-13	0.330613	0.244375	0.447284
ERCC3	<0.001	0.400323	0.346784	0.462127
ERCC4	<0.001	0.419009	0.364447	0.481738
ERCC6L	0.021041	0.867588	0.768975	0.978847
ERN1	<0.001	0.323615	0.272773	0.383934
ERN2	<0.001	0.324224	0.258727	0.406301
ERO1L	1.22E-15	0.659098	0.595122	0.729951
ERO1LB	<0.001	0.591792	0.539861	0.648718
ERP44	<0.001	0.370418	0.317677	0.431916
ESD	<0.001	0.273223	0.223671	0.333751
ESR1	<0.001	0.238215	0.17267	0.328641
ESR2	0.043141	0.817391	0.672297	0.993799
ESYT1	<0.001	0.405408	0.334734	0.491003
ETF1	<0.001	0.434761	0.381677	0.495229
ETFA	<0.001	0.470462	0.413912	0.534739

(*Continued*)

Table S1: *Continued*

Gene	*P* value	HR	lower 95% CI	upper 95% CI
ETFB	1.11E-15	0.587462	0.515846	0.669021
ETFDH	<0.001	0.509266	0.459534	0.564379
ETNK1	<0.001	0.486225	0.432598	0.546501
ETNK2	0.006014	0.820616	0.712656	0.944932
EWSR1	<0.001	0.371624	0.312765	0.441558
EXOC2	<0.001	0.491841	0.438976	0.551072
EXOC7	<0.001	0.16724	0.123572	0.226341
EXT1	<0.001	0.534088	0.474882	0.600675
EXT2	<0.001	0.407272	0.348937	0.47536
EXTL1	0.393929	1.060012	0.927086	1.211997
EXTL2	<0.001	0.481673	0.428589	0.541333
EXTL3	<0.001	0.295738	0.235714	0.371046
EYA1	0.000581	0.859071	0.787855	0.936724
EYA2	0.000867	0.864622	0.793688	0.941894
EYA3	<0.001	0.26915	0.213021	0.340068
EYA4	1.46E-07	0.679128	0.587903	0.784508
EZH1	<0.001	0.322787	0.270317	0.38544
EZH2	2.90E-14	0.695233	0.633041	0.763535
F10	2.95E-12	0.588702	0.507321	0.683137
F12	2.00E-15	1.778841	1.543212	2.050449
F13A1	<0.001	0.684304	0.633799	0.738833
F5	9.97E-12	0.649372	0.57346	0.735333
F7	7.21E-09	0.530379	0.427849	0.657479
F8	<0.001	0.506226	0.455202	0.562969
F9	1.68E-14	0.57906	0.503633	0.665783
FA2H	2.59E-10	0.613879	0.527691	0.714144
FAAH	<0.001	0.348126	0.288727	0.419745
FABP2	0.147255	0.901168	0.782865	1.037348
FABP3	0.000536	0.79411	0.696948	0.904818
FABP6	0.378337	1.08302	0.906938	1.293289
FADS2	2.14E-10	1.798165	1.500328	2.155127
FADS3	2.83E-12	0.499528	0.411138	0.606921
FAH	2.54E-14	0.497965	0.416206	0.595784
FAHD2A	<0.001	0.215335	0.169224	0.274009
FAM20B	<0.001	0.410473	0.345904	0.487096
FANCL	<0.001	0.567473	0.51906	0.6204
FAR2	<0.001	0.441062	0.377641	0.515133
FARP1	<0.001	0.360555	0.298073	0.436134
FARP2	<0.001	0.206913	0.161144	0.26568
FARS2	<0.001	0.206908	0.157655	0.271548

(*Continued*)

Table S1: *Continued*

Gene	*P* value	HR	lower 95% CI	upper 95% CI
FARSA	6.47E-12	0.413555	0.321451	0.53205
FASN	2.20E-06	1.456515	1.246527	1.701876
FBL	2.70E-10	0.55254	0.459625	0.664238
FBLN2	1.83E-11	0.66317	0.588294	0.747576
FBN1	<0.001	0.600871	0.547677	0.659232
FBN2	1.79E-08	0.638781	0.546522	0.746615
FBP1	<0.001	0.67504	0.625308	0.728728
FBP2	4.77E-14	1.455663	1.320272	1.604938
FCN2	1.68E-07	0.585985	0.479646	0.7159
FDFT1	<0.001	0.468294	0.402065	0.545432
FDPS	<0.001	0.394179	0.327606	0.47428
FDX1	<0.001	0.430885	0.37492	0.495203
FDXR	<0.001	0.405539	0.333514	0.493118
FECH	<0.001	0.340129	0.282864	0.408987
FER	<0.001	0.369567	0.315194	0.433321
FES	<0.001	0.340958	0.279631	0.415736
FFAR2	0.912713	0.993354	0.881705	1.119141
FGD1	0.359771	1.105358	0.892072	1.369639
FGD2	1.33E-07	0.48612	0.37184	0.635523
FGD6	<0.001	0.460981	0.403135	0.527127
FGF1	<0.001	0.446348	0.373617	0.533237
FGF2	<0.001	0.43297	0.375336	0.499454
FGF23	0.010361	1.195405	1.04292	1.370186
FGF3	3.44E-15	1.404139	1.290361	1.52795
FGF4	0.650203	1.051303	0.846874	1.30508
FGF5	1.54E-08	1.454766	1.277583	1.656521
FGF6	0.001521	1.326059	1.113777	1.5788
FGF7	<0.001	0.446683	0.371601	0.536935
FGF8	4.37E-05	1.271549	1.133177	1.426818
FGFBP1	0.204666	1.051513	0.972983	1.136382
FGFR1	2.22E-16	0.341245	0.264175	0.4408
FGFR2	0.000243	0.674678	0.546782	0.83249
FGFR3	1.02E-12	0.659029	0.587641	0.739088
FGFR4	0.007948	1.242602	1.058463	1.458775
FGG	0.407554	0.981987	0.940642	1.02515
FGR	<0.001	0.53149	0.464616	0.607988
FH	0.004799	0.784883	0.663281	0.928779
FHIT	<0.001	0.426254	0.352655	0.515213
FICD	<0.001	0.403593	0.338512	0.481185
FKBP4	0.0051	1.305227	1.083243	1.5727

(*Continued*)

Table S1: *Continued*

Gene	*P* value	HR	lower 95% CI	upper 95% CI
FKBP5	5.36E-07	0.740163	0.658018	0.832563
FKBP8	6.57E-11	1.474131	1.312052	1.656231
FLAD1	<0.001	0.392078	0.317228	0.484588
FLT1	<0.001	0.396128	0.334547	0.469043
FLT3	<0.001	0.394194	0.34076	0.456008
FLT4	8.89E-09	0.442723	0.33537	0.58444
FLVCR2	<0.001	0.388338	0.326647	0.461679
FMO1	1.36E-07	0.763147	0.690159	0.843854
FMO2	<0.001	0.619687	0.571979	0.671374
FMO3	<0.001	0.677175	0.630259	0.727582
FMO4	<0.001	0.408739	0.354771	0.470918
FMO5	<0.001	0.668102	0.622187	0.717404
FMO6P	1.33E-05	0.776507	0.692952	0.870138
FMOD	<0.001	0.549201	0.47809	0.63089
FN1	<0.001	0.504751	0.435986	0.584363
FN3K	4.24E-10	1.636271	1.40197	1.909729
FN3KRP	<0.001	0.334478	0.279112	0.400827
FNBP1	<0.001	0.448898	0.386757	0.521022
FNBP1L	<0.001	0.615497	0.57104	0.663415
FNTA	<0.001	0.386828	0.330005	0.453435
FOLH1	0.015943	0.83115	0.715095	0.96604
FOLR1	4.88E-15	0.82097	0.781417	0.862525
FOLR2	2.06E-05	0.640914	0.522259	0.786526
FOLR3	0.843791	0.98537	0.851013	1.14094
FOXK2	<0.001	0.216218	0.167697	0.278779
FOXO4	<0.001	0.398433	0.327514	0.484709
FOXRED2	2.72E-07	0.602535	0.496722	0.730889
FPGS	<0.001	0.304011	0.232295	0.397868
FPGT	<0.001	0.60434	0.558833	0.653553
FPR1	0.015277	0.813917	0.689176	0.961236
FPR2	0.385743	0.921617	0.766383	1.108294
FPR3	8.78E-14	0.612587	0.538562	0.696787
FRAS1	<0.001	0.391228	0.325958	0.469569
FRK	<0.001	0.561104	0.511213	0.615864
FSHR	4.91E-07	1.485726	1.273332	1.733546
FTCD	6.83E-13	1.834741	1.554721	2.165196
FTH1	<0.001	0.426201	0.360836	0.503407
FTL	<0.001	0.197783	0.143713	0.272194
FTSJ1	8.07E-09	0.451705	0.344789	0.591774
FTSJ2	<0.001	0.389623	0.333053	0.455802

(*Continued*)

Table S1: *Continued*

Gene	*P* value	HR	lower 95% CI	upper 95% CI
FTSJ3	<0.001	0.360992	0.292004	0.44628
FUCA1	<0.001	0.421811	0.348034	0.511227
FURIN	0.073267	1.161059	0.986025	1.367164
FUT1	<0.001	0.503161	0.442638	0.571961
FUT2	3.33E-16	0.600152	0.530812	0.678551
FUT3	0.099324	0.887247	0.769591	1.022892
FUT5	0.212836	1.115616	0.939209	1.325157
FUT6	0.991908	1.000994	0.826088	1.212932
FUT7	3.34E-09	1.464962	1.290833	1.662581
FUT8	5.75E-13	0.549722	0.467162	0.646872
FUT9	0.317777	0.943745	0.84241	1.05727
FXN	<0.001	0.278501	0.220045	0.352484
FXYD1	3.87E-06	0.773298	0.693365	0.862446
FXYD2	<0.001	0.411276	0.34375	0.492066
FXYD3	0.042349	0.90413	0.8203	0.996526
FYN	<0.001	0.472703	0.417073	0.535753
FZD1	<0.001	0.53862	0.482088	0.60178
FZD10	0.043454	0.922143	0.852377	0.99762
FZD2	8.47E-13	0.560808	0.47862	0.657108
FZD3	<0.001	0.563733	0.510484	0.622537
FZD4	<0.001	0.314605	0.248178	0.398812
FZD5	<0.001	0.525273	0.4705	0.586422
FZD6	<0.001	0.604142	0.55631	0.656087
FZD7	<0.001	0.651844	0.592887	0.716663
FZD9	0.00365	1.175405	1.054058	1.310723
G3BP1	<0.001	0.375627	0.318152	0.443483
G6PC	0.96928	1.003725	0.830665	1.212842
G6PC2	2.04E-10	0.521708	0.426898	0.637574
G6PC3	0.086922	0.812176	0.640038	1.03061
G6PD	0.871112	0.990271	0.879956	1.114415
GAA	4.43E-10	0.549845	0.455645	0.663519
GABARAP	<0.001	0.279933	0.220312	0.355689
GABARAPL1	1.11E-16	0.473408	0.39647	0.565277
GABARAPL2	<0.001	0.361927	0.309059	0.423838
GABBR1	<0.001	0.348829	0.293429	0.414688
GABBR2	0.000182	0.769584	0.670962	0.882702
GABRA1	0.792962	1.020158	0.878908	1.184108
GABRA2	6.90E-08	0.564204	0.458263	0.694636
GABRA3	0.000138	1.263251	1.1202	1.424571
GABRA4	<0.001	0.341852	0.278545	0.419549

(*Continued*)

Table S1: *Continued*

Gene	*P* value	HR	lower 95% CI	upper 95% CI
GABRA5	1.55E-15	0.375875	0.295513	0.478091
GABRA6	3.05E-09	1.44071	1.276891	1.625546
GABRB1	3.39E-11	0.58511	0.499359	0.685585
GABRB2	<0.001	0.501726	0.441053	0.570745
GABRB3	2.60E-11	0.599167	0.515415	0.69653
GABRD	<0.001	1.618248	1.45293	1.802376
GABRG2	0.036743	0.84111	0.715046	0.989399
GABRG3	4.03E-09	0.561173	0.462918	0.680283
GABRP	0.000131	0.891936	0.841171	0.945764
GABRQ	6.03E-05	0.68886	0.574179	0.826445
GABRR1	3.50E-06	0.78851	0.713192	0.871782
GABRR2	3.36E-06	0.667767	0.563198	0.791752
GAD1	0.011581	0.887613	0.809149	0.973686
GAD2	0.027635	1.335525	1.032364	1.727712
GAK	<0.001	0.381037	0.320729	0.452684
GAL3ST1	0.000812	0.845155	0.7659	0.932612
GAL3ST4	1.28E-13	0.518476	0.435763	0.616889
GALC	<0.001	0.511045	0.457182	0.571252
GALE	1.80E-13	0.449346	0.363161	0.555985
GALK1	2.81E-10	1.698908	1.441	2.002975
GALK2	<0.001	0.3531	0.300169	0.415364
GALNS	2.10E-09	0.452627	0.349218	0.586656
GALNT1	<0.001	0.531166	0.479638	0.58823
GALNT10	<0.001	0.594955	0.537279	0.658822
GALNT11	<0.001	0.545845	0.492525	0.604936
GALNT12	<0.001	0.629898	0.580272	0.683769
GALNT14	0.019769	1.098097	1.014997	1.188001
GALNT2	<0.001	0.452944	0.385422	0.532294
GALNT3	<0.001	0.498968	0.444571	0.560022
GALNT6	0.000154	1.190422	1.087651	1.302904
GALNT7	<0.001	0.589768	0.539704	0.644476
GALNT8	0.793483	0.981075	0.850314	1.131945
GALT	<0.001	0.27266	0.220916	0.336523
GAMT	6.33E-12	1.852322	1.553674	2.208375
GANAB	<0.001	2.432416	1.982039	2.98513
GAPDH	5.27E-05	1.820002	1.361458	2.432987
GAPDHS	0.659903	1.032496	0.895425	1.190551
GAPVD1	<0.001	0.34278	0.290301	0.404745
GARS	<0.001	0.481782	0.419713	0.55303
GART	<0.001	0.410569	0.346735	0.486156

(*Continued*)

Table S1: *Continued*

Gene	*P* value	HR	lower 95% CI	upper 95% CI
GAS6	<0.001	0.538901	0.472688	0.614389
GATC	<0.001	0.356305	0.294654	0.430856
GATM	0.933348	0.989884	0.78002	1.256213
GBA3	<0.001	0.451712	0.375853	0.542881
GBF1	0.969589	0.993218	0.700003	1.409253
GBP1	5.55E-15	0.652642	0.586399	0.726369
GBP2	<0.001	0.587108	0.526814	0.654303
GCA	<0.001	0.584928	0.538517	0.635339
GCAT	4.69E-07	0.616793	0.511096	0.744348
GCDH	<0.001	0.185091	0.138538	0.247287
GCGR	0.010852	0.813	0.693291	0.95338
GCH1	<0.001	0.584703	0.529437	0.645738
GCHFR	0.31575	1.107851	0.906943	1.353264
GCK	7.35E-14	1.390479	1.275436	1.515899
GCKR	2.72E-07	0.642118	0.542354	0.760232
GCLC	1.62E-06	0.763209	0.683417	0.852318
GCLM	3.00E-15	0.661926	0.597457	0.733351
GCNT1	<0.001	0.632293	0.56828	0.703516
GCNT2	<0.001	0.464585	0.410024	0.526407
GCNT3	6.22E-15	0.769774	0.720785	0.822093
GCNT4	3.99E-09	0.629882	0.540004	0.734719
GDE1	<0.001	0.395316	0.33714	0.463529
GDI1	<0.001	0.390105	0.329945	0.461233
GDI2	<0.001	0.39868	0.333647	0.476389
GDNF	0.030007	1.201778	1.01795	1.418803
GDPD2	6.33E-15	1.469824	1.334204	1.61923
GEM	<0.001	0.599595	0.549678	0.654044
GFER	0.000435	1.576359	1.223308	2.031302
GFM1	<0.001	0.380002	0.322759	0.447397
GFPT1	<0.001	0.499189	0.445184	0.559746
GFPT2	2.58E-14	0.624146	0.552865	0.704617
GGA1	<0.001	0.238633	0.182055	0.312795
GGA2	<0.001	0.197294	0.152713	0.25489
GGA3	<0.001	0.255743	0.20518	0.318767
GGCT	<0.001	0.521239	0.46937	0.578841
GGCX	<0.001	0.382469	0.313939	0.465958
GGH	1.39E-10	0.744121	0.679887	0.814423
GGPS1	<0.001	0.459125	0.407349	0.517483
GGT5	0.127606	1.087231	0.976332	1.210726
GIF	2.80E-11	0.644523	0.566331	0.733512

(*Continued*)

Table S1: *Continued*

Gene	*P* value	HR	lower 95% CI	upper 95% CI
GIMAP4	<0.001	0.527734	0.469593	0.593073
GIMAP6	<0.001	0.592923	0.536038	0.655845
GJA1	<0.001	0.677939	0.623387	0.737264
GK	<0.001	0.4637	0.402224	0.534571
GK2	2.19E-07	0.726296	0.643554	0.819676
GK3P	<0.001	0.46904	0.410954	0.535336
GLA	<0.001	0.472839	0.406042	0.550624
GLCE	<0.001	0.586657	0.536579	0.641409
GLDC	0.074471	0.90202	0.805392	1.010241
GLI3	9.97E-10	0.580935	0.488049	0.691498
GLO1	<0.001	0.471082	0.416533	0.532774
GLRA1	0.01383	1.174674	1.033355	1.33532
GLRA2	1.18E-12	1.521806	1.355421	1.708615
GLRA3	2.26E-07	0.613796	0.510222	0.738395
GLRB	<0.001	0.527344	0.475548	0.584781
GLRX	<0.001	0.501319	0.442393	0.568093
GLRX2	<0.001	0.447146	0.390805	0.51161
GLS	<0.001	0.433743	0.380383	0.494588
GLS2	1.52E-07	0.709517	0.624206	0.806487
GLTP	7.19E-13	0.494516	0.407991	0.599391
GLUD1	1.52E-14	0.445267	0.362253	0.547305
GLUD2	<0.001	0.362293	0.299229	0.438648
GLUL	2.05E-13	0.549576	0.468445	0.644759
GLYAT	0.044745	0.74945	0.565489	0.993257
GM2A	0.002997	0.736961	0.602437	0.901524
GMDS	<0.001	0.470149	0.395004	0.559591
GMIP	2.24E-07	0.542358	0.430234	0.683701
GMPPA	<0.001	0.403593	0.329731	0.494
GMPR	<0.001	0.480898	0.420324	0.550202
GMPR2	<0.001	0.361515	0.305039	0.428449
GMPS	<0.001	0.477298	0.416666	0.546752
GNA11	1.93E-12	0.286551	0.202339	0.405812
GNA12	<0.001	0.420017	0.360882	0.488842
GNA13	<0.001	0.499755	0.443395	0.563278
GNA14	<0.001	0.52054	0.458587	0.590863
GNA15	4.74E-12	0.642936	0.567258	0.72871
GNAI1	<0.001	0.66602	0.621345	0.713907
GNAI2	1.20E-08	0.555704	0.454046	0.680122
GNAI3	<0.001	0.531263	0.478751	0.589534
GNAL	7.68E-10	0.602247	0.512397	0.707852

(*Continued*)

Table S1: *Continued*

Gene	*P* value	HR	lower 95% CI	upper 95% CI
GNAO1	<0.001	0.306446	0.239047	0.392848
GNAQ	<0.001	0.441392	0.387056	0.503355
GNAS	<0.001	0.227055	0.178965	0.288068
GNAT1	1.41E-14	1.619938	1.432645	1.831715
GNAT2	0.000324	0.787886	0.691866	0.897232
GNAT3	0.111369	0.911962	0.814151	1.021524
GNAZ	<0.001	0.391935	0.332118	0.462525
GNB1	<0.001	0.478337	0.404756	0.565293
GNB2	1.81E-11	0.468945	0.376017	0.584839
GNB3	0.217349	0.858785	0.67428	1.093778
GNB5	<0.001	0.28415	0.223313	0.361562
GNE	<0.001	0.489613	0.428529	0.559404
GNG10	<0.001	0.462851	0.409071	0.523701
GNG11	<0.001	0.541335	0.482373	0.607504
GNG12	<0.001	0.536956	0.478584	0.602447
GNG13	0.528383	0.957648	0.837107	1.095547
GNG3	2.22E-14	0.480657	0.398274	0.580081
GNG4	0.005544	1.14381	1.040203	1.257735
GNG5	<0.001	0.315132	0.247025	0.402018
GNG7	<0.001	0.253231	0.204406	0.313719
GNGT1	0.018649	0.903445	0.830159	0.983201
GNL1	<0.001	0.214578	0.168238	0.273683
GNL2	<0.001	0.573007	0.525964	0.624257
GNL3L	<0.001	0.256743	0.202361	0.325738
GNMT	<0.001	0.485745	0.425445	0.554592
GNPAT	<0.001	0.418282	0.365438	0.478767
GNPDA1	<0.001	0.452163	0.389802	0.524502
GNPTAB	<0.001	0.309948	0.259184	0.370655
GNRHR	0.766286	0.968672	0.785283	1.194888
GNS	<0.001	0.416277	0.354544	0.48876
GOLGA2	<0.001	0.376131	0.303145	0.466689
GOLGA5	<0.001	0.505249	0.453866	0.562448
GOT1	2.08E-08	0.60768	0.51055	0.723289
GOT2	<0.001	0.412453	0.349038	0.487389
GPAA1	<0.001	0.368498	0.297957	0.455739
GPD1	6.78E-06	1.436796	1.22701	1.682449
GPD1L	<0.001	0.582191	0.532867	0.63608
GPD2	<0.001	0.445932	0.388277	0.512149
GPHN	<0.001	0.397663	0.342015	0.462365
GPI	0.08568	1.159003	0.979493	1.37141

(*Continued*)

Table S1: *Continued*

Gene	*P* value	HR	lower 95% CI	upper 95% CI
GPLD1	<0.001	0.216885	0.168687	0.278854
GPR17	3.18E-05	1.428196	1.207426	1.689332
GPR18	<0.001	0.527427	0.473277	0.587773
GPR6	1.08E-07	0.570861	0.464207	0.70202
GPT	1.68E-11	0.395871	0.302251	0.518489
GPX1	8.49E-07	0.597096	0.486292	0.733148
GPX2	0.005637	1.100185	1.028268	1.177132
GPX3	4.44E-16	0.691217	0.632215	0.755726
GPX4	0.041493	0.75301	0.57326	0.989121
GPX5	0.620601	0.953827	0.791006	1.150164
GPX7	3.00E-15	0.570016	0.495723	0.655442
GRAP	0.000123	1.344628	1.156011	1.56402
GRB2	<0.001	0.297059	0.239153	0.368987
GRHPR	3.27E-12	0.383473	0.292824	0.502186
GRIA1	<0.001	0.501901	0.438531	0.57443
GRIA2	0.108242	1.132363	0.972988	1.317844
GRIA3	1.13E-12	0.436107	0.34697	0.548144
GRIA4	0.324007	1.090177	0.91829	1.294238
GRIK1	3.02E-09	0.557806	0.459937	0.6765
GRIK2	6.22E-15	0.445545	0.363627	0.545918
GRIK3	0.614008	0.951219	0.783208	1.15527
GRIN1	2.35E-08	1.476974	1.288015	1.693654
GRIN2A	5.08E-13	0.542795	0.459865	0.640681
GRIN2B	0.499205	1.068866	0.881115	1.296623
GRIN2C	0.99481	0.999207	0.786682	1.269145
GRIN2D	0.001931	0.744893	0.61836	0.897318
GRK1	6.07E-07	1.404132	1.228842	1.604427
GRM1	0.195011	1.142437	0.934038	1.397332
GRM4	0.002092	1.376508	1.122996	1.687249
GRM5	<0.001	0.428703	0.353004	0.520636
GRM8	0.398112	1.084145	0.898882	1.307592
GRPR	0.070944	1.127971	0.989775	1.285464
GSK3A	3.84E-08	0.510313	0.401498	0.648618
GSK3B	<0.001	0.338104	0.283759	0.402857
GSN	0.171779	1.250114	0.907604	1.721882
GSPT1	<0.001	0.396712	0.341129	0.461352
GSPT2	<0.001	0.596737	0.549486	0.648051
GSR	0.000161	0.725591	0.61425	0.857114
GSS	<0.001	0.371101	0.301963	0.456068
GSTA1	<0.001	0.634426	0.576859	0.697738

(*Continued*)

Table S1: *Continued*

Gene	*P* value	HR	lower 95% CI	upper 95% CI
GSTA3	3.18E-07	0.759882	0.683956	0.844237
GSTA4	<0.001	0.50532	0.451285	0.565825
GSTK1	<0.001	0.384456	0.312676	0.472714
GSTM1	6.60E-08	0.642697	0.547449	0.754518
GSTM2	2.44E-14	0.562118	0.484752	0.651831
GSTM3	<0.001	0.542146	0.479795	0.612601
GSTM4	2.03E-06	0.65876	0.554543	0.782562
GSTM5	<0.001	0.330621	0.274063	0.398851
GSTO1	2.48E-10	0.504886	0.408571	0.623905
GSTP1	7.22E-12	1.680878	1.448889	1.950012
GSTT1	0.026285	0.929806	0.871991	0.991455
GSTT2	1.18E-09	0.725616	0.654381	0.804606
GSTZ1	0.000416	0.710989	0.588309	0.859252
GTF2F2	<0.001	0.531025	0.470565	0.599253
GTF3C4	<0.001	0.323873	0.270594	0.387642
GTPBP1	0.36831	0.861746	0.623157	1.191686
GTPBP2	0.064965	0.821254	0.666271	1.012289
GTPBP3	<0.001	0.266325	0.21186	0.334793
GTPBP4	<0.001	0.451903	0.394831	0.517226
GTPBP6	<0.001	0.353553	0.295863	0.422493
GUCA1A	0.069282	1.157746	0.988505	1.355963
GUCA1B	<0.001	0.383233	0.316577	0.463924
GUCY1A2	<0.001	0.50499	0.452079	0.564093
GUCY1A3	<0.001	0.558788	0.510519	0.611622
GUCY1B2	6.66E-16	0.704953	0.647651	0.767326
GUCY1B3	<0.001	0.473773	0.421373	0.532688
GUCY2C	0.04942	0.897503	0.805733	0.999726
GUCY2D	<0.001	0.547548	0.481222	0.623017
GUCY2F	7.16E-09	1.522134	1.320263	1.754872
GUF1	<0.001	0.492117	0.438463	0.552336
GUK1	<0.001	0.251317	0.198613	0.318006
GULP1	<0.001	0.520854	0.462611	0.586431
GUSB	<0.001	0.314128	0.250672	0.393648
GYG1	<0.001	0.407083	0.338492	0.489572
GYG2	<0.001	0.488104	0.424815	0.560822
GYPB	0.506211	1.078398	0.863242	1.34718
GYS1	<0.001	0.455791	0.386701	0.537224
GYS2	0.207106	1.074489	0.961007	1.201371
GZMB	0.077398	1.091515	0.990428	1.20292
H6PD	1.40E-14	0.302839	0.223405	0.410517

(*Continued*)

Table S1: *Continued*

Gene	*P* value	HR	lower 95% CI	upper 95% CI
HAAO	0.229348	1.177078	0.902309	1.535518
HABP2	<0.001	0.646314	0.589501	0.708602
HADH	<0.001	0.404526	0.352077	0.464787
HADHA	<0.001	0.241615	0.184944	0.315652
HADHB	<0.001	0.396399	0.339272	0.463145
HAGH	<0.001	0.213292	0.163628	0.278028
HAL	<0.001	0.482667	0.408955	0.569664
HAMP	0.254176	0.906174	0.765005	1.073394
HAO1	0.000477	1.279533	1.114275	1.469301
HAO2	<0.001	0.33118	0.261378	0.419622
HAPLN1	5.92E-08	0.664091	0.572737	0.770017
HAPLN2	0.001418	1.267974	1.095915	1.467047
HARS2	<0.001	0.320427	0.26302	0.390363
HAS1	0.819419	0.990131	0.909312	1.078133
HAS2	<0.001	0.640176	0.589502	0.695205
HAT1	<0.001	0.59596	0.548499	0.647528
HBB	2.27E-07	0.753192	0.676535	0.838534
HBD	0.038575	0.877181	0.774763	0.993137
HBE1	1.88E-10	0.571303	0.480918	0.678677
HBEGF	1.86E-07	0.582261	0.475125	0.713557
HBQ1	<0.001	1.415882	1.311631	1.52842
HBS1L	<0.001	0.447767	0.395223	0.507297
HBZ	0.016824	1.153423	1.026042	1.296618
HCCS	<0.001	0.423842	0.359678	0.499451
HCK	4.91E-13	0.628841	0.554513	0.713133
HCN2	0.104892	1.155387	0.97031	1.375767
HCN4	0.001362	0.700318	0.563144	0.870907
HCRT	<0.001	0.390667	0.332089	0.459579
HDAC2	<0.001	0.409801	0.354898	0.473198
HDAC3	<0.001	0.327648	0.268779	0.399411
HDAC4	<0.001	0.329862	0.27166	0.400535
HDAC5	4.55E-15	0.317139	0.237985	0.422619
HDAC9	<0.001	0.389469	0.3299	0.459793
HDC	<0.001	0.415838	0.358383	0.482504
HDGF	2.06E-11	0.447877	0.354111	0.566472
HDHD1	<0.001	0.463595	0.404623	0.531161
HEBP1	<0.001	0.484881	0.427881	0.549474
HEBP2	0.2302	1.141357	0.91965	1.416513
HEMK1	<0.001	0.198303	0.148997	0.263926
HEPH	<0.001	0.49284	0.427695	0.567908

(*Continued*)

Table S1: *Continued*

Gene	*P* value	HR	lower 95% CI	upper 95% CI
HERC1	<0.001	0.376629	0.316499	0.448182
HERC2	<0.001	0.502524	0.446004	0.566207
HEXA	<0.001	0.237275	0.186016	0.30266
HEXB	<0.001	0.444911	0.39068	0.50667
HFE	3.75E-08	0.417208	0.305578	0.569618
HGD	0.00019	0.83128	0.754389	0.916007
HGSNAT	<0.001	0.278895	0.220457	0.352823
HHAT	<0.001	0.470778	0.419669	0.528112
HIBCH	<0.001	0.475079	0.423681	0.532712
HIF1AN	4.36E-06	0.509517	0.382129	0.67937
HINT1	<0.001	0.324876	0.27195	0.388102
HIPK1	<0.001	0.369364	0.311093	0.438549
HIPK2	<0.001	0.263585	0.207306	0.335143
HIPK3	<0.001	0.385005	0.329582	0.449749
HIRIP3	<0.001	0.207671	0.161522	0.267005
HIST1H2AB	<0.001	0.241214	0.184949	0.314595
HIST1H2AC	<0.001	0.655505	0.597079	0.719647
HIST1H2AJ	0.002301	1.262037	1.086642	1.465742
HIST3H2A	4.75E-09	0.603576	0.509733	0.714696
HK1	<0.001	0.348603	0.284568	0.427047
HK2	0.009658	0.834537	0.727696	0.957064
HK3	7.43E-05	0.815046	0.736622	0.90182
HKDC1	4.33E-10	0.604993	0.516673	0.70841
HLA-B	0.000188	0.637976	0.503921	0.807693
HLCS	<0.001	0.372739	0.304339	0.456511
HMBS	1.11E-16	0.421306	0.343562	0.516641
HMGCL	<0.001	0.428609	0.369357	0.497366
HMGCR	<0.001	0.553748	0.502846	0.609804
HMGCS1	2.00E-15	0.605863	0.535358	0.685654
HMGCS2	3.89E-10	0.593444	0.503973	0.698799
HMHA1	<0.001	0.330176	0.275846	0.395205
HMMR	8.66E-15	0.723479	0.666672	0.785126
HMOX1	2.37E-06	1.37784	1.206095	1.574041
HMOX2	<0.001	0.205936	0.157205	0.269773
HNMT	<0.001	0.423366	0.370969	0.483163
HOMER1	<0.001	0.543648	0.486828	0.6071
HOMER2	<0.001	0.584569	0.520439	0.656601
HOMER3	6.66E-16	1.629006	1.447292	1.833535
HP	0.017018	0.928026	0.872803	0.986743
HPCA	0.24043	0.899774	0.754319	1.073278

(*Continued*)

Table S1: *Continued*

Gene	*P* value	HR	lower 95% CI	upper 95% CI
HPCAL1	0.023586	0.793071	0.64884	0.969362
HPCAL4	0.219195	0.8848	0.727869	1.075566
HPD	4.72E-11	1.301163	1.203023	1.407309
HPGD	<0.001	0.728161	0.685575	0.773391
HPGDS	<0.001	0.635058	0.583283	0.69143
HPRT1	9.06E-14	0.506506	0.423553	0.605706
HPSE	<0.001	0.578507	0.51564	0.649039
HPSE2	1.42E-06	1.315919	1.176961	1.471284
HPX	9.47E-09	1.48995	1.30029	1.707274
HRAS	0.002554	0.71653	0.577012	0.889783
HRASLS2	0.090739	1.177022	0.974465	1.421682
HRC	0.00064	1.286128	1.113138	1.486002
HRG	1.79E-10	0.534299	0.440698	0.647782
HRH1	<0.001	0.538004	0.473056	0.611869
HRH2	0.575728	0.946442	0.780496	1.147671
HRH3	0.000217	1.495121	1.208112	1.850313
HRH4	<0.001	0.45448	0.384984	0.536521
HS3ST1	0.042504	1.156222	1.00492	1.330305
HS3ST2	<0.001	0.622327	0.557554	0.694624
HS3ST3A1	1.30E-09	0.709719	0.635299	0.792856
HS3ST3B1	<0.001	0.359853	0.303461	0.426725
HS6ST1	1.09E-08	1.791161	1.46669	2.187414
HSD11B1	<0.001	0.609441	0.550343	0.674884
HSD11B2	5.00E-15	0.67285	0.609295	0.743033
HSD17B1	0.000655	0.672961	0.535873	0.845119
HSD17B10	<0.001	0.416444	0.351805	0.492961
HSD17B11	<0.001	0.591524	0.543683	0.643575
HSD17B12	<0.001	0.28676	0.230658	0.356506
HSD17B14	<0.001	0.404597	0.327781	0.499414
HSD17B2	1.99E-06	0.865201	0.815071	0.918414
HSD17B3	2.02E-05	0.767866	0.680048	0.867024
HSD17B4	<0.001	0.350974	0.290673	0.423785
HSD17B6	<0.001	0.667487	0.621466	0.716915
HSD17B7	<0.001	0.327344	0.273895	0.391224
HSD17B8	<0.001	0.415029	0.353034	0.48791
HSD3B1	0.063974	1.191144	0.989891	1.433314
HSD3B2	0.136262	0.878676	0.741196	1.041656
HSP90AA1	<0.001	0.523532	0.470864	0.582092
HSPG2	0.001779	1.430155	1.142683	1.789947
HTATIP2	1.97E-12	0.474034	0.385046	0.583589

(*Continued*)

Table S1: *Continued*

Gene	*P* value	HR	lower 95% CI	upper 95% CI
HTR1A	0.758902	1.023918	0.880469	1.190737
HTR1B	0.041747	0.879225	0.776764	0.995203
HTR1D	1.12E-06	1.373813	1.208939	1.561173
HTR1E	<0.001	0.247936	0.196465	0.312891
HTR1F	<0.001	0.547431	0.483366	0.619987
HTR2A	<0.001	0.329822	0.273031	0.398427
HTR2B	<0.001	0.625316	0.572442	0.683074
HTR2C	0.003734	1.203145	1.061773	1.36334
HTR3A	0.10653	1.085556	0.982556	1.199354
HTR3B	0.255695	1.075008	0.948957	1.217803
HTR4	0.041615	0.794351	0.636536	0.991293
HTR5A	3.28E-14	1.685876	1.473091	1.929397
HTR6	0.377394	1.099117	0.891066	1.355744
HTR7	0.340401	0.897252	0.717984	1.12128
HUNK	<0.001	0.408989	0.338598	0.494012
HUWE1	<0.001	0.112302	0.077336	0.163078
HYAL1	1.25E-09	0.753864	0.688169	0.82583
HYAL2	<0.001	0.306176	0.250762	0.373837
HYAL3	0.016411	0.836391	0.722829	0.967794
HYAL4	0.902902	1.007524	0.893209	1.13647
HYI	<0.001	0.330747	0.267859	0.4084
IARS	<0.001	0.463951	0.408409	0.527047
IARS2	<0.001	0.490047	0.440272	0.54545
IBTK	<0.001	0.512163	0.46088	0.569151
ICAM1	6.57E-13	0.64161	0.568486	0.72414
ICK	<0.001	0.408418	0.35054	0.475852
ICMT	<0.001	0.261931	0.198629	0.345407
ICT1	8.88E-16	0.383942	0.303989	0.484923
ID3	5.02E-07	0.681407	0.586729	0.791363
IDE	<0.001	0.470634	0.417077	0.531069
IDH1	<0.001	0.436317	0.378229	0.503325
IDH2	3.13E-11	0.47009	0.376203	0.587408
IDH3A	<0.001	0.377003	0.321478	0.442118
IDH3B	<0.001	0.372447	0.313515	0.442456
IDH3G	5.88E-10	0.438755	0.338066	0.569433
IDI1	<0.001	0.426074	0.369668	0.491087
IDO1	1.18E-07	0.79069	0.724873	0.862483
IDS	<0.001	0.333745	0.278233	0.400333
IDUA	0.000651	1.448242	1.17051	1.791874
IFIH1	<0.001	0.504753	0.449492	0.566809

(*Continued*)

Table S1: *Continued*

Gene	*P* value	HR	lower 95% CI	upper 95% CI
IFNB1	0.069102	1.124534	0.99086	1.276243
IFNG	7.68E-09	0.739398	0.667391	0.819175
IGF1R	2.83E-07	0.575613	0.466195	0.710711
IGF2R	2.09E-10	0.573788	0.48344	0.68102
IGHMBP2	<0.001	0.302234	0.235759	0.387452
IGLC1	0.378293	0.938276	0.814288	1.081144
IKBKB	<0.001	0.374981	0.311957	0.450736
IKBKE	1.32E-10	0.406743	0.309125	0.535187
IL1B	5.72E-13	0.643517	0.570818	0.725476
IL3	2.11E-15	1.575489	1.408183	1.762673
IL5	0.910733	1.008122	0.875183	1.161254
IL6	0.106819	0.933444	0.85849	1.014943
ILK	<0.001	0.360328	0.298472	0.435003
ILKAP	<0.001	0.478796	0.419025	0.547094
ILVBL	1.17E-05	0.469902	0.335216	0.658705
IMPA1	<0.001	0.546803	0.49625	0.602507
IMPA2	1.66E-12	0.62341	0.546767	0.710796
IMPAD1	<0.001	0.381282	0.307931	0.472106
IMPDH1	<0.001	0.369027	0.29575	0.460459
IMPDH2	<0.001	0.470036	0.401001	0.550956
IMPG2	0.007716	0.792764	0.668266	0.940456
INPP1	<0.001	0.544687	0.486586	0.609724
INPP4A	<0.001	0.300293	0.244559	0.368729
INPP4B	<0.001	0.616521	0.562698	0.675493
INPP5A	<0.001	0.389869	0.32911	0.461845
INPP5B	<0.001	0.326748	0.264976	0.402921
INPP5D	<0.001	0.31719	0.256971	0.391521
INPP5E	<0.001	0.31915	0.266451	0.382273
INPP5J	0.004077	1.214495	1.063662	1.386717
INPP5K	<0.001	0.155356	0.11338	0.212872
INPPL1	<0.001	0.345182	0.288222	0.413397
INSR	<0.001	0.371996	0.312043	0.443469
INSRR	0.011493	1.199815	1.041743	1.381872
INVS	<0.001	0.301835	0.244389	0.372784
IP6K1	<0.001	0.249605	0.199087	0.312941
IPO13	<0.001	0.311482	0.250829	0.386801
IPO4	2.05E-06	0.596681	0.482138	0.738437
IPO5	<0.001	0.436453	0.383344	0.496919
IPO7	<0.001	0.508553	0.459205	0.563205
IPO8	<0.001	0.354831	0.299179	0.420835

(*Continued*)

Table S1: *Continued*

Gene	*P* value	HR	lower 95% CI	upper 95% CI
IPO9	<0.001	0.34217	0.287359	0.407437
IPPK	<0.001	0.275592	0.216642	0.350583
IQGAP1	<0.001	0.554082	0.503196	0.610114
IQSEC1	<0.001	0.379637	0.32158	0.448175
IQSEC3	1.84E-10	1.879842	1.548235	2.282475
IRAK1	5.53E-13	0.430658	0.342527	0.541463
IRAK3	<0.001	0.548215	0.491016	0.612078
IRAK4	<0.001	0.421891	0.364211	0.488705
IREB2	<0.001	0.407784	0.35375	0.470071
IRGC	3.45E-05	1.324463	1.159533	1.512853
ISCA1	<0.001	0.395455	0.333947	0.468291
ISCU	<0.001	0.205126	0.163386	0.25753
ISG20	0.003842	0.789852	0.673105	0.926849
ISYNA1	3.29E-06	0.529156	0.404687	0.691909
ITGA2B	0.130589	1.164044	0.955967	1.417411
ITGAL	<0.001	0.536195	0.470512	0.611047
ITGB1BP2	<0.001	0.476187	0.418812	0.541423
ITGB2	0.004567	0.780741	0.657992	0.92639
ITGB3BP	<0.001	0.560256	0.508957	0.616727
ITGB7	<0.001	0.44419	0.368776	0.535026
ITK	<0.001	0.567564	0.515461	0.624934
ITPA	7.41E-13	0.382892	0.294533	0.49776
ITPK1	0.417006	0.887364	0.664927	1.184211
ITPKA	5.46E-05	1.144444	1.071847	1.221957
ITPKB	<0.001	0.223284	0.172897	0.288356
ITPKC	4.77E-15	0.504641	0.425262	0.598837
ITPR1	<0.001	0.485077	0.429909	0.547325
ITPR2	<0.001	0.441433	0.388083	0.502117
ITPR3	<0.001	0.286726	0.225869	0.363981
IVD	<0.001	0.267327	0.211321	0.338177
JAK1	<0.001	0.452151	0.391154	0.52266
JAK2	<0.001	0.505567	0.454019	0.562968
JAK3	0.00677	1.437987	1.105541	1.870401
JMJD6	<0.001	0.251363	0.191088	0.330651
JUN	<0.001	0.516115	0.447426	0.595349
KALRN	<0.001	0.231236	0.180994	0.295425
KARS	<0.001	0.434916	0.3741	0.505618
KAT2A	<0.001	0.312244	0.253538	0.384544
KAT2B	<0.001	0.568156	0.520805	0.619813
KAT5	<0.001	0.194551	0.143514	0.263738

(*Continued*)

Table S1: *Continued*

Gene	*P* value	HR	lower 95% CI	upper 95% CI
KAT6A	<0.001	0.380396	0.321369	0.450266
KAT6B	<0.001	0.21935	0.173908	0.276666
KAT7	<0.001	0.244801	0.189971	0.315455
KAT8	<0.001	0.216313	0.172056	0.271954
KATNA1	<0.001	0.433366	0.379508	0.494869
KCNA1	0.01179	0.804986	0.679935	0.953037
KCNA10	9.30E-11	1.418464	1.276097	1.576714
KCNA3	<0.001	0.571585	0.513852	0.635803
KCNA4	0.091891	0.872359	0.744275	1.022485
KCNAB2	0.004294	1.425164	1.117539	1.817469
KCNC3	4.29E-08	1.652383	1.380635	1.977619
KCNC4	<0.001	0.371526	0.29699	0.464768
KCND2	2.61E-13	0.718647	0.65775	0.785182
KCND3	5.11E-05	0.648806	0.526267	0.799878
KCNE1	2.93E-08	0.668688	0.580029	0.770899
KCNE2	3.47E-12	1.461503	1.313327	1.626397
KCNH1	0.482809	0.957243	0.84727	1.081492
KCNH2	0.010928	0.811153	0.6904	0.953026
KCNH4	4.74E-13	1.76073	1.510467	2.052459
KCNH6	0.536306	1.06808	0.866857	1.316013
KCNIP1	2.91E-11	0.60999	0.527303	0.705643
KCNIP2	0.000351	1.448754	1.182251	1.775333
KCNJ1	0.065474	0.877534	0.763651	1.008401
KCNJ10	0.015571	1.209232	1.036697	1.410481
KCNJ13	6.63E-09	1.485211	1.299385	1.697613
KCNJ14	<0.001	0.236671	0.180519	0.310288
KCNJ15	<0.001	0.615445	0.56103	0.675137
KCNJ16	8.21E-10	0.680296	0.60159	0.7693
KCNJ2	<0.001	0.653704	0.59395	0.71947
KCNJ3	8.32E-12	0.465727	0.374057	0.579863
KCNJ4	1.35E-05	1.419122	1.212132	1.66146
KCNJ5	4.71E-07	0.536603	0.421187	0.683648
KCNJ6	0.292208	0.929737	0.811868	1.06472
KCNJ8	<0.001	0.526467	0.465786	0.595053
KCNJ9	5.65E-14	1.8851	1.597846	2.223994
KCNK1	9.21E-15	0.609563	0.537833	0.690859
KCNK3	1.70E-14	0.562751	0.485879	0.651785
KCNMA1	<0.001	0.354588	0.294196	0.427378
KCNMB1	<0.001	0.31554	0.25976	0.383298
KCNMB2	<0.001	0.378897	0.315728	0.454705

(*Continued*)

Table S1: *Continued*

Gene	*P* value	HR	lower 95% CI	upper 95% CI
KCNMB3	<0.001	0.468389	0.407165	0.538819
KCNMB4	3.63E-11	0.601227	0.517133	0.698996
KCNN1	0.036225	0.85902	0.745148	0.990294
KCNN2	<0.001	0.52485	0.467633	0.589068
KCNN3	5.23E-07	0.650881	0.550366	0.769754
KCNN4	0.865372	1.007965	0.91963	1.104786
KCNQ1	3.09E-05	1.411062	1.200075	1.659144
KCNQ2	6.10E-09	1.605536	1.368684	1.883375
KCNQ3	<0.001	0.43426	0.372815	0.505832
KCNQ4	0.004213	0.769965	0.643749	0.920927
KDM1A	<0.001	0.350954	0.282692	0.435699
KDM2A	<0.001	0.273726	0.221268	0.33862
KDM8	0.447205	0.911312	0.71724	1.157897
KDR	<0.001	0.562176	0.504821	0.626047
KDSR	<0.001	0.342658	0.289403	0.405713
KERA	<0.001	0.65216	0.593209	0.716968
KHK	6.60E-05	1.513141	1.234592	1.854537
KIF13A	<0.001	0.370937	0.31454	0.437447
KIF20A	0.143662	0.917333	0.817148	1.029801
KIFC1	1.11E-15	1.589433	1.419125	1.78018
KIT	<0.001	0.684941	0.638082	0.735241
KL	<0.001	0.55394	0.499047	0.61487
KLK6	0.381913	0.948839	0.843468	1.067374
KLKB1	<0.001	0.548057	0.492887	0.609402
KLRB1	<0.001	0.562635	0.509909	0.620813
KMO	<0.001	0.542547	0.487864	0.60336
KNG1	0.012557	1.263163	1.051458	1.517495
KPNA1	<0.001	0.436355	0.37825	0.503385
KPNA2	1.11E-16	0.566331	0.494734	0.64829
KPNA3	<0.001	0.473411	0.422802	0.530078
KPNA4	<0.001	0.452622	0.394276	0.519603
KPNA5	<0.001	0.520164	0.47103	0.574424
KPNA6	<0.001	0.321951	0.260749	0.397518
KPNB1	<0.001	0.363756	0.30575	0.432767
KRAS	<0.001	0.44737	0.393144	0.509076
KSR1	0.575236	1.069655	0.845196	1.353724
KYNU	2.17E-06	0.768419	0.689064	0.856912
L2HGDH	1.55E-15	0.410509	0.329813	0.510948
LAIR1	3.58E-05	0.706583	0.59929	0.833085
LALBA	0.009573	1.191667	1.043626	1.360708

(*Continued*)

Table S1: *Continued*

Gene	*P* value	HR	lower 95% CI	upper 95% CI
LAMA1	0.608668	0.963751	0.836716	1.110073
LAP3	<0.001	0.51488	0.460056	0.576236
LARS	<0.001	0.408232	0.355464	0.468834
LARS2	<0.001	0.329439	0.269988	0.401981
LAT	<0.001	0.458283	0.389075	0.539802
LATS1	<0.001	0.276293	0.222559	0.343
LBP	0.002196	1.14823	1.051015	1.254438
LCAT	0.974418	1.003675	0.802108	1.255895
LCE2B	<0.001	0.430563	0.365542	0.507149
LCK	<0.001	0.5346	0.464534	0.615235
LCMT1	<0.001	0.288104	0.231634	0.35834
LCMT2	<0.001	0.329911	0.275514	0.395048
LCT	<0.001	0.657036	0.598149	0.72172
LDHA	<0.001	0.384244	0.316848	0.465975
LDHAL6B	0.000526	0.822015	0.735803	0.918327
LDHB	<0.001	0.550892	0.497506	0.610006
LDHC	0.572779	1.030893	0.927453	1.14587
LDLR	0.000839	1.432915	1.160221	1.769703
LEP	0.088034	1.095234	0.986541	1.215903
LEPRE1	1.83E-08	0.515222	0.408968	0.64908
LEPREL1	0.023286	0.839021	0.720974	0.976396
LEPREL2	5.65E-05	1.317693	1.152132	1.507045
LGALS1	2.33E-13	1.904771	1.603234	2.263022
LGALS13	2.83E-09	0.657117	0.572118	0.754743
LGSN	1.37E-06	0.891145	0.850422	0.933818
LHCGR	3.61E-05	0.798426	0.717551	0.888416
LHPP	0.018099	0.762779	0.609369	0.95481
LIAS	4.61E-14	0.533106	0.452716	0.627771
LIG1	0.02425	0.765154	0.606211	0.965769
LIG3	<0.001	0.283843	0.22953	0.351008
LIG4	<0.001	0.549103	0.499622	0.603486
LILRB1	0.291487	1.083116	0.933787	1.256326
LILRB2	5.71E-12	0.618149	0.539062	0.70884
LILRB4	2.69E-12	0.496219	0.407738	0.6039
LIME1	0.000141	1.478911	1.209047	1.809009
LIMK1	1.33E-11	1.850387	1.548213	2.211537
LIMK2	<0.001	0.451555	0.384641	0.53011
LIN28A	<0.001	0.449818	0.384297	0.52651
LIN7A	<0.001	0.4103	0.343175	0.490555
LIN7B	<0.001	0.350118	0.283773	0.431975

(*Continued*)

Table S1: *Continued*

Gene	*P* value	HR	lower 95% CI	upper 95% CI
LIN7C	<0.001	0.552577	0.503753	0.606132
LIPA	<0.001	0.566555	0.510386	0.628906
LIPC	0.011279	1.172466	1.036698	1.326014
LIPE	8.29E-13	0.525778	0.440896	0.627002
LIPF	9.61E-05	0.779964	0.688398	0.883709
LIPG	1.18E-13	0.694149	0.630328	0.764431
LIPT1	<0.001	0.494515	0.445366	0.549087
LLGL2	<0.001	0.489339	0.41962	0.570641
LMAN2	0.003858	0.716441	0.571413	0.898279
LMBRD1	<0.001	0.438052	0.383588	0.500251
LMTK2	<0.001	0.276396	0.220639	0.346244
LNPEP	<0.001	0.353262	0.299069	0.417275
LOX	0.002013	0.796774	0.689793	0.920346
LOXL1	1.86E-10	0.64608	0.564853	0.738986
LOXL2	1.67E-15	2.033288	1.707527	2.421198
LPAR1	<0.001	0.457198	0.402073	0.51988
LPAR2	1.76E-07	0.551792	0.441437	0.689736
LPAR3	3.18E-05	0.781033	0.695184	0.877483
LPAR4	<0.001	0.488521	0.42906	0.556222
LPAR6	<0.001	0.439739	0.383006	0.504876
LPCAT1	<0.001	0.689646	0.63411	0.750045
LPCAT3	1.70E-10	0.534475	0.440992	0.647774
LPCAT4	9.17E-12	0.423404	0.33073	0.542047
LPGAT1	<0.001	0.548795	0.498372	0.604319
LPHN1	<0.001	0.321695	0.259507	0.398786
LPIN1	<0.001	0.37969	0.32323	0.446011
LPIN2	<0.001	0.551019	0.497207	0.610655
LPL	<0.001	0.680238	0.632313	0.731795
LPO	0.002442	1.187875	1.062705	1.327788
LPPR2	4.13E-05	0.515107	0.375125	0.707323
LPPR3	1.13E-07	1.467746	1.27373	1.691315
LPPR4	<0.001	0.55591	0.504443	0.612628
LRP1	0.156965	0.793199	0.575504	1.093242
LRP2	<0.001	0.658557	0.598941	0.724107
LRP5	5.02E-05	1.416609	1.197125	1.676333
LRRK1	<0.001	0.330388	0.265938	0.410458
LSG1	<0.001	0.502111	0.428554	0.588293
LSS	<0.001	0.313084	0.244346	0.401159
LTB	<0.001	0.350336	0.290651	0.422278
LTB4R	0.512174	0.943675	0.793479	1.122301

(*Continued*)

Table S1: *Continued*

Gene	*P* value	HR	lower 95% CI	upper 95% CI
LTB4R2	2.06E-05	1.270976	1.138165	1.419285
LTC4S	0.069303	1.173279	0.987453	1.394075
LTF	0.033158	0.945979	0.898855	0.995572
LUC7L3	<0.001	0.530003	0.480007	0.585206
LYN	<0.001	0.565072	0.499853	0.638801
LYPLA1	<0.001	0.497252	0.441032	0.560638
LYPLA2	<0.001	0.258546	0.20202	0.330887
LYVE1	9.85E-11	0.688199	0.614534	0.770693
LYZ	0.000786	0.845441	0.766514	0.932495
LYZL6	0.001186	0.767071	0.653458	0.900439
LZTS1	0.000103	1.652744	1.282593	2.129719
M6PR	6.55E-14	0.575346	0.497926	0.664805
MADD	<0.001	0.247347	0.196356	0.311581
MAFF	<0.001	0.515791	0.443263	0.600187
MAGT1	<0.001	0.389145	0.337957	0.448086
MAK	<0.001	0.431372	0.358047	0.519713
MAN1A1	<0.001	0.538143	0.487424	0.594139
MAN1A2	<0.001	0.404991	0.350714	0.467668
MAN1B1	<0.001	0.317567	0.255202	0.395172
MAN1C1	<0.001	0.540714	0.484172	0.603859
MAN2A1	<0.001	0.582107	0.534824	0.633571
MAN2A2	<0.001	0.334408	0.283184	0.394897
MAN2B1	<0.001	0.393391	0.327642	0.472334
MAN2B2	<0.001	0.436321	0.379083	0.502201
MAOA	<0.001	0.680546	0.631046	0.733929
MAOB	<0.001	0.402671	0.328691	0.493303
MAP2	<0.001	0.664106	0.606839	0.726778
MAP2K1	<0.001	0.472255	0.413277	0.539649
MAP2K2	<0.001	2.219652	1.861063	2.647335
MAP2K4	<0.001	0.436883	0.384939	0.495837
MAP2K5	<0.001	0.171872	0.119884	0.246405
MAP2K7	0.018808	0.700336	0.520296	0.942677
MAP3K1	<0.001	0.485733	0.430839	0.547621
MAP3K10	<0.001	1.447198	1.335575	1.568149
MAP3K11	1.18E-08	1.695247	1.413959	2.032493
MAP3K12	<0.001	0.292716	0.235771	0.363415
MAP3K13	<0.001	0.335303	0.280192	0.401254
MAP3K14	<0.001	0.367117	0.306051	0.440367
MAP3K2	<0.001	0.378022	0.323947	0.441122
MAP3K3	<0.001	0.319271	0.259877	0.39224

(*Continued*)

Table S1: *Continued*

Gene	*P* value	HR	lower 95% CI	upper 95% CI
MAP3K4	<0.001	0.410042	0.356046	0.472225
MAP3K5	<0.001	0.488998	0.435437	0.549148
MAP3K6	3.07E-12	0.40705	0.316194	0.524012
MAP3K7	<0.001	0.46136	0.407638	0.522161
MAP3K8	<0.001	0.557299	0.499775	0.621443
MAP3K9	<0.001	0.473701	0.399498	0.561686
MAP4K1	<0.001	0.460593	0.390869	0.542756
MAP4K2	0.208006	1.094768	0.950846	1.260473
MAP4K3	<0.001	0.560094	0.514051	0.610261
MAP4K4	<0.001	0.474145	0.41531	0.541313
MAP4K5	<0.001	0.565398	0.516254	0.619219
MAPK1	<0.001	0.283818	0.22003	0.366097
MAPK10	<0.001	0.349357	0.291912	0.418106
MAPK11	<0.001	1.799922	1.581954	2.047922
MAPK12	1.13E-08	1.710868	1.422873	2.057154
MAPK13	6.44E-13	0.538173	0.45455	0.637181
MAPK14	<0.001	0.365739	0.298532	0.448076
MAPK3	0.411784	0.867553	0.617941	1.217993
MAPK4	1.39E-08	1.551808	1.333271	1.806166
MAPK6	<0.001	0.456112	0.390912	0.532187
MAPK7	0.000852	0.650969	0.505828	0.837757
MAPK8	<0.001	0.331408	0.277033	0.396457
MAPK8IP1	0.050378	1.138345	0.999787	1.296106
MAPK9	<0.001	0.44536	0.391286	0.506908
MAPKAP1	<0.001	0.337765	0.277243	0.411499
MAPKAPK2	<0.001	0.282984	0.217932	0.367454
MAPKAPK3	<0.001	0.43297	0.364084	0.51489
MAPKAPK5	<0.001	0.399086	0.331407	0.480586
MARK1	<0.001	0.597751	0.533041	0.670317
MARK2	1.11E-07	1.797288	1.447454	2.231674
MARK3	<0.001	0.455034	0.399773	0.517934
MARK4	3.16E-07	1.792306	1.433128	2.241502
MASP2	0.839557	0.976367	0.774565	1.230745
MAST1	1.83E-08	1.389593	1.239147	1.558304
MAST2	<0.001	0.261967	0.198424	0.34586
MAST3	<0.001	0.378668	0.320064	0.448003
MAST4	<0.001	0.416457	0.353973	0.489972
MAT1A	1.77E-11	1.35002	1.236941	1.473437
MAT2A	<0.001	0.343347	0.286932	0.410855
MAT2B	<0.001	0.349373	0.278282	0.438626

(*Continued*)

Table S1: *Continued*

Gene	*P* value	HR	lower 95% CI	upper 95% CI
MB	9.78E-05	0.835686	0.763529	0.914663
MBD1	<0.001	0.263868	0.200881	0.346605
MBD2	<0.001	0.264011	0.208469	0.334352
MBL2	0.000534	1.232419	1.094956	1.387141
MBOAT2	<0.001	0.562897	0.505482	0.626834
MBOAT7	0.044442	0.749963	0.56648	0.992877
MBP	<0.001	0.259051	0.193122	0.347488
MBTPS1	<0.001	0.336577	0.283874	0.399064
MCAM	<0.001	0.522636	0.449192	0.608089
MCAT	<0.001	0.350953	0.279892	0.440055
MCCC1	<0.001	0.490246	0.434237	0.553479
MCCC2	<0.001	0.397658	0.341087	0.463612
MCF2	1.11E-16	0.369446	0.292071	0.467319
MCF2L	<0.001	0.247273	0.187125	0.326756
MCF2L2	3.87E-08	0.761686	0.691225	0.839329
MCFD2	<0.001	0.308599	0.255069	0.373363
MCM2	1.74E-09	0.721795	0.649117	0.802612
MCM3	0.340571	1.091932	0.911265	1.308417
MCM4	0.056029	1.17238	0.995918	1.380109
MCM5	3.73E-09	0.608045	0.515359	0.7174
MCM6	<0.001	0.487977	0.425552	0.55956
MCM7	1.36E-09	0.607272	0.516798	0.713585
MCM9	<0.001	0.360597	0.307791	0.422463
MCTP1	<0.001	0.546128	0.495382	0.602074
MCTP2	<0.001	0.555911	0.502767	0.614672
MDH1	<0.001	0.473558	0.421205	0.532417
MDH2	<0.001	0.363863	0.304496	0.434804
MDK	0.011623	1.159394	1.033574	1.300529
MDM2	<0.001	0.304781	0.245251	0.378761
ME1	<0.001	0.545463	0.486857	0.611123
ME2	<0.001	0.475759	0.418325	0.541078
ME3	<0.001	0.420393	0.346818	0.509576
MECR	0.00018	0.550956	0.403334	0.752608
MEIS1	<0.001	0.457048	0.40238	0.519144
MELK	2.32E-05	0.830093	0.761497	0.904868
MEPCE	<0.001	0.330341	0.267489	0.407961
MERTK	<0.001	0.353846	0.299656	0.417835
MET	<0.001	0.611797	0.557034	0.671944
METAP1	<0.001	0.468355	0.414634	0.529036
METAP2	<0.001	0.456396	0.405137	0.51414

(*Continued*)

Table S1: *Continued*

Gene	*P* value	HR	lower 95% CI	upper 95% CI
METTL1	5.72E-14	0.416292	0.331228	0.523202
METTL2B	<0.001	0.538788	0.4789	0.606167
METTL3	<0.001	0.41111	0.358384	0.471593
MFAP4	<0.001	0.703907	0.654606	0.756922
MFGE8	1.75E-05	1.503146	1.248052	1.81038
MFI2	0.200706	0.88961	0.743679	1.064177
MFN1	<0.001	0.563197	0.507458	0.625058
MFN2	1.25E-08	0.378182	0.270596	0.528544
MGAM	2.45E-10	0.692864	0.618456	0.776223
MGAT1	<0.001	0.410935	0.342871	0.492509
MGAT2	7.14E-13	0.445144	0.356867	0.555258
MGAT3	0.762325	0.969553	0.793498	1.18467
MGAT4A	<0.001	0.503754	0.451841	0.561631
MGAT4B	7.11E-15	0.462614	0.380978	0.561743
MGAT4C	2.82E-10	0.593745	0.504963	0.698137
MGAT5	<0.001	0.328355	0.262948	0.410032
MGEA5	<0.001	0.407509	0.350318	0.474036
MGLL	<0.001	0.635329	0.576184	0.700545
MGMT	0.423514	0.90932	0.720473	1.147667
MGRN1	0.000258	0.629569	0.491213	0.806896
MGST2	<0.001	0.474103	0.414635	0.5421
MGST3	1.03E-10	0.346223	0.250985	0.477598
MICAL1	<0.001	0.451882	0.386613	0.528169
MICAL2	<0.001	0.516079	0.446687	0.596252
MICAL3	<0.001	0.27774	0.218405	0.353194
MID1	<0.001	0.512578	0.458887	0.57255
MIF	<0.001	1.968945	1.697972	2.283161
MINA	<0.001	0.427466	0.371687	0.491616
MINK1	6.64E-12	1.735872	1.482984	2.031885
MINPP1	<0.001	0.52566	0.474876	0.581875
MIPEP	<0.001	0.451392	0.393685	0.517558
MKNK1	<0.001	0.336613	0.276651	0.40957
MKNK2	<0.001	0.412242	0.349637	0.486058
MLPH	<0.001	0.721101	0.676176	0.76901
MLYCD	<0.001	0.212592	0.167731	0.269451
MMACHC	<0.001	0.355834	0.293059	0.432056
MME	1.62E-09	0.756447	0.690856	0.828264
MMP1	0.544777	0.984562	0.936213	1.035407
MMP10	0.055275	0.941635	0.885473	1.001358
MMP11	1.83E-09	1.366864	1.234457	1.513472

(*Continued*)

Table S1: *Continued*

Gene	*P* value	HR	lower 95% CI	upper 95% CI
MMP12	0.058054	1.059178	0.99804	1.124061
MMP13	0.001402	0.913749	0.864553	0.965745
MMP14	<0.001	1.920504	1.709335	2.15776
MMP15	6.58E-05	1.402386	1.187798	1.65574
MMP16	<0.001	0.276513	0.221884	0.344591
MMP17	1.78E-06	1.404873	1.221967	1.615156
MMP19	4.53E-08	0.576546	0.473279	0.702344
MMP2	5.46E-05	0.598159	0.466025	0.767757
MMP20	1.76E-08	1.410476	1.251413	1.589758
MMP24	0.004913	0.80271	0.688734	0.935547
MMP25	<0.001	0.272659	0.215205	0.345451
MMP26	9.77E-15	0.612743	0.541286	0.693634
MMP27	<0.001	0.333421	0.275076	0.404141
MMP28	8.77E-14	0.666551	0.599153	0.74153
MMP3	0.008475	0.902704	0.836466	0.974187
MMP7	<0.001	0.754198	0.706081	0.805594
MMP8	1.27E-07	0.673985	0.582203	0.780237
MMP9	0.048899	0.916284	0.839931	0.999578
MOCOS	9.08E-13	0.58301	0.502791	0.676028
MOCS1	<0.001	0.288045	0.233897	0.354728
MOCS2	<0.001	0.41193	0.359624	0.471843
MOCS3	1.15E-09	0.486586	0.385854	0.613615
MOGAT2	0.017352	0.817576	0.692578	0.965134
MOGS	2.22E-16	0.322241	0.24589	0.4223
MOS	2.04E-08	0.706406	0.62561	0.797636
MOXD1	<0.001	0.523344	0.466113	0.587602
MPC1	<0.001	0.379174	0.320722	0.448279
MPC2	<0.001	0.377308	0.325881	0.43685
MPG	3.33E-14	0.353368	0.270077	0.462345
MPI	<0.001	0.313111	0.253568	0.386637
MPO	<0.001	0.296074	0.232336	0.377298
MPST	<0.001	0.314115	0.256548	0.3846
MRC2	0.004206	0.71631	0.570008	0.900163
MRE11A	<0.001	0.451819	0.396531	0.514816
MRS2	<0.001	0.381342	0.326602	0.445256
MSMO1	<0.001	0.513208	0.45668	0.576734
MSRA	<0.001	0.314034	0.2545	0.387493
MSRB1	0.000126	0.720752	0.609654	0.852096
MSRB2	<0.001	0.435642	0.375793	0.505023
MST1R	<0.001	0.535957	0.476366	0.603003

(*Continued*)

Table S1: *Continued*

Gene	*P* value	HR	lower 95% CI	upper 95% CI
MST4	<0.001	0.62325	0.563192	0.689713
MT1E	0.040726	0.845041	0.719185	0.99292
MT1F	0.050215	0.857019	0.734375	1.000145
MT1G	4.74E-08	1.719562	1.41554	2.08888
MT1H	0.714444	1.030042	0.879015	1.207017
MT1M	0.016889	0.887322	0.804431	0.978754
MT1X	3.63E-06	1.381829	1.205081	1.584499
MT3	3.35E-07	0.65649	0.5585	0.771672
MT4	3.03E-14	1.600118	1.417366	1.806434
MTAP	<0.001	0.407351	0.343269	0.483395
MTG1	<0.001	0.426104	0.35658	0.509183
MTHFD1	3.88E-13	0.470358	0.383699	0.576589
MTHFD2	0.029286	0.861484	0.75339	0.985087
MTHFD2L	<0.001	0.348388	0.295616	0.410581
MTHFR	2.75E-11	0.391856	0.297425	0.516268
MTIF2	<0.001	0.551636	0.502772	0.60525
MTM1	<0.001	0.512322	0.462321	0.567731
MTMR1	<0.001	0.381336	0.322536	0.450856
MTMR14	<0.001	0.209172	0.157753	0.27735
MTMR2	<0.001	0.474286	0.414913	0.542155
MTMR3	6.66E-16	0.322596	0.245122	0.424556
MTMR4	<0.001	0.436653	0.380612	0.500944
MTMR6	<0.001	0.527348	0.47728	0.58267
MTMR7	0.002	0.755581	0.632533	0.902567
MTNR1A	2.08E-05	1.346019	1.173879	1.543403
MTNR1B	2.50E-11	1.484738	1.322019	1.667486
MTO1	<0.001	0.418457	0.366516	0.477759
MTOR	9.26E-09	0.55861	0.457947	0.6814
MTPAP	<0.001	0.475715	0.421648	0.536715
MTR	<0.001	0.488683	0.439202	0.54374
MTRR	<0.001	0.48111	0.429241	0.539247
MTTP	4.63E-06	0.764903	0.682036	0.857838
MUS81	<0.001	0.327385	0.268739	0.39883
MUSK	0.046816	0.81852	0.671867	0.997184
MUT	<0.001	0.500449	0.452205	0.55384
MVD	1.60E-12	1.515875	1.350658	1.701301
MVK	0.000479	1.53537	1.207009	1.953061
MYBBP1A	1.95E-11	1.7896	1.509824	2.12122
MYCBP2	<0.001	0.416781	0.364071	0.477124
MYL1	0.398385	0.943404	0.82409	1.079991

(*Continued*)

Table S1: *Continued*

Gene	*P* value	HR	lower 95% CI	upper 95% CI
MYL10	1.79E-05	0.641158	0.523317	0.785533
MYL12A	<0.001	0.379229	0.32423	0.443558
MYL12B	<0.001	0.376623	0.323318	0.438716
MYL2	0.000673	1.276193	1.108837	1.468807
MYL3	7.21E-09	1.403523	1.251266	1.574308
MYL4	<0.001	0.210931	0.158598	0.280532
MYL5	0.714436	1.03848	0.848323	1.271261
MYL6	<0.001	0.189999	0.143879	0.250903
MYL6B	0.16559	1.186311	0.931777	1.510377
MYL7	<0.001	1.535164	1.39278	1.692104
MYL9	1.40E-14	0.458769	0.3762	0.559462
MYLIP	<0.001	0.366612	0.312832	0.429638
MYLK	<0.001	0.35126	0.293155	0.420882
MYLK3	1.11E-16	0.300944	0.226607	0.399665
MYLPF	2.01E-13	1.9519	1.632991	2.33309
MYO3A	0.472074	0.932278	0.770088	1.128628
MYO6	<0.001	0.583116	0.534995	0.635564
MYO9A	<0.001	0.464546	0.411072	0.524975
MYO9B	1.24E-12	0.339382	0.251851	0.457334
MYRIP	<0.001	0.449127	0.388341	0.519428
NAA10	1.02E-14	0.348092	0.266434	0.454776
NAA11	1.16E-10	0.647366	0.567166	0.738907
NAA60	<0.001	0.213541	0.165107	0.276183
NAALADL1	1.55E-06	0.564933	0.447537	0.713123
NADK	<0.001	0.265545	0.203054	0.347267
NADSYN1	<0.001	0.257054	0.201919	0.327243
NAGA	<0.001	0.425319	0.359351	0.503396
NAGK	<0.001	0.378016	0.301535	0.473897
NAGLU	0.661557	1.056438	0.826207	1.350827
NAGPA	<0.001	0.377761	0.316133	0.451403
NAMPT	<0.001	0.647695	0.590393	0.710558
NANS	<0.001	0.260169	0.206376	0.327984
NARS	<0.001	0.454716	0.402148	0.514156
NARS2	<0.001	0.459478	0.406082	0.519896
NAT1	<0.001	0.558871	0.511203	0.610983
NAT2	0.000908	1.204097	1.078964	1.343743
NAT6	0.405521	1.082999	0.8975	1.306837
NAV2	<0.001	0.234643	0.182703	0.301349
NCALD	<0.001	0.310468	0.255655	0.377034
NCAN	6.35E-10	0.625794	0.539368	0.726067

(*Continued*)

Table S1: *Continued*

Gene	*P* value	HR	lower 95% CI	upper 95% CI
NCF1	0.101503	0.878562	0.752448	1.025813
NCF2	<0.001	0.683785	0.629807	0.742389
NCF4	<0.001	0.510095	0.437598	0.594601
NCOA1	<0.001	0.350331	0.299962	0.409158
NCOA3	<0.001	0.436773	0.376003	0.507365
NCOA6	<0.001	0.42404	0.364424	0.493408
NCOR2	3.73E-06	1.657527	1.338056	2.053275
NCS1	1.58E-09	1.903519	1.544502	2.345988
NDOR1	2.78E-15	1.865436	1.598054	2.177556
NDRG1	0.001384	0.792195	0.686798	0.913767
NDST1	9.39E-06	0.477401	0.344218	0.662115
NDST2	0.010415	0.769568	0.629819	0.940326
NDST3	2.92E-05	0.750644	0.656181	0.858705
NDST4	0.049346	1.168777	1.000446	1.365431
NDUFA1	<0.001	0.396308	0.319532	0.491531
NDUFA10	<0.001	0.247726	0.185516	0.330797
NDUFA13	0.089728	0.776842	0.580309	1.039935
NDUFA2	0.081837	0.791102	0.607586	1.030047
NDUFA3	8.10E-06	1.915242	1.439691	2.547875
NDUFA4	<0.001	0.259858	0.210545	0.320721
NDUFA4L2	1.60E-08	1.240532	1.151168	1.336834
NDUFA5	<0.001	0.449073	0.39652	0.50859
NDUFA6	<0.001	0.354221	0.300534	0.417497
NDUFA7	1.13E-09	1.605925	1.378855	1.870388
NDUFA8	<0.001	0.386116	0.321512	0.463701
NDUFA9	<0.001	0.419355	0.353369	0.497663
NDUFAB1	<0.001	0.360229	0.284416	0.456251
NDUFAF1	<0.001	0.419044	0.366508	0.479111
NDUFAF4	<0.001	0.454166	0.401126	0.51422
NDUFAF5	6.58E-06	1.266894	1.143037	1.404173
NDUFB1	<0.001	0.235984	0.185997	0.299406
NDUFB11	0.160757	0.785065	0.559811	1.100956
NDUFB2	<0.001	0.239873	0.188418	0.30538
NDUFB3	<0.001	0.41673	0.354704	0.489603
NDUFB4	<0.001	0.353455	0.295216	0.423183
NDUFB5	<0.001	0.472507	0.416088	0.536576
NDUFB6	<0.001	0.368057	0.311721	0.434574
NDUFB7	9.38E-08	0.38886	0.274908	0.550045
NDUFB8	<0.001	0.287752	0.235274	0.351934
NDUFC1	<0.001	0.267144	0.208848	0.341713

(*Continued*)

Table S1: *Continued*

Gene	*P* value	HR	lower 95% CI	upper 95% CI
NDUFS1	<0.001	0.40238	0.343217	0.471741
NDUFS2	<0.001	0.356577	0.2964	0.42897
NDUFS3	<0.001	0.269056	0.210908	0.343237
NDUFS4	<0.001	0.354188	0.302093	0.415266
NDUFS6	<0.001	0.43106	0.370952	0.500909
NDUFS7	9.99E-11	0.374291	0.277883	0.504146
NDUFV1	0.010791	1.402874	1.081413	1.819892
NDUFV2	2.69E-07	0.557018	0.445697	0.696143
NEDD4	<0.001	0.592361	0.543909	0.645128
NEDD4L	<0.001	0.496069	0.44006	0.559206
NEDD9	<0.001	0.442811	0.382722	0.512333
NEK1	<0.001	0.470769	0.41339	0.536113
NEK11	<0.001	0.330527	0.272907	0.400312
NEK2	0.22444	0.940861	0.852731	1.0381
NEK3	<0.001	0.40656	0.349435	0.473025
NEK4	<0.001	0.369865	0.314257	0.435312
NEK7	<0.001	0.517523	0.470201	0.569607
NEK9	0.980512	0.996843	0.773435	1.284781
NENF	5.59E-08	2.252	1.680141	3.018499
NET1	<0.001	0.561402	0.507618	0.620883
NEU1	6.86E-13	0.468417	0.380847	0.576123
NEU2	0.002643	1.238766	1.077378	1.424331
NEU3	4.27E-09	0.495855	0.392369	0.626636
NFASC	<0.001	0.380154	0.309772	0.466528
NFATC1	0.072112	0.76364	0.569189	1.02452
NFATC3	<0.001	0.330399	0.271236	0.402468
NFATC4	3.55E-05	0.549745	0.414003	0.729993
NFE2	1.45E-06	0.740573	0.655411	0.836801
NFE2L1	<0.001	0.339173	0.27433	0.419343
NFKB1	<0.001	0.405932	0.346897	0.475013
NFS1	<0.001	0.366234	0.297363	0.451057
NFU1	<0.001	0.434463	0.383188	0.492598
NGB	2.07E-05	1.389986	1.19446	1.617518
NGF	0.028014	0.848309	0.73252	0.982401
NGLY1	<0.001	0.462286	0.408818	0.522747
NIN	<0.001	0.376164	0.321036	0.440758
NIPA2	<0.001	0.389022	0.332735	0.454832
NIT2	<0.001	0.444533	0.381	0.51866
NKIRAS2	<0.001	0.370502	0.308016	0.445665
NLK	<0.001	0.445104	0.390104	0.507858

(*Continued*)

Table S1: *Continued*

Gene	*P* value	HR	lower 95% CI	upper 95% CI
NME1	0.074385	1.175464	0.984205	1.403891
NME3	<0.001	0.380326	0.313023	0.462099
NME4	0.183045	0.86304	0.694807	1.072007
NME5	<0.001	0.602537	0.554787	0.654396
NME6	2.22E-16	0.312735	0.237006	0.412662
NME7	<0.001	0.572282	0.524213	0.624759
NMNAT2	1.82E-06	0.700357	0.60506	0.810664
NMRK1	<0.001	0.416737	0.363876	0.477277
NMRK2	0.672638	0.968622	0.835469	1.122997
NMT1	<0.001	0.253816	0.201838	0.319179
NMT2	<0.001	0.50849	0.451904	0.572162
NNT	<0.001	0.388629	0.335861	0.449687
NOL8	<0.001	0.551597	0.499625	0.608975
NOLC1	<0.001	0.421504	0.35632	0.498613
NONO	<0.001	0.357804	0.294582	0.434595
NOS1	0.311843	1.101226	0.913529	1.327489
NOS2	1.07E-07	1.382769	1.22697	1.558353
NOS3	5.07E-07	1.472481	1.266144	1.712444
NOTCH1	0.297389	1.119127	0.905614	1.382979
NOX1	2.13E-07	0.530823	0.417868	0.674312
NOX3	1.79E-10	1.602447	1.386295	1.852302
NOX4	<0.001	0.55133	0.495095	0.613952
NOX5	0.00081	1.441563	1.163816	1.785594
NPC1L1	4.93E-08	1.442797	1.264714	1.645956
NPC2	<0.001	0.526083	0.461948	0.599123
NPEPL1	<0.001	0.294817	0.241605	0.359748
NPFFR1	0.26329	1.132747	0.910516	1.409218
NPL	<0.001	0.523051	0.460842	0.593658
NPPB	6.99E-05	1.366263	1.171473	1.593443
NPR1	<0.001	0.29723	0.232674	0.379698
NPR2	<0.001	0.380802	0.321051	0.451675
NPR3	<0.001	0.586065	0.527591	0.651021
NPRL2	<0.001	0.345672	0.275742	0.433336
NPRL3	3.33E-16	0.222	0.154769	0.318436
NPTX1	1.19E-09	0.693667	0.616541	0.78044
NPTX2	0.013875	0.897331	0.823144	0.978204
NPTXR	0.272466	1.080384	0.941038	1.240364
NPVF	0.637223	1.028901	0.914003	1.158241
NQO1	1.93E-05	0.839515	0.774778	0.909661
NQO2	5.20E-14	0.460065	0.375854	0.563144

(*Continued*)

Table S1: *Continued*

Gene	*P* value	HR	lower 95% CI	upper 95% CI
NR0B1	0.43702	1.040598	0.94124	1.150445
NR1H4	0.054652	0.834905	0.694561	1.003606
NR1I2	2.49E-07	1.352868	1.206097	1.5175
NR1I3	0.348427	0.917753	0.767028	1.098096
NR3C2	<0.001	0.641278	0.595255	0.690858
NR5A1	0.263398	1.085403	0.940196	1.253037
NRAS	<0.001	0.552155	0.493198	0.61816
NRF1	<0.001	0.186315	0.140453	0.247152
NRGN	0.794315	0.982024	0.856836	1.125503
NSD1	<0.001	0.314872	0.25558	0.387919
NSDHL	<0.001	0.439636	0.366824	0.526902
NSMAF	<0.001	0.32062	0.266688	0.385458
NSUN3	<0.001	0.373352	0.314599	0.443078
NSUN5	<0.001	0.377592	0.317911	0.448476
NSUN6	<0.001	0.449208	0.39205	0.514699
NSUN7	<0.001	0.443942	0.389292	0.506262
NT5C	2.17E-05	1.37721	1.188065	1.596469
NT5C2	<0.001	0.35359	0.297494	0.420264
NT5DC3	<0.001	0.288789	0.230919	0.361161
NT5E	<0.001	0.615879	0.562663	0.674128
NT5M	0.635528	1.044549	0.872273	1.25085
NTRK1	0.043666	1.151562	1.004018	1.320789
NTRK2	0.000594	0.76106	0.651241	0.889398
NTRK3	<0.001	0.275892	0.212495	0.358203
NTSR1	8.23E-09	1.481438	1.296113	1.693262
NTSR2	1.11E-16	1.572547	1.413448	1.749554
NUAK1	2.22E-16	0.633584	0.568102	0.706615
NUCB1	9.78E-06	0.686	0.580468	0.810717
NUCB2	<0.001	0.47906	0.427648	0.536652
NUDT1	0.112225	1.145026	0.968804	1.353303
NUDT11	4.00E-13	0.638035	0.565102	0.72038
NUDT15	<0.001	0.520429	0.455408	0.594734
NUDT18	<0.001	0.287733	0.231598	0.357473
NUDT2	<0.001	0.464578	0.392647	0.549687
NUDT3	<0.001	0.278522	0.226095	0.343104
NUDT4	4.85E-06	0.580701	0.459985	0.733097
NUDT7	<0.001	0.459539	0.388209	0.543975
NUDT9	<0.001	0.485123	0.43363	0.54273
NXN	2.55E-15	0.636184	0.568752	0.711612
NXT1	2.26E-10	0.460515	0.362396	0.5852

(*Continued*)

Table S1: *Continued*

Gene	*P* value	HR	lower 95% CI	upper 95% CI
OAS3	0.001212	0.765922	0.651685	0.900182
OAT	<0.001	0.5	0.448492	0.557424
OAZ1	<0.001	0.194687	0.13535	0.280038
OAZ2	<0.001	0.249943	0.190076	0.328667
OAZ3	0.379666	1.091448	0.897879	1.326748
OBSCN	0.000148	0.653806	0.524979	0.814248
OCA2	<0.001	0.494811	0.425645	0.575217
OCRL	<0.001	0.340644	0.282745	0.410398
ODC1	<0.001	0.573615	0.511733	0.642981
OGDH	0.000871	0.643177	0.496009	0.83401
OGDHL	2.93E-11	1.285235	1.193606	1.383899
OGFOD1	<0.001	0.273962	0.215378	0.34848
OGFOD2	<0.001	0.173817	0.130583	0.231365
OGT	<0.001	0.40722	0.354873	0.467287
OLA1	<0.001	0.445907	0.392658	0.506378
OLAH	1.14E-12	0.488891	0.401391	0.595465
OMD	<0.001	0.581733	0.527316	0.641767
OPA1	<0.001	0.53602	0.485534	0.591755
OPHN1	<0.001	0.188757	0.139359	0.255665
OPLAH	7.65E-13	0.562734	0.480863	0.658543
OPRD1	0.103689	1.136346	0.974202	1.325478
OPRK1	1.88E-11	0.634764	0.555906	0.72481
OPRM1	0.026588	0.784455	0.632967	0.972199
OSBP	<0.001	0.367172	0.310314	0.434448
OSBPL1A	<0.001	0.337332	0.277287	0.41038
OSBPL2	<0.001	0.396754	0.341203	0.461349
OTC	0.000177	1.304598	1.135326	1.499109
OXCT1	<0.001	0.558043	0.507843	0.613205
OXCT2	<0.001	0.266378	0.20593	0.34457
OXSM	<0.001	0.373035	0.320109	0.434711
OXSR1	<0.001	0.387514	0.324485	0.462786
OXT	6.68E-06	1.36822	1.193717	1.568233
OXTR	<0.001	0.497249	0.437235	0.5655
P2RX1	0.000117	0.761414	0.662828	0.874664
P2RY1	<0.001	0.607916	0.554342	0.666666
P2RY2	0.035312	0.829423	0.69685	0.987216
P2RY4	<0.001	0.412901	0.353183	0.482716
P2RY6	0.000192	1.237539	1.106392	1.384232
P4HA1	<0.001	0.435369	0.374274	0.506437
P4HB	0.021337	0.768075	0.613525	0.961555

(*Continued*)

Table S1: *Continued*

Gene	*P* value	HR	lower 95% CI	upper 95% CI
P4HTM	<0.001	0.496339	0.44206	0.557282
PABPN1	<0.001	0.397379	0.337114	0.468418
PACS1	<0.001	0.303492	0.248685	0.370378
PADI1	2.96E-05	0.684505	0.572961	0.817765
PADI2	2.22E-16	0.377883	0.29944	0.476876
PADI3	2.70E-07	1.241439	1.143224	1.348091
PADI4	0.002289	1.338856	1.109916	1.615019
PAEP	3.81E-07	1.106327	1.064013	1.150325
PAFAH1B1	<0.001	0.378245	0.325352	0.439736
PAFAH1B2	<0.001	0.273469	0.207308	0.360747
PAFAH1B3	3.38E-10	0.585151	0.495037	0.691669
PAFAH2	5.40E-12	0.435766	0.344114	0.55183
PAICS	<0.001	0.506517	0.435627	0.588944
PAK1	<0.001	0.373095	0.312822	0.444981
PAK2	<0.001	0.449856	0.376471	0.537545
PAK3	<0.001	0.518129	0.456446	0.588148
PAK4	9.49E-07	0.541773	0.42402	0.692226
PAK6	<0.001	0.544516	0.474622	0.624702
PAK7	0.489381	1.036372	0.936538	1.146847
PAM	<0.001	0.544167	0.493048	0.600585
PANK2	<0.001	0.296853	0.24207	0.364033
PANK3	<0.001	0.479118	0.427042	0.537544
PANK4	<0.001	0.273989	0.218005	0.34435
PAOX	<0.001	0.212768	0.16059	0.281901
PAPD7	<0.001	0.473761	0.417547	0.537544
PAPOLA	<0.001	0.494029	0.441886	0.552324
PAPOLB	3.98E-06	0.654227	0.546288	0.783493
PAPOLG	<0.001	0.347796	0.29373	0.411814
PAPSS1	<0.001	0.380131	0.323787	0.446279
PAPSS2	<0.001	0.586082	0.526931	0.651872
PARD3	<0.001	0.419276	0.355476	0.494527
PARD6A	<0.001	0.279367	0.21647	0.36054
PARD6B	<0.001	0.377163	0.318859	0.446129
PARG	<0.001	0.385104	0.329711	0.449803
PARK2	<0.001	0.202905	0.148508	0.277229
PARP1	<0.001	0.3689	0.308736	0.44079
PARP11	<0.001	0.360804	0.306834	0.424266
PARP12	<0.001	0.584374	0.522067	0.654117
PARP16	<0.001	0.425715	0.368474	0.491848
PARP2	<0.001	0.405992	0.348274	0.473274

(*Continued*)

Table S1: *Continued*

Gene	*P* value	HR	lower 95% CI	upper 95% CI
PARP3	<0.001	0.396305	0.335558	0.468048
PARP4	<0.001	0.471921	0.418711	0.531894
PARP6	<0.001	0.365838	0.308049	0.434469
PARP8	<0.001	0.57949	0.5319	0.631338
PASK	<0.001	0.298323	0.245946	0.361854
PBK	0.001594	0.886565	0.822711	0.955376
PBX1	<0.001	0.564713	0.497766	0.640664
PC	0.004715	0.798095	0.682531	0.933226
PCBD1	3.75E-14	0.51422	0.43287	0.61086
PCCA	<0.001	0.393097	0.33435	0.462166
PCCB	1.30E-14	0.442449	0.359573	0.544427
PCDH11X	6.64E-10	0.672513	0.592935	0.76277
PCDH12	<0.001	0.352891	0.286608	0.434503
PCDH17	<0.001	0.604127	0.555176	0.657393
PCDH8	0.16359	0.951906	0.888132	1.020259
PCDH9	<0.001	0.451143	0.39066	0.520991
PCDHA10	0.006163	1.207968	1.055208	1.382844
PCDHA2	<0.001	1.787197	1.560447	2.046897
PCDHA5	<0.001	0.465681	0.390808	0.554899
PCDHA9	<0.001	0.43902	0.380307	0.506796
PCDHB1	2.32E-08	0.633796	0.540083	0.743769
PCDHB11	2.51E-10	1.479008	1.310122	1.669665
PCDHB12	3.13E-14	0.58097	0.504979	0.668395
PCDHB13	<0.001	0.368672	0.308518	0.440554
PCDHB3	2.11E-15	0.566813	0.492641	0.652151
PCDHB6	<0.001	0.529946	0.459399	0.611325
PCDHB8	1.11E-16	0.601849	0.533634	0.678784
PCDHGA1	<0.001	0.426767	0.362737	0.502098
PCDHGA10	2.58E-06	1.281448	1.155593	1.421009
PCDHGA3	0.163651	0.891517	0.758502	1.047859
PCDHGA8	<0.001	0.442519	0.372307	0.525971
PCDHGA9	5.55E-14	1.589489	1.408609	1.793597
PCDHGB5	<0.001	0.256201	0.195622	0.335539
PCDHGB6	0.022362	1.146949	1.019651	1.290141
PCDHGC3	1.92E-14	0.450695	0.367514	0.552704
PCK1	4.44E-06	0.765042	0.682362	0.857739
PCK2	<0.001	0.436036	0.372366	0.510592
PCLO	<0.001	0.475206	0.415212	0.543868
PCMT1	<0.001	0.412159	0.356751	0.476174
PCSK1	0.757585	0.990505	0.932279	1.052367

(*Continued*)

Table S1: *Continued*

Gene	*P* value	HR	lower 95% CI	upper 95% CI
PCSK6	0.178412	1.178059	0.927923	1.495622
PCSK7	<0.001	0.272524	0.215229	0.34507
PCTP	<0.001	0.571257	0.520404	0.62708
PCYOX1	<0.001	0.502683	0.451087	0.56018
PCYOX1L	<0.001	0.414374	0.350897	0.489333
PCYT1A	<0.001	0.449671	0.385527	0.524489
PCYT1B	2.78E-10	0.52822	0.433239	0.644024
PCYT2	0.004618	1.362409	1.099951	1.687492
PDC	3.10E-10	0.67384	0.595891	0.761987
PDCD6	<0.001	0.373282	0.308278	0.451993
PDCD6IP	<0.001	0.46057	0.408223	0.519631
PDCL	<0.001	0.34243	0.289281	0.405343
PDE10A	<0.001	0.413624	0.3504	0.488257
PDE11A	<0.001	0.260229	0.201856	0.335482
PDE1A	<0.001	0.414869	0.356616	0.482637
PDE1B	2.10E-13	1.466392	1.323951	1.624158
PDE1C	<0.001	0.26126	0.202903	0.336402
PDE2A	<0.001	0.474343	0.404349	0.556453
PDE3A	<0.001	0.439571	0.370927	0.520919
PDE3B	<0.001	0.448898	0.395782	0.509142
PDE4A	0.03256	1.304797	1.02234	1.665294
PDE4B	<0.001	0.459286	0.402261	0.524395
PDE4D	<0.001	0.456685	0.395726	0.527035
PDE4DIP	<0.001	0.431875	0.370904	0.502869
PDE5A	<0.001	0.379983	0.324183	0.445389
PDE6A	0.002244	1.191184	1.064753	1.332627
PDE6B	<0.001	0.381536	0.310526	0.468784
PDE6C	1.54E-11	1.474329	1.317037	1.650407
PDE6D	<0.001	0.264534	0.207666	0.336975
PDE6G	7.35E-14	1.597281	1.412865	1.80577
PDE6H	0.528035	1.044695	0.912024	1.196666
PDE7B	<0.001	0.363983	0.296466	0.446876
PDE8A	<0.001	0.310004	0.257747	0.372855
PDE8B	<0.001	0.368596	0.310921	0.436969
PDE9A	<0.001	0.505619	0.436165	0.586133
PDGFRA	<0.001	0.331585	0.271895	0.404378
PDGFRB	<0.001	0.468944	0.39492	0.556842
PDHA1	<0.001	0.395047	0.335614	0.465005
PDHA2	2.08E-12	0.584857	0.503613	0.679207
PDHB	<0.001	0.420457	0.362972	0.487046

(*Continued*)

Table S1: *Continued*

Gene	*P* value	HR	lower 95% CI	upper 95% CI
PDHX	<0.001	0.493272	0.442966	0.549292
PDK1	5.90E-08	0.621335	0.523135	0.737969
PDK3	<0.001	0.438979	0.381472	0.505156
PDK4	<0.001	0.535469	0.47657	0.601648
PDP1	<0.001	0.565474	0.505071	0.633101
PDPK1	8.85E-07	0.686526	0.590921	0.797598
PDPR	<0.001	0.318676	0.259589	0.391211
PDSS1	<0.001	0.38046	0.324079	0.446649
PDSS2	<0.001	0.487499	0.433912	0.547704
PDXK	<0.001	0.285142	0.219946	0.369662
PDZD8	<0.001	0.517463	0.464722	0.576189
PDZK1	<0.001	0.537779	0.470585	0.614567
PEA15	3.33E-16	0.433204	0.354513	0.529363
PEBP1	<0.001	0.307974	0.248616	0.381502
PECR	<0.001	0.35327	0.287853	0.433555
PEMT	3.29E-09	0.482651	0.379163	0.614383
PEPD	<0.001	0.477446	0.411449	0.554029
PET112	<0.001	0.331741	0.262675	0.418966
PFAS	<0.001	0.382305	0.306705	0.476539
PFKFB1	0.018209	0.746921	0.58626	0.951611
PFKFB2	<0.001	0.290184	0.233817	0.360138
PFKFB3	<0.001	0.558942	0.501977	0.622371
PFKFB4	0.107293	1.174287	0.965736	1.427873
PFKL	<0.001	0.336842	0.269448	0.421093
PFKM	<0.001	0.466406	0.407087	0.534369
PFKP	4.42E-10	0.542447	0.447609	0.657379
PGAM1	<0.001	0.421479	0.353891	0.501975
PGAM2	3.12E-06	1.310419	1.169645	1.468136
PGD	0.007326	0.718944	0.56488	0.915026
PGGT1B	<0.001	0.522355	0.470208	0.580285
PGK1	1.20E-08	0.576588	0.477128	0.696782
PGK2	1.14E-06	0.686296	0.589751	0.798646
PGLS	<0.001	0.237325	0.181759	0.30988
PGLYRP1	1.35E-08	1.373481	1.231	1.532453
PGLYRP4	1.99E-06	0.675775	0.574956	0.794272
PGM1	<0.001	0.567597	0.509042	0.632888
PGM3	<0.001	0.523974	0.473336	0.58003
PGM5	3.33E-16	0.425395	0.346583	0.52213
PGP	0.078107	1.232913	0.976722	1.556302
PGR	<0.001	0.417631	0.354843	0.491528

(*Continued*)

Table S1: *Continued*

Gene	*P* value	HR	lower 95% CI	upper 95% CI
PGRMC1	<0.001	0.447416	0.388955	0.514662
PGRMC2	<0.001	0.42454	0.372662	0.483641
PGS1	<0.001	0.268243	0.213497	0.337027
PHEX	<0.001	0.477318	0.4166	0.546886
PHF8	<0.001	0.29446	0.222502	0.389689
PHGDH	0.006198	0.831812	0.729051	0.949056
PHKA1	<0.001	0.455052	0.397935	0.520366
PHKA2	<0.001	0.284824	0.216076	0.375444
PHKG1	0.002412	1.282423	1.09206	1.505968
PHKG2	0.086913	1.23228	0.970189	1.565172
PHLPP1	0.001989	0.70503	0.564917	0.879895
PHLPP2	<0.001	0.327838	0.271555	0.395787
PHOX2B	0.06401	1.118668	0.993491	1.259615
PHYH	<0.001	0.572998	0.520693	0.630557
PI4KA	<0.001	0.377409	0.321682	0.44279
PICK1	<0.001	0.338598	0.26591	0.431156
PIGA	<0.001	0.587358	0.541192	0.637461
PIGB	<0.001	0.502928	0.450244	0.561776
PIGC	<0.001	0.319411	0.268237	0.380348
PIGF	<0.001	0.399798	0.347974	0.45934
PIGG	<0.001	0.183619	0.137737	0.244786
PIGH	<0.001	0.33053	0.271173	0.40288
PIGK	<0.001	0.406192	0.35304	0.467347
PIGL	<0.001	0.297669	0.245246	0.361297
PIGN	<0.001	0.419914	0.36548	0.482455
PIGO	0.94579	0.990601	0.754526	1.300538
PIGP	<0.001	0.42812	0.371771	0.493009
PIGQ	<0.001	0.276453	0.21421	0.35678
PIGT	<0.001	0.293455	0.234919	0.366578
PIGV	<0.001	0.408536	0.350356	0.476378
PIGZ	2.22E-16	0.474768	0.397251	0.567411
PIK3C2A	<0.001	0.403696	0.349966	0.465675
PIK3C2B	<0.001	0.466979	0.410273	0.531524
PIK3C2G	<0.001	0.562283	0.49267	0.641733
PIK3C3	<0.001	0.350868	0.297184	0.414251
PIK3CA	<0.001	0.522948	0.472378	0.578932
PIK3CB	<0.001	0.573409	0.512429	0.641645
PIK3CD	<0.001	0.33579	0.275139	0.409812
PIK3CG	<0.001	0.477618	0.417453	0.546455
PIK3R1	<0.001	0.531464	0.478386	0.590431

(*Continued*)

Table S1: *Continued*

Gene	*P* value	HR	lower 95% CI	upper 95% CI
PIK3R2	0.132895	0.80416	0.605185	1.068555
PIK3R3	<0.001	0.452425	0.394094	0.519389
PIK3R4	<0.001	0.288615	0.230096	0.362017
PIKFYVE	<0.001	0.260403	0.210668	0.321879
PIM1	<0.001	0.477773	0.411297	0.554992
PIM2	0.001024	1.24646	1.092881	1.42162
PINK1	<0.001	0.357835	0.301846	0.424209
PIP4K2A	<0.001	0.453745	0.397825	0.517524
PIP4K2B	<0.001	0.088337	0.058593	0.133181
PIP4K2C	<0.001	0.383645	0.318407	0.462249
PIP5K1C	<0.001	0.351626	0.288887	0.427991
PIPOX	<0.001	0.587808	0.523456	0.660071
PIR	6.94E-09	0.753493	0.684678	0.829224
PITPNA	<0.001	0.255334	0.204688	0.318513
PITPNB	<0.001	0.452182	0.394178	0.518721
PITPNC1	<0.001	0.333811	0.268389	0.415179
PITPNM1	4.38E-11	0.396314	0.300956	0.521886
PITPNM3	1.68E-05	0.587001	0.460556	0.748161
PJA1	<0.001	0.388636	0.333149	0.453364
PKD2	<0.001	0.519737	0.470707	0.573874
PKD2L1	0.223701	0.902654	0.765373	1.064557
PKIA	<0.001	0.579081	0.525056	0.638666
PKIG	<0.001	0.501191	0.434125	0.578616
PKLR	4.26E-07	1.549876	1.307798	1.836762
PKM	0.160967	0.885544	0.747139	1.049587
PKMYT1	0.000553	1.484922	1.186478	1.858434
PKN1	0.001094	0.683287	0.543656	0.858781
PKN2	<0.001	0.481083	0.428257	0.540426
PLA1A	1.53E-08	0.712942	0.634091	0.8016
PLA2G12A	<0.001	0.417382	0.365455	0.476687
PLA2G15	1.11E-16	0.416694	0.33896	0.512254
PLA2G16	<0.001	0.539708	0.48264	0.603524
PLA2G1B	<0.001	0.706007	0.65415	0.761974
PLA2G2A	0.022857	1.113277	1.015004	1.221065
PLA2G2D	2.43E-11	1.30958	1.20991	1.41746
PLA2G2E	0.001469	1.236721	1.084962	1.409707
PLA2G2F	<0.001	0.431843	0.365096	0.510793
PLA2G3	0.436298	0.959822	0.865669	1.064216
PLA2G4A	<0.001	0.737896	0.693739	0.784863
PLA2G4C	<0.001	0.454397	0.387586	0.532724

(*Continued*)

Table S1: *Continued*

Gene	*P* value	HR	lower 95% CI	upper 95% CI
PLA2G5	6.44E-15	0.38179	0.299697	0.486371
PLA2G6	2.22E-15	0.350946	0.27093	0.454594
PLA2G7	2.44E-15	0.658296	0.593578	0.73007
PLAG1	<0.001	0.591184	0.542167	0.644631
PLAT	3.43E-10	0.760151	0.697776	0.828101
PLAU	0.097959	0.915143	0.823904	1.016485
PLCB1	<0.001	0.454789	0.391243	0.528657
PLCB2	0.00047	1.446028	1.176009	1.778047
PLCB3	<0.001	0.234436	0.170722	0.321927
PLCB4	<0.001	0.654039	0.60419	0.708002
PLCD1	<0.001	0.335105	0.27736	0.404871
PLCE1	<0.001	0.323972	0.269848	0.388951
PLCG1	<0.001	0.16916	0.126152	0.22683
PLCG2	<0.001	0.248637	0.197043	0.313741
PLCH1	<0.001	0.440679	0.379061	0.512313
PLCH2	1.33E-10	1.469599	1.306721	1.652779
PLCXD1	0.878489	0.985681	0.819286	1.18587
PLD1	1.11E-16	0.544925	0.471741	0.629463
PLD2	<0.001	0.220955	0.165441	0.295097
PLD3	2.00E-14	0.569629	0.493151	0.657968
PLEKHG6	1.66E-11	1.736641	1.478887	2.039317
PLEKHM1	<0.001	0.316984	0.256659	0.391488
PLG	0.124188	1.181895	0.955109	1.462531
PLIN1	8.91E-07	1.293291	1.167211	1.43299
PLIN3	0.007334	1.400538	1.094855	1.791568
PLK1	0.24404	0.927383	0.816911	1.052795
PLK2	<0.001	0.640019	0.585912	0.699123
PLK3	<0.001	0.174274	0.125208	0.242567
PLK4	6.39E-13	0.635527	0.561673	0.719093
PLN	<0.001	0.659315	0.612826	0.709332
PLOD1	9.72E-08	0.579515	0.474207	0.708209
PLOD2	0.002301	0.856834	0.775805	0.946326
PLOD3	5.20E-10	0.569417	0.476734	0.680119
PLS1	<0.001	0.669058	0.628365	0.712386
PLSCR1	<0.001	0.477663	0.417738	0.546185
PLSCR2	<0.001	0.451017	0.378477	0.537461
PLSCR4	<0.001	0.642559	0.596988	0.691608
PLTP	0.129766	1.11443	0.968682	1.282108
PLXNB1	1.58E-14	1.470003	1.332368	1.621856
PMF1	<0.001	0.445231	0.384647	0.515356

(*Continued*)

Table S1: *Continued*

Gene	*P* value	HR	lower 95% CI	upper 95% CI
PMM1	<0.001	0.40333	0.345718	0.470543
PMM2	<0.001	0.406272	0.338704	0.487319
PMPCA	<0.001	0.310186	0.244129	0.394118
PMVK	<0.001	0.327243	0.257456	0.415947
PNKP	9.29E-12	0.498583	0.408164	0.609032
PNLIP	3.29E-07	1.441897	1.252929	1.659366
PNLIPRP1	8.53E-07	1.507082	1.28001	1.774437
PNLIPRP2	0.245667	0.928398	0.818958	1.052464
PNMT	3.84E-06	1.332339	1.179642	1.504801
PNP	<0.001	0.526076	0.457423	0.605035
PNPLA2	0.001271	1.27703	1.100544	1.481818
PNPLA3	2.15E-10	0.616441	0.530936	0.715717
PNPLA4	<0.001	0.455709	0.396795	0.523369
PNPLA6	1.19E-13	0.482726	0.398225	0.585157
PNPO	<0.001	0.35499	0.292825	0.430352
POFUT1	<0.001	0.27533	0.218973	0.346191
POFUT2	2.87E-07	1.80592	1.440998	2.263256
POLA1	<0.001	0.50529	0.449251	0.568319
POLA2	<0.001	0.318883	0.24487	0.415267
POLB	<0.001	0.37858	0.320809	0.446755
POLD1	<0.001	0.371899	0.302525	0.457181
POLD2	1.04E-09	0.535469	0.438139	0.65442
POLD3	<0.001	0.273669	0.220375	0.339853
POLE	1.34E-07	0.486652	0.372354	0.636036
POLE2	2.58E-13	0.665457	0.596659	0.742187
POLE3	<0.001	0.400323	0.339895	0.471493
POLG	0.000169	0.570236	0.425517	0.764173
POLG2	<0.001	0.403694	0.35028	0.465252
POLH	<0.001	0.214906	0.163751	0.282041
POLI	<0.001	0.55245	0.504629	0.604804
POLL	7.09E-10	1.662901	1.414616	1.954765
POLM	0.03348	0.807124	0.662459	0.983379
POLQ	3.72E-05	0.760389	0.667581	0.866099
POLR1B	<0.001	0.32288	0.268588	0.388148
POLR1C	<0.001	0.363572	0.301836	0.437937
POLR1D	<0.001	0.280457	0.222006	0.354298
POLR1E	<0.001	0.321569	0.261824	0.394946
POLR2A	0.957424	0.992766	0.760476	1.296009
POLR2B	<0.001	0.432856	0.379951	0.493128
POLR2C	<0.001	0.395956	0.331558	0.472862

(*Continued*)

Table S1: *Continued*

Gene	*P* value	HR	lower 95% CI	upper 95% CI
POLR2D	<0.001	0.372069	0.306529	0.451622
POLR2E	0.000225	0.591725	0.447778	0.781947
POLR2F	0.381398	0.908032	0.731618	1.126985
POLR2G	<0.001	0.345474	0.269989	0.442064
POLR2H	<0.001	0.471221	0.405939	0.547001
POLR2I	0.391279	1.11758	0.86677	1.440964
POLR2J	<0.001	1.965568	1.697486	2.275987
POLR2K	<0.001	0.49619	0.435752	0.565009
POLR2L	0.014917	0.746506	0.589944	0.944617
POLR3B	<0.001	0.342188	0.2866	0.408559
POLR3C	<0.001	0.364055	0.305786	0.433428
POLR3D	1.11E-16	0.337297	0.260973	0.435943
POLR3F	<0.001	0.39842	0.34451	0.460767
POLR3G	<0.001	0.331601	0.273499	0.402047
POLR3K	<0.001	0.326011	0.260829	0.407482
POLRMT	0.136397	1.192373	0.94592	1.50304
POMGNT1	<0.001	0.235454	0.183218	0.302584
POMT1	<0.001	0.407334	0.348218	0.476486
POMT2	<0.001	0.233548	0.177061	0.308057
PON1	<0.001	0.448666	0.387626	0.519316
PON2	<0.001	0.550089	0.494468	0.611966
PON3	<0.001	0.754372	0.71184	0.799445
POR	<0.001	0.496509	0.4281	0.575849
POSTN	<0.001	0.637855	0.577951	0.703968
PPA1	<0.001	0.480522	0.428164	0.539283
PPA2	<0.001	0.304567	0.251461	0.368889
PPAP2A	<0.001	0.456776	0.399241	0.522602
PPAP2B	<0.001	0.379427	0.325485	0.442309
PPAP2C	<0.001	0.59332	0.528289	0.666357
PPARA	<0.001	0.239553	0.189945	0.302116
PPARD	<0.001	0.304439	0.237504	0.390238
PPARG	<0.001	0.641685	0.580908	0.70882
PPAT	<0.001	0.544889	0.486948	0.609724
PPBP	2.70E-05	0.848545	0.7859	0.916183
PPCDC	<0.001	0.412887	0.349343	0.48799
PPCS	<0.001	0.429777	0.375068	0.492466
PPEF1	1.37E-14	0.659928	0.593673	0.733577
PPEF2	0.454864	0.94674	0.820148	1.092871
PPFIBP1	<0.001	0.520562	0.459622	0.589581
PPIA	0.000569	1.398633	1.155658	1.692694

(*Continued*)

Table S1: *Continued*

Gene	*P* value	HR	lower 95% CI	upper 95% CI
PPIB	0.003556	1.524062	1.148049	2.023227
PPIF	0.000148	1.31954	1.143441	1.52276
PPIG	<0.001	0.513183	0.46142	0.570753
PPIH	<0.001	0.435402	0.371503	0.510292
PPIP5K1	<0.001	0.309472	0.25004	0.383031
PPIP5K2	<0.001	0.570715	0.524404	0.621116
PPM1A	<0.001	0.481585	0.43037	0.538894
PPM1B	<0.001	0.472086	0.42173	0.528455
PPM1D	<0.001	0.416403	0.362642	0.478134
PPM1E	1.78E-15	0.569067	0.495273	0.653857
PPM1F	<0.001	0.264296	0.208943	0.334313
PPM1G	1.18E-05	0.536776	0.406348	0.709068
PPM1H	<0.001	0.477117	0.410158	0.555008
PPME1	<0.001	0.358612	0.290643	0.442477
PPOX	<0.001	0.212166	0.164947	0.272901
PPP1CA	0.454094	0.898881	0.679967	1.188274
PPP1CB	<0.001	0.519451	0.470865	0.57305
PPP1CC	<0.001	0.439945	0.386227	0.501134
PPP1R1A	0.678426	1.031441	0.891025	1.193985
PPP1R3C	<0.001	0.573436	0.513693	0.640128
PPP1R8	<0.001	0.341655	0.29081	0.401389
PPP2CA	<0.001	0.38596	0.332978	0.447373
PPP2CB	<0.001	0.321422	0.258654	0.399421
PPP3CA	<0.001	0.497773	0.44537	0.556341
PPP3CB	<0.001	0.442239	0.3844	0.50878
PPP3CC	<0.001	0.24189	0.190249	0.307549
PPP3R1	0.175263	1.12783	0.947768	1.342102
PPP4C	<0.001	0.42611	0.353934	0.513006
PPP5C	2.53E-05	0.600967	0.474173	0.761666
PPP6C	<0.001	0.374577	0.321774	0.436046
PPT1	<0.001	0.375877	0.319643	0.442004
PRDM2	<0.001	0.280047	0.226975	0.345527
PRDM9	1.85E-07	1.435026	1.252818	1.643734
PRDX1	<0.001	0.447278	0.382585	0.522911
PRDX2	<0.001	0.244539	0.182191	0.328223
PRDX3	<0.001	0.362831	0.309892	0.424813
PRDX4	<0.001	0.46381	0.388897	0.553154
PRDX6	<0.001	0.322758	0.260953	0.3992
PREB	<0.001	0.270448	0.214646	0.340757
PREP	<0.001	0.424774	0.366755	0.491971

(*Continued*)

Table S1: *Continued*

Gene	*P* value	HR	lower 95% CI	upper 95% CI
PREX2	<0.001	0.383413	0.323589	0.454297
PRF1	1.19E-08	0.61371	0.518872	0.725882
PRG2	1.21E-13	0.619833	0.546229	0.703356
PRIM1	<0.001	0.558888	0.499197	0.625717
PRIM2	1.71E-14	0.554468	0.47691	0.64464
PRKAA1	<0.001	0.463314	0.407916	0.526234
PRKAA2	<0.001	0.548564	0.489411	0.614866
PRKACA	0.778396	1.035488	0.812199	1.320164
PRKACB	<0.001	0.544182	0.494144	0.599286
PRKACG	1.70E-05	1.34998	1.177437	1.547809
PRKAR1A	<0.001	0.364862	0.314232	0.42365
PRKAR1B	0.81303	1.02378	0.842609	1.243906
PRKAR2A	<0.001	0.210139	0.161142	0.274033
PRKAR2B	<0.001	0.596079	0.549975	0.646048
PRKCA	0.000459	0.644634	0.504248	0.824104
PRKCB	<0.001	0.294491	0.236635	0.366493
PRKCD	4.44E-08	0.562472	0.457718	0.6912
PRKCDBP	7.14E-14	0.617388	0.544149	0.700486
PRKCE	<0.001	0.274405	0.217797	0.345725
PRKCG	3.50E-08	1.668001	1.390671	2.000637
PRKCH	<0.001	0.293954	0.226421	0.381628
PRKCI	<0.001	0.501419	0.431434	0.582757
PRKCQ	<0.001	0.436955	0.374653	0.509618
PRKCSH	2.80E-07	0.59734	0.49072	0.727127
PRKCZ	<0.001	0.501051	0.443781	0.565713
PRKD1	<0.001	0.439246	0.375398	0.513953
PRKD2	<0.001	0.214663	0.168321	0.273763
PRKD3	<0.001	0.438359	0.38391	0.50053
PRKDC	7.44E-05	0.629398	0.500549	0.791414
PRKG1	<0.001	0.412237	0.35465	0.479175
PRKG2	<0.001	0.458798	0.399957	0.526294
PRKX	<0.001	0.572615	0.513112	0.639019
PRKY	7.77E-16	0.575293	0.50297	0.658015
PRMT1	2.22E-16	1.578471	1.415901	1.759708
PRMT2	<0.001	0.34513	0.290322	0.410286
PRMT3	<0.001	0.560189	0.512528	0.612283
PRMT5	<0.001	0.438559	0.369292	0.520819
PRMT7	<0.001	0.307522	0.243657	0.388128
PRNP	<0.001	0.53404	0.473231	0.602664
PROC	0.162862	1.088191	0.966393	1.225339

(*Continued*)

Table S1: *Continued*

Gene	*P* value	HR	lower 95% CI	upper 95% CI
PRODH2	0.381503	0.902219	0.716518	1.136048
PROS1	<0.001	0.650738	0.607414	0.697152
PROSC	1.78E-15	0.360364	0.280272	0.463343
PROZ	0.020224	1.204496	1.029446	1.409312
PRPF19	<0.001	0.402147	0.328721	0.491975
PRPF4	0.000313	0.635257	0.496354	0.81303
PRPF4B	<0.001	0.547597	0.498144	0.60196
PRPS1	<0.001	0.364377	0.303956	0.436808
PRPS1L1	4.41E-07	0.754265	0.676075	0.841498
PRPS2	<0.001	0.580957	0.529724	0.637144
PRRX1	<0.001	0.588886	0.531809	0.652089
PRSS2	0.027623	1.091865	1.009729	1.180684
PRSS3	0.779271	0.983537	0.875737	1.104606
PRUNE	6.19E-11	0.431601	0.335511	0.555212
PSAP	<0.001	0.339577	0.269991	0.427099
PSAT1	0.724246	1.019487	0.915835	1.13487
PSD	0.006408	1.237418	1.061701	1.442218
PSD3	<0.001	0.554054	0.50189	0.61164
PSD4	<0.001	0.324948	0.264382	0.399388
PSEN1	<0.001	0.203568	0.157223	0.263575
PSKH1	4.88E-13	0.380902	0.293194	0.494848
PSPH	<0.001	0.631242	0.57151	0.697218
PTAFR	9.44E-09	0.546657	0.444797	0.671843
PTDSS1	1.11E-16	0.504499	0.429752	0.592246
PTDSS2	0.005634	0.673958	0.509692	0.891165
PTEN	<0.001	0.509049	0.455927	0.56836
PTGDR	<0.001	0.388733	0.313845	0.481491
PTGDS	2.93E-06	0.783511	0.707341	0.867883
PTGER1	1.06E-05	1.420439	1.215025	1.660581
PTGER2	<0.001	0.547166	0.488778	0.61253
PTGER3	<0.001	0.229662	0.174	0.303131
PTGER4	<0.001	0.473633	0.401626	0.558551
PTGES	0.092459	1.14074	0.978525	1.329846
PTGES2	2.05E-05	0.573658	0.444207	0.740833
PTGES3	<0.001	0.394004	0.339384	0.457414
PTGFR	<0.001	0.509571	0.443562	0.585403
PTGIR	0.028153	1.26814	1.025777	1.567766
PTGIS	0.001875	0.724428	0.591214	0.887659
PTGS1	<0.001	0.501657	0.435594	0.577739
PTGS2	5.62E-14	0.805235	0.761012	0.852027

(*Continued*)

Table S1: *Continued*

Gene	*P* value	HR	lower 95% CI	upper 95% CI
PTH	0.016254	0.870327	0.777121	0.974711
PTH1R	<0.001	0.276731	0.227499	0.336617
PTHLH	0.5708	1.019282	0.95415	1.088859
PTK2	<0.001	0.508822	0.452771	0.571811
PTK2B	0.063872	1.229008	0.988175	1.528535
PTK6	0.000561	0.789295	0.690019	0.902855
PTK7	8.72E-06	1.668799	1.331606	2.091377
PTN	1.33E-14	0.616972	0.54563	0.697643
PTP4A1	<0.001	0.494683	0.426005	0.574433
PTP4A2	<0.001	0.405904	0.350738	0.469748
PTP4A3	1.84E-08	0.571804	0.470634	0.694721
PTPLA	<0.001	0.48729	0.428291	0.554416
PTPLAD1	<0.001	0.426827	0.368554	0.494313
PTPLB	<0.001	0.560648	0.502519	0.625501
PTPN1	0.499915	1.10436	0.82768	1.473529
PTPN11	<0.001	0.391013	0.329004	0.464709
PTPN12	<0.001	0.449246	0.380947	0.52979
PTPN13	<0.001	0.570973	0.522864	0.623508
PTPN14	<0.001	0.493667	0.431113	0.565298
PTPN18	<0.001	0.30992	0.243613	0.394275
PTPN2	<0.001	0.268676	0.213999	0.337322
PTPN21	<0.001	0.266015	0.209723	0.337417
PTPN22	<0.001	0.495671	0.438298	0.560555
PTPN3	<0.001	0.441301	0.385509	0.505167
PTPN4	<0.001	0.435499	0.382601	0.495709
PTPN6	<0.001	0.345715	0.279289	0.427941
PTPN7	<0.001	0.285878	0.231309	0.35332
PTPN9	1.97E-11	0.350807	0.258316	0.476415
PTPRA	<0.001	0.236671	0.177545	0.315488
PTPRB	<0.001	0.440621	0.381776	0.508536
PTPRC	<0.001	0.536941	0.485057	0.594375
PTPRD	<0.001	0.465797	0.406833	0.533308
PTPRE	<0.001	0.322231	0.268217	0.387123
PTPRF	7.15E-05	0.65576	0.532481	0.807579
PTPRG	<0.001	0.434742	0.381782	0.49505
PTPRH	0.436522	1.036095	0.947564	1.132896
PTPRJ	<0.001	0.262124	0.203915	0.33695
PTPRK	<0.001	0.556828	0.505554	0.613302
PTPRM	<0.001	0.540087	0.489518	0.59588
PTPRN	9.62E-08	0.707322	0.622813	0.803297

(*Continued*)

Table S1: *Continued*

Gene	*P* value	HR	lower 95% CI	upper 95% CI
PTPRN2	1.11E-16	0.515797	0.441307	0.60286
PTPRO	<0.001	0.405507	0.335599	0.489977
PTPRR	7.35E-13	0.646312	0.573647	0.728181
PTPRS	0.774881	1.034077	0.821903	1.301022
PTPRT	0.003938	1.144562	1.04418	1.254594
PTPRU	0.506382	0.92977	0.750059	1.152539
PTPRZ1	3.43E-09	0.857537	0.814919	0.902382
PTRH2	<0.001	0.434156	0.376482	0.500666
PTS	<0.001	0.551207	0.482198	0.630092
PUF60	1.54E-11	0.466371	0.373647	0.582105
PUS1	<0.001	0.440706	0.382809	0.507358
PUS3	<0.001	0.370136	0.315754	0.433884
PUS7	<0.001	0.56076	0.502719	0.625501
PUS7L	<0.001	0.432938	0.376016	0.498477
PVALB	0.85694	0.989522	0.882444	1.109592
PXDN	5.88E-15	0.677756	0.614692	0.747289
PYCR1	8.72E-08	0.595051	0.492023	0.719654
PYCRL	0.009268	1.273899	1.061554	1.528721
PYGB	0.044868	0.803553	0.648939	0.995005
PYGL	2.04E-09	0.724645	0.65222	0.805112
PYGM	2.76E-08	0.709334	0.6284	0.800692
PYROXD1	<0.001	0.526712	0.475957	0.582879
QDPR	<0.001	0.308545	0.253193	0.375998
QPCT	5.46E-06	0.817986	0.750117	0.891996
QPCTL	<0.001	0.370683	0.304022	0.451961
QPRT	2.93E-06	0.801269	0.730206	0.879249
QRSL1	<0.001	0.358406	0.306252	0.419441
QSOX1	<0.001	0.473149	0.415375	0.538958
QTRT1	1.20E-07	0.543024	0.433145	0.680778
QTRTD1	<0.001	0.371374	0.30903	0.446295
RAB11FIP2	<0.001	0.528652	0.479104	0.583323
RAB11FIP5	<0.001	0.271535	0.220219	0.334809
RAB15	3.04E-12	0.601277	0.521197	0.69366
RAB1A	<0.001	0.333261	0.280837	0.39547
RAB1B	<0.001	0.38696	0.323226	0.463261
RAB3GAP1	<0.001	0.293776	0.242479	0.355926
RAB3GAP2	<0.001	0.466056	0.405938	0.535076
RAB3IL1	<0.001	0.394151	0.331599	0.468504
RAB5A	<0.001	0.377787	0.321089	0.444496
RAB5B	<0.001	0.277024	0.215277	0.356482

(*Continued*)

Table S1: *Continued*

Gene	*P* value	HR	lower 95% CI	upper 95% CI
RABEP1	<0.001	0.448856	0.397022	0.507456
RABEP2	3.33E-16	0.360079	0.281591	0.460445
RABEPK	0.001056	0.739637	0.617497	0.885935
RABGGTA	<0.001	0.341077	0.27432	0.42408
RABGGTB	<0.001	0.55764	0.507696	0.612497
RAC1	<0.001	0.301808	0.244396	0.372706
RAC2	7.00E-08	0.647334	0.552669	0.758215
RAC3	<0.001	1.382394	1.28632	1.485644
RACGAP1	4.65E-06	0.742423	0.653576	0.843348
RAD54L2	<0.001	0.226201	0.168276	0.304065
RAF1	<0.001	0.389147	0.330918	0.457622
RAG1	<0.001	0.342297	0.281777	0.415816
RALBP1	<0.001	0.374041	0.312813	0.447254
RALGDS	4.66E-07	1.748336	1.406913	2.172615
RALGPS1	<0.001	0.183311	0.141089	0.23817
RALGPS2	<0.001	0.511833	0.458014	0.571976
RAN	<0.001	0.423799	0.368449	0.487464
RANBP1	0.004233	1.319913	1.091295	1.596424
RANBP17	<0.001	0.437101	0.380252	0.502451
RANBP2	<0.001	0.549021	0.500832	0.601847
RANBP3	0.355318	1.136538	0.866407	1.49089
RANBP9	<0.001	0.387968	0.335857	0.448165
RANGRF	5.82E-12	0.360771	0.269879	0.482275
RAP1A	<0.001	0.25479	0.190933	0.340003
RAP1B	<0.001	0.437802	0.384468	0.498535
RAP1GDS1	<0.001	0.465687	0.412147	0.526183
RAP2A	<0.001	0.347586	0.293721	0.41133
RAP2B	<0.001	0.469505	0.39925	0.552123
RAPGEF2	<0.001	0.488046	0.433347	0.54965
RAPGEF3	5.75E-05	0.731264	0.627839	0.851727
RAPGEF4	<0.001	0.516804	0.463858	0.575793
RAPGEF5	<0.001	0.330035	0.276785	0.393528
RAPGEF6	<0.001	0.480842	0.428241	0.539904
RAPGEFL1	2.30E-10	1.297652	1.197221	1.406508
RARA	0.001014	1.533183	1.188272	1.978208
RARB	<0.001	0.328988	0.258209	0.419168
RARG	0.001151	1.362166	1.13057	1.641205
RARRES1	0.004945	0.867683	0.785918	0.957955
RARS	<0.001	0.472503	0.418108	0.533975
RARS2	<0.001	0.317963	0.248232	0.407282

(*Continued*)

Table S1: *Continued*

Gene	*P* value	HR	lower 95% CI	upper 95% CI
RASGRF1	<0.001	0.467402	0.399112	0.547377
RASGRP1	<0.001	0.594571	0.547027	0.646248
RASGRP2	0.284781	0.870326	0.674773	1.122551
RASGRP3	<0.001	0.33995	0.277925	0.415817
RASIP1	5.10E-05	1.570502	1.26237	1.953847
RASSF1	<0.001	0.477027	0.414357	0.549176
RASSF2	<0.001	0.490898	0.434277	0.554901
RASSF4	<0.001	0.406887	0.338106	0.48966
RBKS	<0.001	0.467903	0.401101	0.545831
RBP1	2.23E-12	1.501239	1.34024	1.681579
RCAN1	<0.001	0.466018	0.389863	0.557048
RCC1	1.13E-10	0.455303	0.35846	0.578309
RCN1	<0.001	0.476418	0.411294	0.551853
RCN2	<0.001	0.509669	0.458359	0.566724
RCVRN	1.96E-11	1.699499	1.455581	1.984291
RDH11	<0.001	0.311441	0.254485	0.381145
RDH16	0.233157	0.899441	0.755629	1.070624
RDH8	0.015829	1.188032	1.032867	1.366507
RECQL	<0.001	0.575003	0.523975	0.631
RECQL4	2.56E-06	1.402208	1.21796	1.614327
RECQL5	2.36E-08	0.4125	0.302291	0.562889
REG1A	3.39E-06	1.227149	1.125616	1.337841
REG1B	0.036254	0.864989	0.755187	0.990755
REM1	<0.001	0.276789	0.219618	0.348843
REN	0.51494	0.960737	0.851619	1.083836
RENBP	3.33E-16	1.46642	1.337862	1.607331
RET	2.23E-12	0.519238	0.432404	0.623509
RETSAT	<0.001	0.505855	0.442232	0.57863
REV3L	<0.001	0.540635	0.489761	0.596793
REXO2	<0.001	0.355445	0.301076	0.419634
RFK	<0.001	0.537958	0.481895	0.600544
RFWD3	2.86E-09	0.565259	0.468266	0.682342
RGL1	<0.001	0.575884	0.521712	0.635681
RGL2	<0.001	0.404844	0.349311	0.469206
RGN	<0.001	0.604903	0.554965	0.659334
RGR	0.001238	1.33394	1.119968	1.588792
RHAG	0.171151	0.862604	0.698041	1.065961
RHBG	2.49E-10	1.386215	1.252853	1.533772
RHCG	3.18E-08	1.193018	1.120693	1.270011
RHEB	<0.001	0.263117	0.206504	0.33525

(*Continued*)

Table S1: *Continued*

Gene	*P* value	HR	lower 95% CI	upper 95% CI
RHO	5.63E-13	0.525078	0.440719	0.625584
RHOA	0.003959	0.661948	0.499973	0.876398
RHOD	2.66E-06	0.606847	0.492648	0.747518
RHOF	4.61E-06	0.596411	0.478113	0.74398
RHOQ	<0.001	0.364509	0.308442	0.430768
RHOT1	<0.001	0.453134	0.400947	0.512113
RIC8B	<0.001	0.265637	0.216157	0.326444
RIMS1	0.084476	0.876893	0.755324	1.01803
RIMS2	3.33E-15	0.495044	0.415598	0.589676
RIN1	1.69E-06	0.505122	0.381927	0.668055
RIN2	<0.001	0.415436	0.357518	0.482738
RIN3	1.11E-16	0.352929	0.275791	0.451643
RIOK2	<0.001	0.495797	0.445417	0.551875
RIPK1	<0.001	0.416997	0.362221	0.480056
RIPK2	<0.001	0.501355	0.43199	0.581858
RIT1	<0.001	0.57587	0.528066	0.628001
RIT2	0.6077	0.968515	0.857159	1.094338
RNASE1	8.86E-06	0.717643	0.619938	0.830747
RNASE3	0.077311	1.115362	0.988114	1.258997
RNASEH1	<0.001	0.351247	0.29407	0.419542
RNASEH2A	2.75E-05	1.336541	1.167067	1.530625
RNASEL	<0.001	0.466813	0.411715	0.529284
RND1	9.54E-14	0.682342	0.617041	0.754552
RND3	<0.001	0.656203	0.606159	0.710378
RNF128	4.44E-16	0.811354	0.771507	0.853259
RNF138	<0.001	0.542395	0.493506	0.596128
RNF2	<0.001	0.321418	0.268136	0.385288
RNF5	0.611636	0.952823	0.790669	1.148231
RNF8	<0.001	0.380098	0.30714	0.470386
RNGTT	<0.001	0.409668	0.352479	0.476136
RNLS	<0.001	0.382273	0.324651	0.450123
RNMT	<0.001	0.490069	0.435333	0.551687
RNPEP	<0.001	0.423348	0.359299	0.498815
ROCK1	<0.001	0.37341	0.301558	0.462381
ROCK2	<0.001	0.469008	0.41584	0.528975
ROR1	<0.001	0.508708	0.454185	0.569776
ROR2	0.000941	0.808681	0.713063	0.91712
RORA	<0.001	0.423156	0.367235	0.487593
RORB	<0.001	0.405972	0.338406	0.487028
ROS1	<0.001	0.579966	0.526101	0.639346

(*Continued*)

Table S1: *Continued*

Gene	*P* value	HR	lower 95% CI	upper 95% CI
RP2	<0.001	0.365147	0.307393	0.433753
RPAP2	<0.001	0.362317	0.306608	0.428149
RPE	<0.001	0.496386	0.44386	0.555129
RPE65	2.87E-08	0.725633	0.647923	0.812664
RPH3AL	2.57E-10	0.507076	0.410821	0.625884
RPIA	<0.001	0.402925	0.348364	0.46603
RPL22	<0.001	0.316592	0.26445	0.379014
RPL29	0.534563	1.113973	0.7924	1.566048
RPN1	0.379828	0.873098	0.644955	1.181943
RPN2	<0.001	0.358705	0.294959	0.436227
RPP14	<0.001	0.259589	0.20971	0.321332
RPS27	7.89E-14	0.392713	0.307327	0.501823
RPS6KA1	<0.001	0.455935	0.386656	0.537626
RPS6KA2	<0.001	0.489519	0.42992	0.55738
RPS6KA3	<0.001	0.479302	0.428669	0.535915
RPS6KA4	0.000131	1.543338	1.235647	1.927647
RPS6KA5	<0.001	0.409782	0.351934	0.477139
RPS6KA6	<0.001	0.540275	0.469265	0.622032
RPS6KB1	<0.001	0.493778	0.441357	0.552425
RPS6KB2	<0.001	0.337161	0.273034	0.416349
RPUSD2	<0.001	0.294589	0.23483	0.369556
RRAGA	<0.001	0.321802	0.268437	0.385776
RRAGB	<0.001	0.419217	0.362039	0.485425
RRAGC	<0.001	0.414932	0.356091	0.483496
RRAGD	<0.001	0.560872	0.505498	0.622312
RRAS2	<0.001	0.468972	0.412231	0.533522
RREB1	2.48E-07	0.34298	0.228412	0.515015
RRM1	<0.001	0.506239	0.435245	0.588814
RRM2	0.005641	0.875934	0.79751	0.96207
RSAD1	<0.001	0.246296	0.198132	0.306169
RSAD2	<0.001	0.634093	0.581463	0.691486
RTCA	<0.001	0.462931	0.406784	0.526827
RTEL1	3.36E-08	0.439725	0.328493	0.588623
RUFY1	<0.001	0.219262	0.167998	0.286168
RUNDC3A	0.885962	0.987772	0.834902	1.168633
RUVBL1	0.003589	1.29605	1.088492	1.543188
RUVBL2	1.32E-10	0.454206	0.357023	0.577844
RXFP3	1.11E-16	1.72934	1.519229	1.96851
RXRA	<0.001	0.369937	0.297597	0.45986
RXRB	4.49E-07	0.540669	0.425809	0.686511

(*Continued*)

Table S1: *Continued*

Gene	*P* value	HR	lower 95% CI	upper 95% CI
RXRG	8.52E-11	0.626303	0.543782	0.721347
RYK	<0.001	0.439423	0.380565	0.507384
RYR1	0.017693	0.896442	0.819014	0.981189
RYR2	<0.001	0.420272	0.360383	0.490114
RYR3	<0.001	0.450481	0.383852	0.528676
S100A1	0.000571	1.213039	1.086814	1.353924
S100A10	<0.001	0.6014	0.537915	0.672377
S100A11	<0.001	1.918189	1.654624	2.223737
S100A2	0.000928	1.095084	1.037768	1.155566
S100A3	0.294287	1.064119	0.947437	1.195171
S100A4	0.059132	1.148353	0.994682	1.325766
S100A8	0.000466	1.138309	1.058648	1.223963
S100A9	3.85E-09	1.214925	1.138719	1.296231
S100B	0.000254	0.798568	0.707895	0.900854
S100P	0.001724	1.079164	1.028953	1.131826
S1PR1	<0.001	0.551874	0.493953	0.616586
S1PR2	0.028287	0.703612	0.513933	0.963295
S1PR4	0.000526	0.676943	0.542955	0.843996
S1PR5	<0.001	1.506561	1.37226	1.654006
SAE1	1.63E-05	1.548071	1.269111	1.888347
SAG	5.47E-09	0.63601	0.546281	0.740477
SAR1A	<0.001	0.367775	0.305248	0.443111
SAR1B	<0.001	0.43198	0.378566	0.492932
SARDH	6.07E-10	1.639243	1.401729	1.917001
SARS	<0.001	0.368277	0.307783	0.440661
SARS2	<0.001	0.392652	0.323283	0.476907
SAT1	<0.001	0.358206	0.300839	0.426513
SCAP	<0.001	0.225753	0.175083	0.291086
SCARB1	2.06E-09	0.468131	0.365223	0.600035
SCCPDH	<0.001	0.456457	0.398631	0.522672
SCD	0.000113	1.259178	1.120118	1.415504
SCD5	<0.001	0.307534	0.25128	0.37638
SCG2	1.67E-15	0.681579	0.620213	0.749017
SCGB1A1	0.000169	0.933057	0.899967	0.967364
SCIN	1.09E-11	0.750345	0.690678	0.815166
SCLY	<0.001	0.25512	0.193245	0.336806
SCN10A	<0.001	0.389787	0.33081	0.459279
SCN11A	<0.001	0.285397	0.225798	0.360726
SCN1A	<0.001	0.622684	0.5575	0.69549
SCN1B	0.910857	0.987971	0.799345	1.221108

(*Continued*)

Table S1: *Continued*

Gene	*P* value	HR	lower 95% CI	upper 95% CI
SCN2A	0.003934	0.825707	0.72492	0.940507
SCN2B	6.00E-15	0.460651	0.379157	0.559661
SCN3A	9.60E-12	0.77998	0.726164	0.837783
SCN3B	3.80E-05	0.771898	0.682435	0.87309
SCN4A	0.15028	0.902746	0.785281	1.037782
SCN5A	<0.001	0.353512	0.287518	0.434653
SCN7A	<0.001	0.621659	0.574017	0.673254
SCN8A	0.150386	0.870775	0.721122	1.051485
SCN9A	<0.001	0.547509	0.492584	0.608559
SCNN1A	0.532203	1.059524	0.883731	1.270287
SCNN1B	6.72E-09	0.748958	0.679231	0.825843
SCNN1D	7.85E-07	1.480662	1.267107	1.73021
SCNN1G	<0.001	0.497942	0.434173	0.571076
SCO2	<0.001	0.511045	0.439636	0.594053
SCP2	<0.001	0.520659	0.473259	0.572806
SCRN1	<0.001	0.535093	0.481817	0.594261
SDC1	6.99E-15	0.600739	0.528409	0.682971
SDC2	<0.001	0.525901	0.471563	0.5865
SDC4	<0.001	0.631754	0.567501	0.703283
SDF4	<0.001	0.300549	0.240222	0.376024
SDHA	<0.001	0.317377	0.254032	0.396517
SDHB	<0.001	0.266735	0.214902	0.331069
SDHC	<0.001	0.265076	0.208198	0.337492
SDHD	<0.001	0.324936	0.270472	0.390368
SDPR	<0.001	0.596373	0.548128	0.648864
SDS	0.155178	0.874287	0.72645	1.052211
SEC14L3	0.627992	1.028724	0.917384	1.153576
SEC14L4	4.33E-15	0.506377	0.4272	0.600229
SEC16A	<0.001	0.396352	0.338019	0.46475
SEC31A	<0.001	0.377232	0.317844	0.447715
SELENBP1	<0.001	0.707836	0.66472	0.753748
SELL	<0.001	0.620335	0.565802	0.680123
SELP	<0.001	0.487555	0.429933	0.5529
SELPLG	0.000351	0.695478	0.569901	0.848726
SELT	<0.001	0.449209	0.390934	0.51617
SEPHS1	<0.001	0.377112	0.322301	0.441246
SEPHS2	<0.001	0.377953	0.321656	0.444102
SEPP1	<0.001	0.547757	0.498522	0.601855
SEPW1	<0.001	0.416494	0.361181	0.480278
SERGEF	<0.001	0.329655	0.254595	0.426844

(*Continued*)

Table S1: *Continued*

Gene	*P* value	HR	lower 95% CI	upper 95% CI
SERINC1	<0.001	0.517188	0.470249	0.568813
SERINC5	<0.001	0.521178	0.467759	0.580698
SERPINA10	0.000543	1.213548	1.087491	1.354218
SERPINA7	3.17E-05	0.749696	0.654564	0.858654
SERPINC1	0.00204	1.278186	1.093602	1.493924
SERPIND1	1.49E-13	0.797979	0.751605	0.847213
SERPINE1	0.787323	0.982459	0.863934	1.117244
SET	6.66E-16	2.043008	1.717562	2.430119
SETD1A	0.035088	1.213864	1.013646	1.453628
SETD1B	<0.001	0.220097	0.169149	0.286392
SETD2	<0.001	0.494078	0.439627	0.555273
SETD3	<0.001	0.318374	0.264346	0.383445
SETD8	7.77E-16	0.412155	0.332154	0.511426
SETDB1	<0.001	0.265554	0.212436	0.331953
SETMAR	<0.001	0.295641	0.243924	0.358323
SFPQ	<0.001	0.360639	0.308561	0.421506
SFRP4	<0.001	0.69897	0.652012	0.749309
SFXN1	<0.001	0.472875	0.411254	0.543728
SFXN3	5.66E-15	0.506099	0.426611	0.600396
SGK1	<0.001	0.631045	0.575101	0.692432
SGK2	1.93E-12	0.566972	0.484112	0.664013
SGMS1	<0.001	0.607003	0.562339	0.655215
SGPL1	<0.001	0.458572	0.390552	0.538439
SGPP1	<0.001	0.56166	0.510144	0.618378
SGSH	<0.001	0.421056	0.361862	0.489932
SHBG	<0.001	1.566425	1.412819	1.736732
SHMT1	6.66E-15	0.452418	0.370587	0.552317
SHMT2	0.037861	0.806327	0.658056	0.988005
SHPK	<0.001	0.343414	0.274704	0.429308
SI	0.768509	0.985283	0.892651	1.087527
SIAH1	<0.001	0.380524	0.325339	0.445071
SIAH2	0.022071	0.804469	0.667739	0.969197
SIGLEC7	8.74E-12	2.056522	1.671942	2.529564
SIGMAR1	0.022489	0.725115	0.55019	0.955654
SIK1	<0.001	0.412997	0.337627	0.505191
SIK2	<0.001	0.210227	0.163434	0.270418
SIK3	<0.001	0.289152	0.233312	0.358357
SIRT1	<0.001	0.516628	0.466684	0.571916
SIRT2	<0.001	0.338481	0.274119	0.417955
SIRT3	<0.001	0.139818	0.10395	0.188063

(*Continued*)

Table S1: *Continued*

Gene	*P* value	HR	lower 95% CI	upper 95% CI
SIRT4	<0.001	0.3671	0.310707	0.433728
SIRT5	<0.001	0.241637	0.191781	0.304453
SIRT6	5.56E-08	0.450646	0.33802	0.600797
SIRT7	<0.001	0.28258	0.223788	0.356816
SKIV2L2	<0.001	0.401163	0.348081	0.46234
SLC10A1	1.80E-08	0.667959	0.580416	0.768705
SLC10A2	<0.001	0.450159	0.381546	0.531111
SLC10A3	<0.001	0.397027	0.334197	0.47167
SLC11A1	0.000416	1.374264	1.151876	1.639589
SLC11A2	<0.001	0.416825	0.357381	0.486156
SLC12A1	8.89E-11	0.576622	0.488226	0.681022
SLC12A2	<0.001	0.578351	0.530253	0.63081
SLC12A3	6.26E-08	1.663478	1.383451	2.000185
SLC12A4	0.00166	1.47569	1.157931	1.880648
SLC12A5	<0.001	0.375389	0.315553	0.446571
SLC12A6	<0.001	0.443362	0.387943	0.506699
SLC12A7	<0.001	0.438524	0.377495	0.50942
SLC12A8	0.679476	0.97434	0.861304	1.10221
SLC13A1	0.881066	0.987233	0.834293	1.168209
SLC13A2	0.000114	0.787239	0.697147	0.888973
SLC13A3	0.019579	0.799208	0.66211	0.964694
SLC13A4	0.401821	0.936584	0.803578	1.091605
SLC14A1	<0.001	0.502654	0.436291	0.579111
SLC15A1	6.66E-16	0.481753	0.403519	0.575156
SLC15A2	<0.001	0.571441	0.519386	0.628713
SLC16A1	0.019788	0.866078	0.767429	0.977407
SLC16A10	<0.001	0.554673	0.494769	0.621831
SLC16A2	1.11E-16	0.520033	0.44622	0.606056
SLC16A4	<0.001	0.703593	0.655273	0.755476
SLC16A5	<0.001	0.491128	0.431501	0.558994
SLC16A6	<0.001	0.506044	0.443867	0.576931
SLC16A7	<0.001	0.642489	0.594545	0.694298
SLC16A8	7.15E-06	1.406178	1.21173	1.631829
SLC17A1	0.075771	1.148248	0.985764	1.337514
SLC17A2	0.001562	0.799389	0.695828	0.918363
SLC17A3	4.17E-13	0.608988	0.532574	0.696365
SLC17A4	0.788476	0.976497	0.820757	1.16179
SLC17A5	<0.001	0.409425	0.353916	0.473641
SLC17A6	5.43E-12	0.647183	0.571875	0.732409
SLC17A7	0.828611	1.019482	0.856076	1.214079

(*Continued*)

Table S1: *Continued*

Gene	*P* value	HR	lower 95% CI	upper 95% CI
SLC18A1	0.240819	0.927717	0.818404	1.051631
SLC18A2	<0.001	0.44705	0.391123	0.510974
SLC18A3	2.17E-08	1.414063	1.252521	1.59644
SLC19A1	1.93E-07	1.444857	1.257926	1.659567
SLC19A2	<0.001	0.545376	0.498143	0.597089
SLC19A3	<0.001	0.456772	0.390119	0.534813
SLC1A1	<0.001	0.56981	0.511524	0.634737
SLC1A2	<0.001	0.419622	0.342959	0.513422
SLC1A3	2.20E-14	0.58177	0.506276	0.66852
SLC1A4	<0.001	0.464008	0.388887	0.553639
SLC1A5	9.04E-10	0.541171	0.444642	0.658656
SLC1A6	1.25E-09	1.292577	1.189846	1.404179
SLC1A7	1.78E-13	0.590656	0.513427	0.679501
SLC20A1	4.75E-14	0.614621	0.54156	0.697539
SLC20A2	1.44E-15	0.485938	0.406998	0.58019
SLC22A1	0.243955	0.921236	0.802485	1.057558
SLC22A11	2.03E-07	1.30868	1.182416	1.448426
SLC22A2	1.46E-08	1.436389	1.26728	1.628065
SLC22A3	<0.001	0.649015	0.598573	0.703709
SLC22A4	<0.001	0.5602	0.493461	0.635965
SLC22A5	<0.001	0.289222	0.230778	0.362467
SLC22A6	2.12E-14	1.549333	1.384794	1.733422
SLC22A7	0.006183	1.352798	1.089652	1.679492
SLC23A2	<0.001	0.337922	0.280267	0.407438
SLC24A1	<0.001	0.239469	0.187862	0.305252
SLC24A2	3.15E-08	0.626571	0.530942	0.739425
SLC24A3	<0.001	0.623064	0.567008	0.684663
SLC25A1	1.14E-05	1.589694	1.292484	1.955247
SLC25A10	0.008618	0.75254	0.608703	0.930366
SLC25A11	<0.001	0.280633	0.221145	0.356123
SLC25A12	<0.001	0.418542	0.365261	0.479596
SLC25A13	3.70E-14	0.480832	0.397805	0.581189
SLC25A15	1.19E-05	0.673269	0.56401	0.803693
SLC25A20	<0.001	0.352963	0.293421	0.424587
SLC25A22	<0.001	0.242611	0.190318	0.309272
SLC25A23	<0.001	0.347256	0.28677	0.4205
SLC25A24	<0.001	0.386981	0.329979	0.453828
SLC25A28	<0.001	0.279968	0.224443	0.34923
SLC25A3	<0.001	0.359828	0.301745	0.429092
SLC25A32	<0.001	0.44202	0.386922	0.504964

(*Continued*)

Table S1: *Continued*

Gene	*P* value	HR	lower 95% CI	upper 95% CI
SLC25A37	7.50E-12	0.547604	0.460906	0.650611
SLC25A4	<0.001	0.317406	0.265973	0.378786
SLC25A42	6.99E-15	0.364664	0.282878	0.470096
SLC25A5	<0.001	0.353036	0.29563	0.421589
SLC25A6	<0.001	0.36549	0.307336	0.434649
SLC26A1	8.86E-09	1.690394	1.41349	2.021544
SLC26A10	0.202281	0.910948	0.789277	1.051374
SLC26A2	<0.001	0.514098	0.457426	0.577791
SLC26A3	0.000172	0.711093	0.595226	0.849513
SLC26A4	7.33E-15	0.678271	0.615069	0.747968
SLC27A2	7.11E-15	0.705107	0.645707	0.769971
SLC27A3	<0.001	0.486208	0.418012	0.565531
SLC27A5	8.88E-16	1.794431	1.555997	2.069402
SLC27A6	0.652107	0.977508	0.885461	1.079123
SLC28A1	1.38E-06	1.478496	1.261487	1.732837
SLC28A2	0.511412	0.952773	0.824664	1.100784
SLC28A3	3.33E-16	0.645205	0.580834	0.71671
SLC29A1	<0.001	0.384616	0.318033	0.465138
SLC29A2	0.449583	0.923061	0.750071	1.135949
SLC29A3	<0.001	0.438552	0.36297	0.529871
SLC2A1	0.31547	1.05527	0.950048	1.172145
SLC2A10	<0.001	0.685037	0.635417	0.738532
SLC2A11	<0.001	0.253125	0.199454	0.321238
SLC2A2	1.37E-12	1.509676	1.347137	1.691826
SLC2A3	4.08E-06	0.733416	0.642788	0.836822
SLC2A4	0.405103	1.058854	0.92548	1.211448
SLC2A5	5.08E-08	0.550582	0.44421	0.682426
SLC2A6	0.005047	1.258867	1.071754	1.478648
SLC2A8	<0.001	0.229198	0.176023	0.298438
SLC2A9	0.394987	0.906537	0.723093	1.136521
SLC30A1	2.52E-13	0.416889	0.329799	0.526978
SLC30A10	0.00993	1.148995	1.033867	1.276942
SLC30A3	<0.001	1.444502	1.33145	1.567152
SLC30A4	1.97E-11	0.500936	0.409323	0.613054
SLC30A5	<0.001	0.420383	0.367823	0.480453
SLC30A9	<0.001	0.544997	0.495997	0.598838
SLC31A1	<0.001	0.457462	0.397003	0.527128
SLC31A2	<0.001	0.530902	0.464345	0.607
SLC33A1	<0.001	0.327926	0.271328	0.396331
SLC34A1	0.001182	0.711686	0.579456	0.87409

(*Continued*)

Table S1: *Continued*

Gene	*P* value	HR	lower 95% CI	upper 95% CI
SLC34A2	<0.001	0.793817	0.754662	0.835003
SLC35A1	<0.001	0.556995	0.509946	0.608385
SLC35A3	<0.001	0.554528	0.504121	0.609974
SLC35D1	<0.001	0.399216	0.342348	0.46553
SLC35D2	<0.001	0.294635	0.237666	0.36526
SLC36A1	<0.001	0.347031	0.279272	0.43123
SLC37A1	<0.001	0.343151	0.271144	0.43428
SLC37A4	5.91E-09	0.481726	0.376676	0.616074
SLC38A1	<0.001	0.476547	0.418315	0.542886
SLC38A10	0.100553	1.248133	0.958015	1.626109
SLC38A2	<0.001	0.547955	0.499241	0.601422
SLC38A3	7.09E-07	1.302281	1.17319	1.445577
SLC38A4	0.016573	0.838497	0.725982	0.96845
SLC38A6	<0.001	0.572477	0.519916	0.630352
SLC39A1	2.22E-16	0.433246	0.354937	0.528831
SLC39A14	<0.001	0.383136	0.312837	0.469233
SLC39A2	0.017122	0.840481	0.728588	0.969559
SLC39A4	0.663961	0.969622	0.843648	1.114407
SLC39A6	<0.001	0.517523	0.458308	0.584388
SLC39A7	3.33E-16	0.495857	0.41891	0.586938
SLC39A8	<0.001	0.391182	0.328267	0.466154
SLC39A9	1.33E-14	0.392993	0.309868	0.498418
SLC3A1	<0.001	0.362414	0.289921	0.453033
SLC3A2	1.59E-07	0.568488	0.460267	0.702155
SLC43A1	4.09E-14	0.465724	0.382005	0.567791
SLC44A1	<0.001	0.405906	0.351769	0.468375
SLC44A4	1.42E-05	0.78885	0.708733	0.878023
SLC48A1	<0.001	0.324181	0.250651	0.419281
SLC4A10	2.08E-06	0.741078	0.654805	0.838718
SLC4A4	<0.001	0.52804	0.47168	0.591134
SLC4A5	<0.001	0.247851	0.199022	0.308661
SLC4A7	<0.001	0.414617	0.355225	0.483939
SLC4A8	<0.001	0.414188	0.353745	0.48496
SLC50A1	2.33E-15	1.603673	1.426778	1.802501
SLC52A1	1.22E-11	0.714049	0.647786	0.787091
SLC52A2	3.49E-09	0.558294	0.460116	0.67742
SLC5A1	0.001241	0.836379	0.750425	0.932178
SLC5A12	0.000582	0.757489	0.646618	0.88737
SLC5A2	1.97E-08	0.629219	0.535259	0.739674
SLC5A3	<0.001	0.494009	0.435456	0.560435

(*Continued*)

Table S1: *Continued*

Gene	*P* value	HR	lower 95% CI	upper 95% CI
SLC5A4	3.48E-05	0.755517	0.661608	0.862756
SLC5A5	7.64E-05	1.391705	1.181463	1.63936
SLC5A6	1.50E-05	0.577413	0.450279	0.740442
SLC5A7	0.420281	0.951042	0.841752	1.074523
SLC6A1	<0.001	0.535631	0.476172	0.602515
SLC6A11	9.99E-16	0.357734	0.278376	0.459715
SLC6A12	<0.001	0.503707	0.434724	0.583636
SLC6A13	<0.001	0.338783	0.280013	0.409887
SLC6A14	1.11E-16	0.824996	0.788361	0.863334
SLC6A15	0.917988	1.00705	0.880993	1.151145
SLC6A16	<0.001	0.476408	0.413021	0.549522
SLC6A2	5.04E-08	1.996011	1.556735	2.559242
SLC6A20	7.77E-16	0.630275	0.563405	0.70508
SLC6A3	0.025976	0.882773	0.791009	0.985181
SLC6A4	1.81E-09	0.703427	0.627232	0.788878
SLC6A5	0.019582	1.281813	1.040614	1.578919
SLC6A6	0.324863	0.885164	0.694319	1.128466
SLC6A7	<0.001	1.40348	1.297743	1.517834
SLC6A8	4.79E-08	1.254646	1.156506	1.361113
SLC6A9	0.099908	0.882592	0.760583	1.024173
SLC7A1	0.029453	1.321306	1.028209	1.697951
SLC7A10	5.37E-07	1.369044	1.21081	1.547956
SLC7A11	0.000109	0.830526	0.755956	0.912453
SLC7A2	5.55E-16	0.512843	0.436223	0.602919
SLC7A4	3.18E-06	1.416113	1.223286	1.639335
SLC7A5	0.381204	1.057683	0.932916	1.199136
SLC7A6	<0.001	0.424427	0.367822	0.489743
SLC7A7	2.22E-16	0.607424	0.539154	0.684339
SLC7A8	9.41E-14	0.492853	0.409133	0.593704
SLC7A9	<0.001	0.40215	0.340856	0.474467
SLC8A1	<0.001	0.277108	0.212868	0.360735
SLC8A2	1.17E-12	1.421636	1.290218	1.566441
SLC9A1	8.19E-14	0.385004	0.299682	0.494619
SLC9A2	0.00069	0.84551	0.76741	0.931559
SLC9A3	1.50E-13	1.708822	1.482383	1.969849
SLC9A3R1	3.45E-13	0.610465	0.534464	0.697272
SLC9A3R2	2.23E-08	1.53476	1.320826	1.783345
SLC9A5	<0.001	0.307819	0.244874	0.386946
SLC9A6	<0.001	0.447791	0.396475	0.50575
SLC9A8	<0.001	0.33736	0.276657	0.411382

(*Continued*)

Table S1: *Continued*

Gene	*P* value	HR	lower 95% CI	upper 95% CI
SLCO1A2	<0.001	0.403702	0.329502	0.494612
SLCO1B1	8.74E-11	0.647441	0.567753	0.738313
SLCO1B3	0.446918	0.976791	0.919433	1.037727
SLCO1C1	<0.001	0.310806	0.250593	0.385487
SLCO2A1	<0.001	0.6559	0.594979	0.72306
SLCO2B1	6.16E-11	0.588779	0.502352	0.690076
SLCO3A1	<0.001	0.518994	0.455251	0.591661
SLCO4A1	0.02027	1.178281	1.025867	1.353338
SLCO4C1	<0.001	0.51478	0.461897	0.573718
SLK	<0.001	0.57552	0.528708	0.626477
SLN	2.72E-07	0.765322	0.691141	0.847466
SMG6	<0.001	0.31216	0.248674	0.391854
SMOX	0.023697	1.25841	1.031167	1.535731
SMPD1	1.36E-05	0.55832	0.429364	0.726005
SMPD2	<0.001	0.381479	0.30709	0.473889
SMPD3	7.11E-12	0.506627	0.417117	0.615345
SMS	<0.001	0.548075	0.487407	0.616294
SMURF1	0.09036	0.839841	0.686226	1.027844
SMURF2	<0.001	0.506821	0.457181	0.561852
SMYD2	2.50E-10	0.420203	0.321233	0.549665
SMYD3	<0.001	0.420779	0.357019	0.495926
SNAP25	<0.001	0.486736	0.414661	0.571339
SNCA	<0.001	0.402565	0.333254	0.486291
SNCB	4.39E-06	1.351617	1.188508	1.53711
SNCG	0.716765	1.030414	0.876426	1.211457
SNRK	<0.001	0.337846	0.281661	0.405239
SNTA1	3.74E-08	0.461828	0.350733	0.608113
SNTB1	<0.001	0.501941	0.440648	0.571759
SNTB2	<0.001	0.160399	0.117225	0.219473
SNTG1	0.000743	0.682506	0.54666	0.852111
SOAT1	<0.001	0.478158	0.425263	0.537633
SOAT2	0.553267	1.044206	0.905068	1.204733
SOD1	<0.001	0.389921	0.332438	0.457343
SOD3	1.45E-07	0.600195	0.496209	0.725973
SORBS2	<0.001	0.470802	0.411096	0.539178
SORBS3	0.000115	0.545163	0.400548	0.741989
SORD	2.14E-09	0.604821	0.513018	0.713052
SORT1	<0.001	0.475892	0.416981	0.543126
SOS1	<0.001	0.359408	0.305563	0.42274
SOS2	<0.001	0.265739	0.21385	0.330217

(*Continued*)

Table S1: *Continued*

Gene	*P* value	HR	lower 95% CI	upper 95% CI
SP1	1.11E-09	0.402123	0.299978	0.539049
SPAG1	<0.001	0.583577	0.53218	0.639937
SPAM1	<0.001	0.395126	0.320612	0.486959
SPARC	<0.001	0.523492	0.465008	0.589331
SPAST	<0.001	0.535304	0.48451	0.591423
SPCS1	<0.001	0.371717	0.314548	0.439277
SPHK1	0.026333	0.847132	0.731776	0.980674
SPHK2	<0.001	0.295704	0.237173	0.368679
SPOCK2	<0.001	0.363458	0.292213	0.452075
SPR	2.00E-08	0.473511	0.364698	0.614789
SPRY4	<0.001	0.514911	0.455708	0.581805
SPTAN1	<0.001	0.330572	0.265105	0.412205
SPTBN1	<0.001	0.197248	0.150686	0.258197
SPTLC1	<0.001	0.377467	0.31486	0.452524
SPTLC2	<0.001	0.31549	0.260268	0.382428
SPTLC3	<0.001	0.56812	0.513809	0.628172
SQLE	<0.001	0.550529	0.485869	0.623794
SQRDL	<0.001	0.567897	0.499976	0.645046
SRC	7.96E-09	1.989216	1.574754	2.51276
SRD5A1	1.11E-16	0.615243	0.548671	0.689891
SRD5A2	<0.001	0.480978	0.415123	0.55728
SRD5A3	<0.001	0.586592	0.524908	0.655524
SRI	<0.001	0.497648	0.445275	0.556181
SRM	0.094974	1.225273	0.965301	1.55526
SRP54	<0.001	0.515182	0.463211	0.572985
SRPK1	<0.001	0.422465	0.361363	0.493899
SRPK2	<0.001	0.387925	0.331906	0.453398
SRPK3	0.803069	1.017907	0.885371	1.170284
SRPR	<0.001	0.541017	0.483318	0.605605
SRR	<0.001	0.396353	0.341462	0.460068
SRY	8.29E-09	1.444071	1.274408	1.636323
SSH1	<0.001	0.378847	0.312947	0.458626
SSH3	2.23E-11	0.38546	0.291528	0.509656
SSR1	<0.001	0.403866	0.349492	0.466701
SSR2	<0.001	0.263578	0.206647	0.336194
SSR3	<0.001	0.39152	0.331001	0.463104
SSR4	3.78E-10	0.499778	0.402262	0.620933
SST	0.212465	0.952076	0.88134	1.028489
SSTR2	2.09E-12	0.467054	0.377715	0.577525
ST3GAL1	<0.001	0.535657	0.462937	0.6198

(*Continued*)

Table S1: *Continued*

Gene	*P* value	HR	lower 95% CI	upper 95% CI
ST3GAL2	6.81E-08	0.408448	0.295051	0.565428
ST3GAL4	0.42068	0.922511	0.758076	1.122614
ST3GAL5	<0.001	0.655852	0.608812	0.706527
ST3GAL6	<0.001	0.41944	0.363961	0.483377
ST6GAL1	<0.001	0.408911	0.336889	0.496331
ST6GALNAC2	1.90E-06	0.770779	0.692479	0.857934
ST6GALNAC4	0.014161	0.72831	0.565345	0.938252
ST8SIA1	<0.001	0.530274	0.472868	0.594649
STAB1	<0.001	0.281417	0.216026	0.366601
STAB2	0.019398	1.198967	1.029761	1.395975
STAC	<0.001	0.4831	0.422047	0.552986
STAMBP	<0.001	0.369976	0.316319	0.432735
STATH	0.000193	0.796568	0.706803	0.897733
STAU2	<0.001	0.430098	0.376341	0.491535
STC2	0.003373	0.804781	0.696009	0.930552
STEAP1	9.26E-11	0.765383	0.705908	0.829868
STEAP3	1.11E-15	0.568063	0.49472	0.652278
STEAP4	<0.001	0.713315	0.670747	0.758585
STIM1	<0.001	0.289587	0.229946	0.364696
STK10	<0.001	0.43282	0.364944	0.513319
STK11	0.254283	1.172808	0.891679	1.542572
STK16	0.004038	1.369938	1.105388	1.697801
STK17A	<0.001	0.521056	0.469065	0.578811
STK17B	<0.001	0.44581	0.390979	0.508332
STK19	<0.001	0.211127	0.163592	0.272474
STK24	4.44E-16	0.243982	0.17361	0.342878
STK25	<0.001	0.292393	0.238288	0.358783
STK3	<0.001	0.432804	0.375853	0.498384
STK32B	3.07E-11	0.744961	0.682983	0.812564
STK38	<0.001	0.342413	0.278055	0.421667
STK38L	<0.001	0.553976	0.504171	0.6087
STK39	<0.001	0.572939	0.523805	0.626682
STK4	<0.001	0.477856	0.416461	0.548301
STRN3	<0.001	0.511921	0.459667	0.570115
STRN4	0.001351	0.686643	0.545613	0.864126
STS	<0.001	0.512783	0.449095	0.585503
STT3A	0.172198	0.868921	0.710192	1.063128
STUB1	<0.001	0.269273	0.209436	0.346207
STXBP1	<0.001	0.652082	0.602663	0.705553
STYK1	<0.001	0.638238	0.582637	0.699146

(*Continued*)

Table S1: *Continued*

Gene	*P* value	HR	lower 95% CI	upper 95% CI
SUCLA2	<0.001	0.568336	0.521759	0.61907
SUCLG1	<0.001	0.33402	0.278656	0.400383
SUCLG2	<0.001	0.499212	0.44773	0.556614
SULF1	9.68E-10	0.751093	0.68525	0.823262
SULT1A1	<0.001	0.334093	0.273243	0.408493
SULT1A2	<0.001	0.449911	0.375627	0.538887
SULT1E1	3.89E-15	0.578715	0.504928	0.663285
SULT2A1	0.058101	1.165563	0.994756	1.3657
SULT2B1	1.17E-06	0.700999	0.607449	0.808956
SUOX	<0.001	0.395399	0.339592	0.460376
SUPV3L1	<0.001	0.343853	0.288462	0.40988
SUSD5	<0.001	0.63994	0.582314	0.703269
SUV39H1	2.56E-13	0.491742	0.406586	0.594733
SUV39H2	<0.001	0.471982	0.412077	0.540596
SUV420H1	<0.001	0.459281	0.408847	0.515936
SV2A	0.039984	1.127048	1.005486	1.263307
SV2B	1.46E-10	0.533466	0.440212	0.646474
SV2C	<0.001	0.370262	0.303801	0.451262
SWAP70	<0.001	0.448658	0.397903	0.505887
SYK	<0.001	0.446853	0.385853	0.517497
SYN3	0.703046	0.979592	0.881062	1.089142
SYNJ1	<0.001	0.353012	0.298749	0.41713
SYNJ2	0.011737	0.675206	0.497479	0.916426
SYP	3.15E-10	1.356232	1.233409	1.491286
SYT1	0.000928	0.853676	0.77737	0.937473
SYT11	<0.001	0.389398	0.325846	0.465345
SYT13	3.02E-09	0.689112	0.60932	0.779354
SYT2	0.913348	1.007317	0.883367	1.148658
SYT5	0.265386	1.092202	0.935185	1.275583
SYTL2	<0.001	0.568552	0.515428	0.62715
TACR1	0.998762	0.999832	0.808291	1.236762
TACR2	0.000104	1.282084	1.130863	1.453527
TACR3	0.743165	1.019518	0.908197	1.144483
TAF1	<0.001	0.233938	0.185045	0.295748
TAF4	6.60E-10	0.472202	0.372136	0.599175
TAGLN	0.074461	0.844796	0.701889	1.016799
TALDO1	<0.001	0.43959	0.371889	0.519616
TAOK1	<0.001	0.323712	0.269364	0.389026
TAOK2	0.815865	1.037113	0.763173	1.409384
TAOK3	<0.001	0.416857	0.363702	0.477782

(*Continued*)

Table S1: *Continued*

Gene	*P* value	HR	lower 95% CI	upper 95% CI
TAP1	1.31E-12	0.627863	0.552097	0.714026
TAP2	4.11E-15	0.439366	0.357821	0.539494
TARBP1	<0.001	0.429696	0.37609	0.490943
TARS	<0.001	0.427043	0.364416	0.500433
TAS2R10	<0.001	0.480337	0.424978	0.542906
TAS2R16	0.004476	0.843601	0.750253	0.948564
TAS2R3	1.49E-12	1.465081	1.318004	1.628571
TAS2R4	6.37E-08	0.691236	0.60465	0.79022
TAS2R7	0.020512	1.176501	1.025354	1.349928
TAS2R9	<0.001	0.547732	0.481654	0.622875
TAT	1.04E-06	0.586088	0.472993	0.726226
TBCD	8.74E-11	0.372487	0.276398	0.501981
TBXA2R	0.003641	1.514871	1.144963	2.004287
TBXAS1	<0.001	0.430455	0.357151	0.518803
TCHH	<0.001	0.567423	0.503515	0.639443
TCIRG1	<0.001	0.541104	0.47421	0.617433
TCN1	0.421984	0.981802	0.938761	1.026816
TCN2	<0.001	0.4648	0.392742	0.550077
TDG	<0.001	0.570698	0.520241	0.62605
TDO2	6.97E-12	0.652333	0.577362	0.737039
TEC	3.91E-10	0.678055	0.600362	0.765801
TECR	<0.001	0.273875	0.219028	0.342456
TENC1	<0.001	0.392251	0.330589	0.465415
TERT	0.007596	0.771226	0.637309	0.933283
TESC	0.035994	1.147587	1.009036	1.305163
TESK1	<0.001	0.300574	0.237835	0.379863
TESK2	<0.001	0.332283	0.272967	0.404489
TET3	2.55E-15	0.349076	0.268965	0.453049
TF	0.810217	0.984953	0.870313	1.114693
TFB1M	<0.001	0.263456	0.209079	0.331973
TFB2M	1.12E-05	1.46625	1.236057	1.739312
TFF1	0.007207	0.912611	0.853726	0.975558
TFR2	<0.001	0.28957	0.233455	0.359174
TFRC	1.36E-13	0.545624	0.464743	0.640582
TGDS	<0.001	0.408852	0.356869	0.468408
TGFBI	1.41E-09	0.697864	0.621148	0.784056
TGFBR1	<0.001	0.497849	0.439308	0.56419
TGFBR2	<0.001	0.451049	0.382555	0.531807
TGM1	9.98E-10	1.32674	1.211704	1.452697
TGM2	4.57E-06	0.610143	0.493946	0.753673

(*Continued*)

Table S1: *Continued*

Gene	*P* value	HR	lower 95% CI	upper 95% CI
TGM3	0.035951	0.861845	0.750054	0.990299
TGM4	0.021963	0.75722	0.596892	0.960611
TGM5	0.656227	1.033168	0.894901	1.192798
TGS1	2.60E-14	0.467721	0.384656	0.568725
TH	9.11E-05	1.257425	1.121125	1.410297
THG1L	<0.001	0.453117	0.3936	0.521633
THNSL1	<0.001	0.500765	0.448173	0.559527
THNSL2	1.11E-16	0.609211	0.541535	0.685344
THRA	<0.001	0.283941	0.214677	0.375552
THRB	<0.001	0.517281	0.463312	0.577537
THTPA	<0.001	0.384555	0.318529	0.464267
TIAM1	<0.001	0.482225	0.416538	0.55827
TIAM2	<0.001	0.330008	0.27525	0.39566
TIE1	1.23E-10	0.513066	0.418701	0.628698
TIPARP	<0.001	0.602491	0.54958	0.660497
TK1	0.000151	1.303469	1.136474	1.495004
TK2	<0.001	0.326168	0.265334	0.400948
TKT	0.326752	0.91057	0.755089	1.098067
TKTL1	0.805311	1.014482	0.904878	1.137362
TLK1	<0.001	0.279235	0.225397	0.345932
TLK2	<0.001	0.245712	0.194399	0.31057
TLR4	<0.001	0.493934	0.436301	0.55918
TM7SF2	1.77E-09	0.64372	0.557687	0.743024
TMEM132A	2.92E-08	1.738838	1.430073	2.114267
TMEM38B	<0.001	0.522726	0.465282	0.587263
TMLHE	<0.001	0.438051	0.372201	0.515551
TMPRSS6	8.36E-08	1.62041	1.358192	1.933254
TNF	<0.001	0.523481	0.451594	0.606812
TNFAIP6	3.58E-10	0.744014	0.678339	0.816047
TNFRSF11B	<0.001	0.617957	0.557471	0.685006
TNFSF11	0.036602	0.869997	0.76349	0.991362
TNIK	<0.001	0.51	0.45289	0.574311
TNK1	2.18E-09	0.473981	0.371154	0.605297
TNK2	3.95E-06	1.629041	1.324041	2.0043
TNKS	<0.001	0.230746	0.178721	0.297914
TNKS2	<0.001	0.360429	0.304179	0.427082
TNNC1	0.196672	0.95833	0.898367	1.022296
TNNC2	0.409939	0.961178	0.874784	1.056104
TNNI1	4.00E-13	1.377068	1.263047	1.501382
TNNI2	5.97E-11	1.300511	1.202093	1.406986

(*Continued*)

Table S1: *Continued*

Gene	*P* value	HR	lower 95% CI	upper 95% CI
TNNI3	1.10E-06	0.752857	0.67162	0.843921
TNNT1	3.95E-09	1.190142	1.123121	1.261161
TNNT2	0.024935	1.151345	1.017909	1.302273
TNNT3	0.025194	1.252009	1.028352	1.52431
TNPO1	<0.001	0.401626	0.347492	0.464193
TNPO2	0.460494	1.105184	0.847398	1.44139
TNR	6.64E-07	0.566824	0.453156	0.709003
TOP2A	2.73E-05	0.821389	0.74924	0.900486
TOP2B	<0.001	0.5294	0.48148	0.582089
TOPORS	<0.001	0.48543	0.432832	0.54442
TP53I3	<0.001	0.537457	0.467093	0.618419
TPCN1	<0.001	0.223809	0.167373	0.299274
TPH1	4.51E-08	0.579037	0.476072	0.704271
TPI1	0.409971	1.115671	0.859922	1.447483
TPK1	<0.001	0.557964	0.507708	0.613194
TPM1	<0.001	0.376945	0.318582	0.446001
TPM2	9.46E-06	1.425454	1.218499	1.66756
TPM3	<0.001	0.325574	0.267954	0.395585
TPM4	<0.001	0.488303	0.428665	0.556237
TPMT	<0.001	0.261691	0.206495	0.331641
TPO	2.66E-11	1.442674	1.29526	1.606866
TPPP	<0.001	0.292498	0.240135	0.356278
TPST1	<0.001	0.475047	0.415613	0.54298
TPST2	<0.001	0.468696	0.398023	0.551917
TPTE	0.119987	0.923209	0.834754	1.021037
TRDMT1	<0.001	0.342005	0.287207	0.407258
TREH	8.55E-15	1.669828	1.466979	1.900728
TRHR	1.11E-16	1.563366	1.407076	1.737015
TRIM25	<0.001	0.256524	0.204528	0.32174
TRIM32	<0.001	0.425826	0.366717	0.494463
TRIM33	<0.001	0.464051	0.410677	0.524362
TRIM5	<0.001	0.392139	0.32739	0.469694
TRIO	1.92E-08	0.433213	0.323582	0.579988
TRIP10	2.12E-12	0.5215	0.434891	0.625356
TRIP12	<0.001	0.44396	0.391226	0.503803
TRIT1	<0.001	0.453003	0.397781	0.51589
TRMT1	2.02E-11	0.412121	0.318039	0.534035
TRMT11	<0.001	0.575014	0.527655	0.626623
TRMT12	<0.001	0.3922	0.330601	0.465277
TRMT13	<0.001	0.582399	0.533843	0.635372

(*Continued*)

Table S1: *Continued*

Gene	*P* value	HR	lower 95% CI	upper 95% CI
TRMT2B	<0.001	0.355759	0.295955	0.427646
TRMT44	<0.001	0.141514	0.101916	0.196496
TRMT5	<0.001	0.39124	0.331875	0.461224
TRMT61A	0.000246	0.540764	0.389301	0.751155
TRMT61B	<0.001	0.479403	0.427535	0.537564
TRMU	<0.001	0.286192	0.231103	0.354412
TRPA1	0.748404	1.020831	0.899994	1.157892
TRPC1	<0.001	0.504733	0.451019	0.564845
TRPC3	<0.001	0.295108	0.239143	0.364171
TRPC4	0.000173	0.700245	0.581396	0.84339
TRPC5	1.70E-07	0.733903	0.653546	0.824141
TRPC6	<0.001	0.546208	0.493805	0.604172
TRPC7	3.89E-13	1.66031	1.447904	1.903875
TRPM1	0.010372	0.740386	0.588353	0.931705
TRPM2	5.53E-09	0.534547	0.433056	0.659825
TRPM3	0.000571	0.609432	0.45979	0.807775
TRPM4	1.34E-07	0.647705	0.551146	0.761182
TRPM6	0.091362	0.831655	0.671432	1.030111
TRPM8	1.91E-05	0.749423	0.656584	0.855389
TRPS1	<0.001	0.508603	0.449933	0.574923
TRPV2	<0.001	0.379372	0.306063	0.470241
TRPV4	4.63E-05	1.353574	1.17009	1.565829
TRPV5	<0.001	0.394138	0.320315	0.484975
TRPV6	2.03E-05	1.351989	1.176891	1.553137
TSFM	<0.001	0.417191	0.357568	0.486757
TSHR	0.000278	0.744949	0.635598	0.873114
TSPO	0.340879	1.110429	0.895112	1.37754
TSSK1B	2.79E-06	1.267479	1.147833	1.399596
TSSK2	2.42E-13	1.631039	1.430872	1.859208
TST	<0.001	0.565776	0.504823	0.634087
TSTA3	0.019469	0.795486	0.656562	0.963805
TTBK2	<0.001	0.271818	0.220472	0.335121
TTN	<0.001	0.283431	0.223772	0.358997
TTPA	0.000724	0.795123	0.696152	0.908164
TTR	5.83E-10	1.29285	1.191946	1.402297
TTYH1	2.60E-13	1.469745	1.32563	1.629527
TUBA1A	<0.001	0.531598	0.473071	0.597367
TUBA1B	2.22E-16	0.256761	0.185623	0.355161
TUBA1C	9.53E-06	0.49831	0.366094	0.678278
TUBA4A	<0.001	0.543494	0.478772	0.616966

(*Continued*)

Table S1: *Continued*

Gene	*P* value	HR	lower 95% CI	upper 95% CI
TUBA8	0.001075	1.245743	1.092021	1.421105
TUBAL3	6.02E-12	0.657452	0.583408	0.740895
TUBB	0.149704	0.836339	0.655814	1.066557
TUBB1	<0.001	0.414648	0.345336	0.497872
TUBB2A	5.89E-05	0.782657	0.694455	0.882061
TUBB2B	5.22E-06	0.848177	0.790171	0.910441
TUBB3	9.14E-06	1.516286	1.261585	1.822408
TUBB6	0.000114	0.771544	0.676333	0.880158
TUFM	<0.001	0.245887	0.190806	0.316867
TUT1	<0.001	0.178026	0.133138	0.238048
TWISTNB	<0.001	0.478655	0.416408	0.550206
TXK	<0.001	0.449587	0.38824	0.520629
TXLNA	0.240904	0.853694	0.655374	1.112028
TXNRD1	8.88E-16	0.532602	0.456856	0.620906
TXNRD2	<0.001	0.206569	0.155284	0.274792
TYK2	<0.001	0.26655	0.214648	0.331001
TYMP	0.488278	1.058454	0.901361	1.242925
TYMS	0.572717	1.043284	0.900446	1.20878
TYR	5.33E-15	0.390751	0.308739	0.494547
TYRO3	4.44E-16	0.417462	0.338252	0.515222
TYRP1	<0.001	0.695409	0.652093	0.741602
TYW1	<0.001	0.416552	0.362997	0.478009
UAP1	<0.001	0.508015	0.455726	0.566302
UAP1L1	0.000357	1.41674	1.170107	1.715357
UBA1	<0.001	0.303165	0.242454	0.379078
UBA2	<0.001	0.391515	0.335585	0.456768
UBA6	<0.001	0.447034	0.391564	0.510362
UBE2A	<0.001	0.334956	0.278115	0.403414
UBE2B	<0.001	0.36873	0.312694	0.434809
UBE2C	1.09E-08	1.315657	1.197536	1.445429
UBE2D1	<0.001	0.402314	0.344226	0.470206
UBE2D2	<0.001	0.354211	0.299272	0.419236
UBE2D3	<0.001	0.291636	0.241496	0.352185
UBE2D4	<0.001	0.262746	0.213596	0.323206
UBE2E1	<0.001	0.379619	0.329474	0.437395
UBE2E3	<0.001	0.427868	0.368052	0.497404
UBE2G1	<0.001	0.401563	0.347067	0.464617
UBE2G2	<0.001	0.265193	0.198673	0.353984
UBE2H	<0.001	0.302785	0.234986	0.390145
UBE2I	<0.001	0.168894	0.124722	0.22871

(*Continued*)

Table S1: *Continued*

Gene	*P* value	HR	lower 95% CI	upper 95% CI
UBE2J1	<0.001	0.36577	0.311787	0.4291
UBE2K	<0.001	0.443178	0.388652	0.505355
UBE2L3	<0.001	0.331831	0.269656	0.408343
UBE2L6	4.75E-12	0.617373	0.53847	0.707837
UBE2M	<0.001	1.829774	1.595651	2.09825
UBE2N	<0.001	0.383675	0.318594	0.46205
UBE2NL	0.112734	1.166304	0.964379	1.410509
UBE2O	1.93E-05	1.491699	1.241671	1.792073
UBE2Q1	<0.001	0.23404	0.184517	0.296853
UBE2S	<0.001	1.379024	1.290109	1.474068
UBE2W	<0.001	0.436622	0.382772	0.498049
UBE2Z	1.01E-12	0.354215	0.266285	0.471179
UBE3A	<0.001	0.303723	0.250814	0.367794
UBE3B	<0.001	0.19121	0.144319	0.253336
UBE3C	<0.001	0.302151	0.233802	0.390481
UBE4A	<0.001	0.489262	0.438483	0.545922
UBE4B	<0.001	0.342907	0.280408	0.419335
UBR2	<0.001	0.345125	0.289335	0.411674
UBR4	<0.001	0.255965	0.186595	0.351126
UBR5	<0.001	0.505391	0.454979	0.561389
UBXN4	<0.001	0.396262	0.34248	0.45849
UCKL1	0.370188	0.897271	0.70788	1.137332
UGCG	<0.001	0.534632	0.479649	0.595919
UGDH	<0.001	0.589536	0.537312	0.646835
UGGT1	<0.001	0.299227	0.242467	0.369274
UGGT2	<0.001	0.423635	0.368005	0.487676
UGP2	<0.001	0.518445	0.467808	0.574563
UGT2A3	2.58E-07	0.705786	0.618157	0.805837
UGT2B15	3.55E-15	0.605047	0.533892	0.685686
UGT2B17	0.001431	0.858337	0.781403	0.942846
UGT2B28	0.000219	0.837927	0.762922	0.920307
UGT2B4	1.35E-11	0.76543	0.708372	0.827083
UGT8	4.29E-10	0.78598	0.728748	0.847706
ULBP1	1.55E-09	0.643015	0.557155	0.742107
ULBP2	0.000299	0.787919	0.692421	0.896588
ULK1	0.011999	1.306452	1.060519	1.609417
ULK2	<0.001	0.365049	0.312087	0.427
UMPS	<0.001	0.331981	0.271894	0.405347
UNC13A	0.000148	1.31368	1.141028	1.512458
UNC13B	<0.001	0.504478	0.447373	0.568872

(*Continued*)

Table S1: *Continued*

Gene	*P* value	HR	lower 95% CI	upper 95% CI
UPB1	0.553182	0.934336	0.746466	1.169488
UPP1	2.27E-12	0.6696	0.598634	0.748978
UQCR10	6.53E-12	0.396091	0.304094	0.51592
UQCR11	<0.001	0.285322	0.212963	0.382266
UQCRB	<0.001	0.382438	0.3291	0.444421
UQCRC1	<0.001	0.473098	0.406312	0.55086
UQCRC2	<0.001	0.474503	0.421271	0.534463
UQCRFS1	<0.001	0.422619	0.350355	0.509786
UQCRQ	0.148986	0.796572	0.584891	1.084864
UROD	<0.001	0.284418	0.229892	0.351877
UROS	5.16E-14	0.379453	0.29484	0.488347
USP1	<0.001	0.561335	0.511008	0.616618
USP10	<0.001	0.448134	0.385304	0.521209
USP11	1.11E-16	0.38254	0.305201	0.479477
USP13	<0.001	0.434392	0.377409	0.499978
USP14	<0.001	0.395583	0.341577	0.458127
USP16	<0.001	0.564108	0.516021	0.616677
USP2	0.123757	1.160257	0.96018	1.402025
USP20	<0.001	0.225045	0.177001	0.286129
USP21	<0.001	0.337776	0.280912	0.406152
USP22	<0.001	0.171871	0.12402	0.238184
USP25	<0.001	0.489293	0.436222	0.54882
USP29	3.02E-07	1.476612	1.272024	1.714106
USP3	<0.001	0.435673	0.38005	0.499437
USP32	<0.001	0.438575	0.383865	0.501083
USP33	<0.001	0.4055	0.349419	0.470581
USP36	<0.001	0.226744	0.170285	0.301922
USP4	<0.001	0.477002	0.414748	0.548601
USP46	<0.001	0.369625	0.315409	0.43316
USP47	<0.001	0.487754	0.437129	0.544241
USP5	5.89E-08	1.526852	1.310263	1.779244
USP7	<0.001	0.334782	0.281579	0.398036
USP8	<0.001	0.520957	0.471236	0.575924
USP9X	<0.001	0.469162	0.418246	0.526276
USP9Y	0.068516	0.958445	0.915659	1.003229
UST	<0.001	0.603563	0.536382	0.679159
UXS1	<0.001	0.395284	0.34082	0.458451
VAMP4	<0.001	0.443321	0.391222	0.502357
VAMP7	<0.001	0.547136	0.499983	0.598736
VARS	1.19E-12	1.942224	1.617278	2.332459

(*Continued*)

Table S1: *Continued*

Gene	*P* value	HR	lower 95% CI	upper 95% CI
VASP	0.05325	0.776263	0.600451	1.003554
VAT1	0.990281	1.001639	0.769647	1.30356
VAV1	<0.001	0.473307	0.416215	0.53823
VAV2	0.423078	0.901763	0.700199	1.161352
VAV3	1.55E-15	0.603827	0.533415	0.683533
VCAM1	<0.001	0.653813	0.601961	0.710131
VCAN	1.55E-06	0.771922	0.694554	0.857908
VCP	<0.001	0.351083	0.289028	0.42646
VDAC1	0.510546	0.931511	0.754064	1.150716
VDR	<0.001	0.407349	0.336485	0.493137
VEGFA	0.632143	0.963975	0.829519	1.120224
VIL1	0.04861	0.888707	0.790369	0.99928
VIM	<0.001	0.500507	0.448304	0.55879
VKORC1	0.010043	0.718825	0.559067	0.924236
VNN1	1.67E-15	0.647395	0.581704	0.720504
VNN2	1.98E-14	0.594443	0.520299	0.679153
VNN3	5.98E-11	0.491988	0.397828	0.608434
VPS4A	<0.001	0.35142	0.289494	0.426592
VPS4B	<0.001	0.543154	0.493484	0.597824
VRK1	<0.001	0.58002	0.526684	0.638758
VRK2	<0.001	0.552812	0.496247	0.615824
VRK3	<0.001	0.2173	0.162324	0.290896
VSNL1	8.23E-09	0.784402	0.722232	0.851924
WARS	0.00231	0.777467	0.661256	0.914102
WARS2	<0.001	0.35781	0.288961	0.443064
WBSCR22	2.55E-14	2.080973	1.723426	2.512696
WDR44	<0.001	0.325189	0.263691	0.401029
WEE1	<0.001	0.461318	0.394366	0.539637
WHSC1	<0.001	0.396769	0.335864	0.468718
WHSC1L1	<0.001	0.381762	0.323762	0.450152
WIPI1	2.78E-15	0.476322	0.396295	0.57251
WNK1	<0.001	0.393107	0.331688	0.4659
WRN	<0.001	0.553606	0.501757	0.610813
WWP2	<0.001	0.241223	0.190782	0.304999
XCR1	<0.001	0.315611	0.248512	0.400829
XDH	<0.001	0.624974	0.561424	0.695718
XK	<0.001	0.736309	0.689879	0.785865
XPNPEP1	<0.001	0.32904	0.266972	0.405537
XPNPEP2	0.004869	1.223439	1.0632	1.407828
XPNPEP3	<0.001	0.27549	0.222798	0.340643

(*Continued*)

Table S1: *Continued*

Gene	*P* value	HR	lower 95% CI	upper 95% CI
XPO1	<0.001	0.503847	0.44814	0.566478
XPO4	<0.001	0.392767	0.336819	0.458008
XPO7	<0.001	0.326766	0.267884	0.39859
XPOT	<0.001	0.537689	0.484028	0.5973
XYLB	1.30E-08	0.462688	0.354736	0.603491
XYLT1	3.92E-08	0.62451	0.527961	0.738714
XYLT2	<0.001	0.26729	0.206057	0.346719
YARS	<0.001	0.34528	0.281751	0.423133
YARS2	<0.001	0.367251	0.306977	0.43936
YES1	<0.001	0.499161	0.444002	0.561173
YTHDC2	<0.001	0.437227	0.384671	0.496963
YWHAB	<0.001	0.267712	0.213497	0.335695
YWHAZ	<0.001	0.448258	0.375946	0.53448
ZAP70	4.89E-06	0.710975	0.6142	0.822997
ZBTB16	<0.001	0.696695	0.642226	0.755784
ZBTB17	5.14E-09	0.465498	0.360178	0.601614
ZCCHC11	<0.001	0.342316	0.288714	0.405869
ZCCHC6	<0.001	0.427616	0.372286	0.491169
ZDHHC13	<0.001	0.43708	0.383332	0.498363
ZDHHC14	<0.001	0.309078	0.248769	0.384008
ZDHHC17	<0.001	0.453214	0.400874	0.512388
ZDHHC18	7.70E-05	1.821036	1.35291	2.45114
ZDHHC24	1.61E-11	0.344928	0.253073	0.470121
ZDHHC3	<0.001	0.350102	0.292106	0.419614
ZDHHC4	<0.001	0.294747	0.2309	0.37625
ZDHHC6	<0.001	0.389615	0.336929	0.45054
ZDHHC7	<0.001	0.38899	0.329665	0.458991
ZFP64	<0.001	0.315513	0.252297	0.39457
ZFY	0.000309	0.870904	0.807897	0.938826
ZIC3	0.062479	1.127685	0.993757	1.279663
ZKSCAN1	<0.001	0.362895	0.306643	0.429466
ZNF146	<0.001	0.579169	0.526556	0.637039
ZNF148	<0.001	0.492131	0.440253	0.550123
ZNF219	2.29E-10	1.862052	1.536493	2.256593
ZNF224	<0.001	0.429742	0.377175	0.489635
ZNF24	<0.001	0.37561	0.321265	0.439148
ZNF33B	<0.001	0.468272	0.415844	0.527309
ZNF43	<0.001	0.539128	0.486092	0.597951

Table S2: The risk coefficients of identified 42 metabolic genes

Genes	coefficients
ABCC4	−0.03
ADCY9	−0.004
ALDH3A2	−0.01
APOA1	−0.161
ARHGDIB	−0.102
ARHGEF9	−0.04
CACNA2D1	−0.218
CAMK1D	−0.106
CCR2	−0.058
CDKL1	−0.189
COX7B	−0.012
CPA3	−0.019
CRYM	−0.076
CYTH1	−0.16
GABRA5	0.003
GAPDH	0.223
GNAO1	0.174
GNG7	−0.16
GUCY1A3	0.151
HMOX1	0.051
HMOX2	−0.199

Table S2: *Continued*

Genes	coefficients
HTR1F	−0.172
LCE2B	−0.22
LPL	−0.036
MAOA	−0.008
MAPKAPK2	−0.324
MOCS2	0.105
NTRK2	−0.061
P2RY6	0.143
PAK3	0.009
PDE3B	−0.075
PDE7B	−0.137
PRKACB	0.021
PRKCB	−0.156
PROSC	−0.122
RECQL4	0.091
S100A11	0.042
SIGLEC7	0.558
SIRT3	−0.01
TUBB3	0.018
UBE2C	0.028
ZNF43	−0.133
